# Stereoselective synthesis and transformation of pinane-based 2-amino-1,3-diols

**DOI:** 10.3762/bjoc.17.80

**Published:** 2021-05-03

**Authors:** Ákos Bajtel, Mounir Raji, Matti Haukka, Ferenc Fülöp, Zsolt Szakonyi

**Affiliations:** 1 Department of Pharmacognosy, University of Szeged, Eötvös u. 6, Szeged, 6720, Hungary; 2 Institute of Pharmaceutical Chemistry, University of Szeged, H-6720 Szeged, Eötvös u. 6, Hungary; 3 Department of Chemistry, University of Jyväskylä, POB 35, 40351 Jyväskylä, Finland; 4 Stereochemistry Research Group of the Hungarian Academy of Sciences, H-6720 Szeged, Eötvös u. 6, Hungary,; 5 Interdisciplinary Centre of Natural Products, University of Szeged, Szeged, Hungary

**Keywords:** 2-amino-1,2-diol, monoterpene, oxazolidin-2-one, stereoselective, tautomerism

## Abstract

A library of pinane-based 2-amino-1,3-diols was synthesised in a stereoselective manner. Isopinocarveol prepared from (−)-α-pinene was converted into condensed oxazolidin-2-one in two steps by carbamate formation followed by a stereoselective aminohydroxylation process. The relative stereochemistry of the pinane-fused oxazolidin-2-one was determined by 2D NMR and X-ray spectroscopic techniques. The regioisomeric spiro-oxazolidin-2-one was prepared in a similar way starting from the commercially available (1*R*)-(−)-myrtenol (**10**). The reduction or alkaline hydrolysis of the oxazolidines, followed by reductive alkylation resulted in primary and secondary 2-amino-1,3-diols, which underwent a regioselective ring closure with formaldehyde or benzaldehyde delivering pinane-condensed oxazolidines. During the preparation of 2-phenyliminooxazolidine, an interesting ring–ring tautomerism was observed in CDCl_3_.

## Introduction

**Figure 1:**
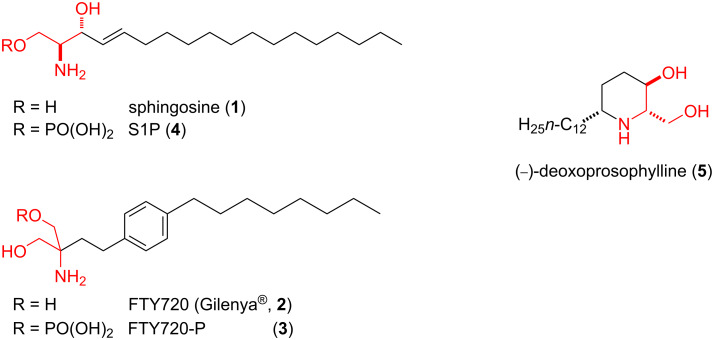
Biologically active 2-amino-1,3-diols.

The best known 2-amino-1,3-diol derivative sphingosine (**1**) plays a crucial role in intracellular signaling as second messenger, and its derivatives called sphingolipids are also critical for cell growth, cell differentiation, cell recognition, and apoptosis [1–7]. Due to its involvement in a wide range of cellular processes, significant efforts have been made in the last two decades targeting sphingosine analogues signalling as a therapeutic strategy. For instance, FTY720-P (**3**), the phosphate of FTY720 (**2**, fingolimod), proved to be a very good agonist for the S1P1 receptor (Figure 1). Sphingosine 1-phosphate (S1P, **4**), in turn, performed critical regulator functions in many physiological and pathological treatments, such as Alzheimer’s disease [8–9], cancer [10–13], multiple sclerosis [14], and inflammation [15].

Due to the lack of a readily available natural sources and the high biological importance of sphingolipid analogues, their synthesis has been the subject of numerous studies [16]. The key step for the synthesis is the stereoselective construction of the 2-amino-1,3-diol moiety of the molecules. Generally, two main synthetic strategies are used to prepare these analogues. One requires the insertion of the alcohol and amino groups in the α,β position with the correct stereochemistry [17–20]. The second strategy involves a bond formation between two chiral centers to produce the targeted 2-amino-1,3-diol [21–22]. For instance, deoxoprosophylline (**5**) as a cyclic 2-amino-1,3-diol target molecule was prepared by Kokatla et al. in an 8 step synthesis starting from Perlin aldehydes, via Pd(OH)_2_-catalyzed reductive azidoketon cyclisation [23]. Another synthetic pathway involves a stereoselective aminohydroxylation process starting from allylic carbamates usually carried out in the presence of potassium osmate [24–28].

In recent years, we have extensively studied the stereoselective synthesis, as well as catalytic and pharmacological applications of monoterpene-based 3-amino-1,2-diols, which are the regioisomers of potential monoterpenic 2-amino-1,3-diols [29–33]. These trifunctionalized terpenoids may also possess diverse biological activities and could successfully applied as chiral catalysts in enantioselective transformations [34]. In the present study, our aim was to synthesize novel, cyclic potentially analogues of sphingosine, incorporating a lipophilic natural pinane skeleton, starting from commercially available monoterpene-based allylic alcohols via a stereoselective hydroxyamination in the presence of a potassium osmate(VI) catalyst. We also planned to explore the regioselectivity of the ring closure of the resulting 2-amino-1,3-diols to obtain promising 1,3-heterocycles. To reach our goal, (1*S*)-(−)-α-pinene (**6**) and (1*R*)-(−)-myrtenol (**10**), two naturally occurring monoterpenoids were selected as precursors, as both are commercially available, cheap starting materials.

## Results and Discussion

### Synthesis of regioisomeric oxazolidinones from (1*S*)-(−)-α-pinene (**6**) and (1*R*)-myrtenol (**10**)

**Scheme 1:**
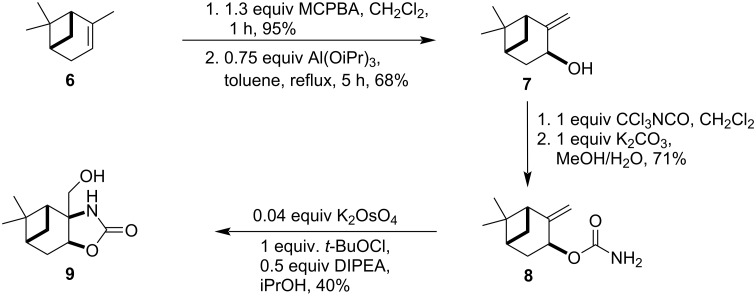
Stereoselective synthesis of the pinane-fused oxazolidin-2-one **9**.

The synthesis of isopinocarveol (**7**), the key intermediate allylic alcohol, was performed according to a literature procedure in good yield [35]. The first step was the stereoselective epoxidation of (−)-α-pinene (**6**), carried out with *meta*-chloroperoxybenzoic acid (MCPBA), followed by a base-catalyzed allylic rearrangement mediated by aluminium isopropoxide (Al(OiPr)_3_). The resulting allylic alcohol **7** was reacted with trichloroacetyl isocyanate, followed by alkaline treatment, delivering carbamate **8** in good yield [27–28,36]. In the next step, the aminohydroxylation was accomplished by potassium osmate(VI) as the catalyst and *t*-BuOCl in the presence of DIPEA affording oxazolidine-2-one **9** [27]. The reaction was found to be highly stereoselective, giving exclusively the *diexo*-fused tricyclic **9** ring system (Scheme 1).

**Figure 2:**
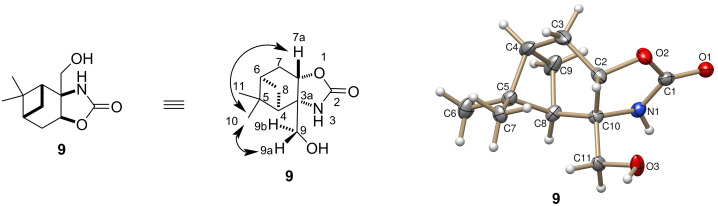
NOESY experiments and X-ray structure elucidation of oxazolidin-2-one **9**.

The absolute configuration of compound **9** was determined by 2D NMR spectroscopic techniques. Clear NOE signals were observed between the H-7a and Me-10 as well as the H_a_-9 and Me-10 protons. Beside NOESY experiments, the structure was also elucidated by X-ray crystallography (Figure 2).

**Scheme 2:**
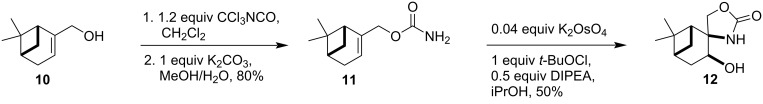
Stereoselective synthesis of the pinane spiro-fused oxazolidin-2-one **12**.

**Scheme 3:**
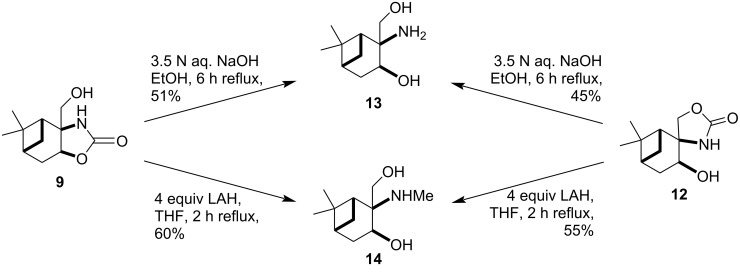
Parallel synthesis of 2-amino-1,3-diols.

To synthesize the regioisomeric spiro-oxazolidinone derivative **12**, (1*R*)-(−)-myrtenol (**10**) was chosen as starting material (Scheme 2). The synthetic method was similar to that mentioned above for (−)-isopinocarveol. In the first step, carbamate **11** was prepared [37], then the aminohydroxylation was carried out catalyzed by potassium osmate(VI), which led to the formation of the spiro-oxazolidine-2-one **12** in a highly regio- and stereoselective manner. Based on the NMR spectroscopic measurements of the crude product, the spiro derivative **12** was obtained exclusively with the relative configuration depicted in Scheme 2. Beside 2D NMR spectroscopic studies, the absolute configuration of compound **12** was determined by its transformation into the corresponding aminodiols **13** and **14**, comparing the products with those obtained from the regioisomer **9** (discussed in Scheme 3).

### Synthesis and transformations of pinane-based 2-amino-1,3-diols

To obtain a library of pinane-based 2-amino-1,3-diols, the oxazolidine-2-ones **9** and **12** were applied as starting materials. The alkaline hydrolysis of both **9** and **12** resulted in the same primary aminodiol **13** [38]. According to the NMR spectra and other physical and chemical properties, there was no difference between the products of the two reactions. Since the relative configuration of compound **9** was clarified by NMR spectroscopy and X-ray crystallographic results, we were able to assign the stereochemistry of spiro-derivate **12**, too. In a similar manner, the LiAlH_4_ (LAH) reduction of both **9** and **12** gave the same *N*-methylaminodiol **14** with modest yield (Scheme 3).

**Scheme 4:**
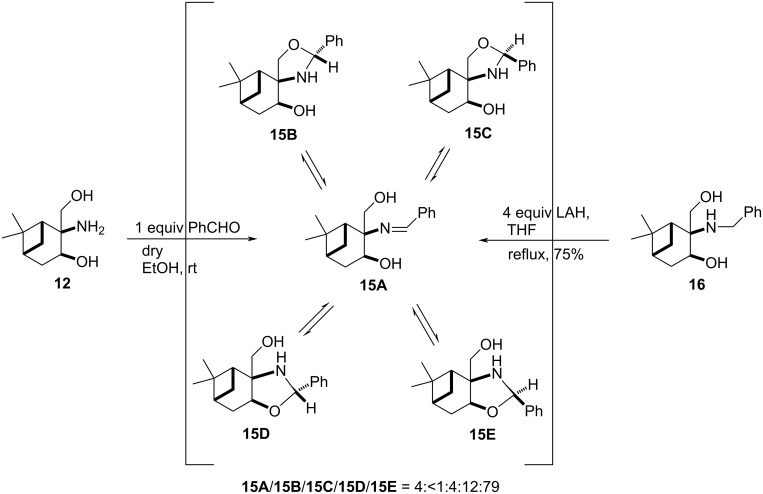
Synthesis of *N*-benzyl-2-amino-1,3-diol **16**.

Subsequently, compound **13** was reacted with benzaldehyde. In this process, the Schiff' base **15A** was generated in situ. Our efforts to reduce it with sodium borohydride failed, since we did not observe the formation of the expected *N*-benzylaminodiol either at room temperature or under reflux conditions, probably due to the strong steric hindrance of the bicyclic system and the hydroxymethyl group. The ^1^H NMR spectroscopic measurements in CDCl_3_ clearly showed that the crude product was a five-component tautomeric mixture containing condensed oxazolidine **15E** as the main component. Additional minor components included the other condensed oxazolidine (**15D**), spiro compounds **15B** and **15C** as well as the Schiff’ base **15A** existing in a ratio of **15A**/**15B**/**15C**/**15D**/**15E** = 4:<1:4:12:79 (Scheme 4) [39–40]. The structures of the five components **15A–E** were determined by 2D NMR spectroscopic techniques (NOESY and HMBC). Since this finding is quite unusual in the case of Schiff’ bases, we decided to study the ring/chain tautomeric mixture (**15A–E**) in the reaction of **13** with benzaldehyde by ^1^H NMR spectroscopy. When a time-dependent ^1^H NMR spectroscopic measurement was accomplished, we observed that the equilibrium composition was established rapidly, without any significant change in the ratio of the tautomers. The equilibrium shifting strongly to product **15E** can account of the difficulty of the reduction process and the necessity to use a stronger reducing agent and more severe conditions. The reduction step, therefore, was performed by applying LAH, a stronger reducing agent, and longer reflux, resulting in *N*-benzyl-2-amino-1,3-diol **16** (Scheme 4).

**Scheme 5:**
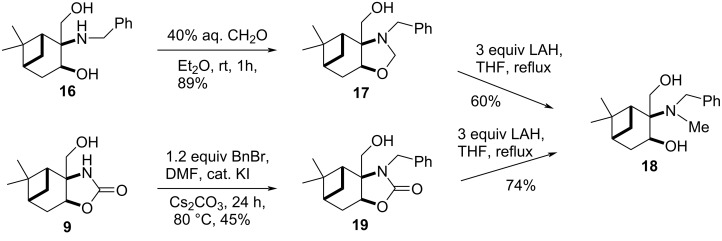
Synthesis of 2-amino-1,3-diols.

**Figure 3:**
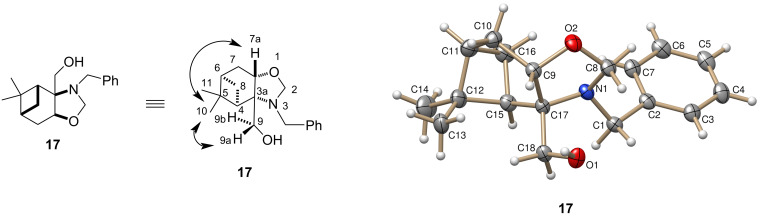
NOESY experiments and X-ray structure proofment of the structure of oxazolidine **17**.

When compound **16** was treated with formaldehyde at room temperature, pinane-fused oxazolidine **17** was obtained regioselectively (Scheme 5), as it was indicated by clear HMBC correlations between the CH_2_ of the oxazolidine ring and the anellation carbons, in contrast to the results observed in the case of the regioisomeric 3-amino-1,2-diols, where spiro-oxazolidines formed exclusively [41]. The configuration of oxazolidine **17** was determined by 2D NMR spectroscopic techniques. Clear NOE signals were observed between the H-7a and Me-10 as well as the H_a_-9 and Me-10 protons. In addition to NOESY experiments, the structure was also elucidated by X-ray crystallography (Figure 3).

The LAH reduction of oxazolidine **17** gave *N*-benzyl-*N*-methyl analogue **18** which, alternatively, was prepared directly from 2-oxazolidinone **9** via *N*-benzylation followed by LAH reduction in 2 steps.

When compound **13** was reacted with phenylisothiocyanate, thiourea **20** was obtained, which underwent a regioselective ring closure resulting in **21A**. The structure of **21A** was determined by ^1^H (whereas the CH-*OH* gave a dublet in DMSO-*d*_6_ while the CH_2_-*OH* of **21B** could be detected as triplet) and 2D NMR spectroscopic techniques (HMBC). It is important to mention that this regioselectivity is the opposite to that observed in the reaction of aminodiols **13** and **16** with aldehydes (see Scheme 4 and Scheme 5), but it is similar to that observed in our earlier study with pinane-based 3-amino-1,2-diols [41]. During the NMR spectroscopic study of **21A** in CDCl_3_ for 30 days, an unknown slow ring–ring tautomerization was observed, forming a 1:1 mixture of the two regioisomers **21A** and **21B**. Compound **21B** could be isolated from the mixture by column chromatography in pure form.

**Scheme 6:**
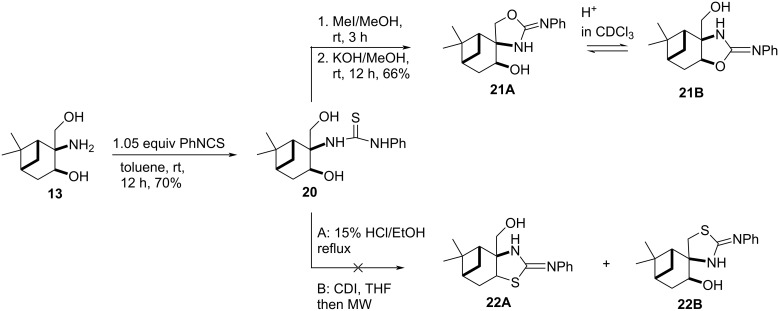
Synthesis of 2-phenyliminooxazolidines.

The synthesis of the heteroanalogue 2-phenyliminothiazolidines **22A** and **22B** failed, even when the reaction was attempted under acidic or even milder conditions (Scheme 6).

**Figure 4:**
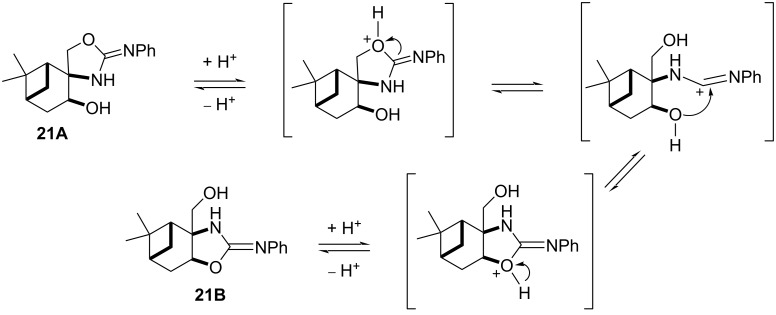
Proposed pathway for the ring–ring tautomerism.

The proposed reaction pathway for the ring–ring tautomerism of **21A** and **21B** is presented in Figure 4 and it explains why the acidic environment (present generally in CDCl_3_ solution) is necessary. In a similar manner, an oxazolidine–1,3-oxazine tautomerism of pulegone-based 3-amino-1,2-diols was recently reported [31]. When compound **21A** or **21B** were treated in less protic solvents such as DMSO-*d*_6_ or CD_3_OD, tautomerization was not observed.

## Conclusion

A small library of pinane-based 2-amino-1,3-diols was synthesized in a stereoselective manner starting from (1*R*)-(−)-myrtenol and isopinocarveol prepared from α-pinene. Pinane-condensed or spiro-oxazolidin-2-ones were formed in three steps by a stereoselective hydroxyamination process. The relative stereochemistry of new compounds was determined by 2D NMR spectroscopic and X-ray techniques. The resulting primary and secondary 2-amino-1,3-diols underwent a regioselective ring closure with formaldehyde and benzaldehyde producing pinane-condensed oxazolidines. In the case of 2-phenyliminooxazolidine, an interesting ring–ring tautomerism was observed in CDCl_3_. The prepared trifunctional compounds may serve as chiral catalysts in enantioselective transformations, while the 2-phenyliminooxazolidines could be interesting in the field of antiproliferative or antioxidants studies based on our former studies on 2-imino-1,3-heterocycles [42–43].

## Supporting Information

File 1 Experimental part, analytical data, NMR spectra and X-ray data of the prepared compounds.

## References

[1] Posse de Chaves E, Sipione S (2010). FEBS Lett.

[2] Hannun Y A, Obeid L M (2002). J Biol Chem.

[3] Kågedal K, Zhao M, Svensson I, Brunk U T (2001). Biochem J.

[4] Kolesnick R N, Goñi F M, Alonso A (2000). J Cell Physiol.

[5] Perry D K, Hannun Y A (1998). Biochim Biophys Acta, Mol Cell Biol Lipids.

[6] Igarashi Y (1997). J Biochem.

[7] Spiegel S, Foster D, Kolesnick R (1996). Curr Opin Cell Biol.

[8] Takasugi N, Sasaki T, Suzuki K, Osawa S, Isshiki H, Hori Y, Shimada N, Higo T, Yokoshima S, Fukuyama T (2011). J Neurosci.

[9] Prager B, Spampinato S F, Ransohoff R M (2015). Trends Mol Med.

[10] Heffernan-Stroud L A, Obeid L M (2013). Sphingosine Kinase 1 in Cancer. Advances in Cancer Research.

[11] Pyne N J, Tonelli F, Lim K G, Long J S, Edwards J, Pyne S (2012). Biochem Soc Trans.

[12] Plano D, Amin S, Sharma A K (2014). J Med Chem.

[13] Pyne N J, Pyne S (2010). Nat Rev Cancer.

[14] Chi H (2011). Trends Pharmacol Sci.

[15] Maceyka M, Spiegel S (2014). Nature.

[16] Howell A R, So R C, Richardson S K (2004). Tetrahedron.

[17] Cardillo G, Orena M, Porzi G, Sandri S (1981). J Chem Soc, Chem Commun.

[18] Azuma H, Takao R, Niiro H, Shikata K, Tamagaki S, Tachibana T, Ogino K (2003). J Org Chem.

[19] He L, Byun H-S, Bittman R (2000). J Org Chem.

[20] Sasai H, Tokunaga T, Watanabe S, Suzuki T, Itoh N, Shibasaki M (1995). J Org Chem.

[21] Masui M, Shioiri T (1998). Tetrahedron Lett.

[22] Ma N, Ma D (2003). Tetrahedron: Asymmetry.

[23] Kokatla H P, Lahiri R, Kancharla P K, Doddi V R, Vankar Y D (2010). J Org Chem.

[24] Han H, Cho C-W, Janda K D (1999). Chem – Eur J.

[25] Rudolph J, Sennhenn P C, Vlaar C P, Sharpless K B (1996). Angew Chem, Int Ed Engl.

[26] Donohoe T J, Johnson P D, Helliwell M, Keenan M (2001). Chem Commun.

[27] Donohoe T J, Johnson P D, Cowley A, Keenan M (2002). J Am Chem Soc.

[28] Hovey M T, Eklund E J, Pike R D, Mainkar A A, Scheerer J R (2011). Org Lett.

[29] Szakonyi Z, Hetényi A, Fülöp F (2008). Tetrahedron.

[30] Csillag K, Németh L, Martinek T A, Szakonyi Z, Fülöp F (2012). Tetrahedron: Asymmetry.

[31] Gonda T, Szakonyi Z, Csámpai A, Haukka M, Fülöp F (2016). Tetrahedron: Asymmetry.

[32] Szakonyi Z, Csőr Á, Csámpai A, Fülöp F (2016). Chem – Eur J.

[33] Le T M, Csámpai A, Fülöp F, Szakonyi Z (2018). Chem – Eur J.

[34] El Alami M S I, El Amrani M A, Agbossou-Niedercorn F, Suisse I, Mortreux A (2015). Chem – Eur J.

[35] Lavallee P, Bouthillier G (1986). J Org Chem.

[36] Miller K E, Wright A J, Olesen M K, Hovey M T, Scheerer J R (2015). J Org Chem.

[37] Kamon T, Shigeoka D, Tanaka T, Yoshimitsu T (2012). Org Biomol Chem.

[38] Byun H-S, Bittman R (2012). Chem Phys Lipids.

[39] Lázár L, Fülöp F (2003). Eur J Org Chem.

[40] Hetényi A, Szakonyi Z, Klika K D, Pihlaja K, Fülöp F (2003). J Org Chem.

[41] Szakonyi Z, Hetényi A, Fülöp F (2007). ARKIVOC.

[42] Szakonyi Z, Zupkó I, Sillanpää R, Fülöp F (2014). Molecules.

[43] Firpo G, Ramírez M L, Faillace M S, Mendes de Brito M d R, Correia Lima e Silva A P S, Pereira Costa J, Rodríguez M C, Argüello G A, Szakonyi Z, Fülöp F (2019). Antioxidants.

