# Supplementary material for: Stereoselective synthesis and transformation of pinane-based 2-amino-1,3-diols
Source: Beilstein J Org Chem. 2021 May 3;17:983–90. doi: 10.3762/bjoc.17.80 (PMC8111431; doi:10.3762/bjoc.17.80)

BEILSTEIN JOURNAL OF ORGANIC CHEMISTRY

Supporting Information

for

Stereoselective synthesis and transformation of pinane-based 2-amino-1,3-diols

Ákos Bajtel, Mounir Raji, Matti Haukka, Ferenc Fülöp and Zsolt Szakonyi

Beilstein J. Org. Chem. **2021,** *17,* 983–990. doi:10.3762/bjoc.17.80

Experimental part, analytical data, NMR spectra and X-ray data of the prepared compounds

License and Terms: This is a supporting information file under the terms of the Creative Commons Attribution License (https://creativecommons.org/licenses/by/4.0). Please note that the reuse, redistribution and reproduction in particular requires that the author(s) and source are credited and that individual graphics may be subject to special legal provisions.
The license is subject to the *Beilstein Journal of Organic Chemistry* terms and conditions: (https://www.beilstein-journals.org/bjoc/terms)

Table of contents

Experimental part S2–S8

X-ray data of compounds **9** and **17** S8–S9

^{1}H, ^{13}C NMR, COSY, NOESY, HSQC and HMBC spectra of new compounds S10–S38

Time-dependent tautomerisation of **21A** to **21B** in $CDCl_3$ solution S39

Experimental

^{1}H and ^{13}C NMR spectra were recorded on Bruker Avance DRX 400 or Bruker Avance DRX 500 spectrometer [δ = 0 (TMS)] (Bruker Corp., Billerica, MA, USA) in solvents as indicated. Chemical shifts are expressed in ppm (δ) relative to TMS as internal reference. J values are given in Hz. Elemental analyses were performed on a Perkin-Elmer 2400 Ser II Elemental Analyzer (PerkinElmer Inc., Waltham, MA, USA). HRMS flow injection analysis was performed with a Thermo Scientific Q Exactive Plus hybrid quadrupole-Orbitrap (Thermo Fisher Scientific, Waltham, MA, USA) mass spectrometer coupled to a Waters Acquity I-Class UPLC™ (Waters, Manchester, UK). Optical rotations were measured with a Perkin-Elmer 341 polarimeter (PerkinElmer Inc., Shelton, CT, USA). Melting points were determined on a Kofler apparatus (Nagema, Dresden, Germany). Chromatographic separations were carried out on Merck Kieselgel 60 (230–400 mesh ASTM, Merck Ltd., Budapest, Hungary). Reactions were monitored with Merck Kieselgel 60 F254-precoated TLC plates (0.25 mm thickness). All chemicals and solvents were used as supplied. (1*R*)-(−)-Myrtenol (**10**) (*ee* > 95%) and (1*S*,5*S*)-(−)-(α)-pinene (*ee* > 95%) were obtained from Merck Hungary Co. Isopinocarveol (**7**) was prepared from commercially available (1*S*,5*S*)-(−)-(α)-pinene (**6**) using a literature method [1], with all spectroscopic data and physical properties similar to those reported therein.

General procedure for carbamate formation

Trichloroacetyl isocyanate (6.78 g, 35.99 mmol) was added dropwise to a solution of the allylic alcohol (4.50 g, 29.56 mmol) in dry DCM (50 mL) at 0 °C. After stirring for 2 h, the mixture was concentrated under reduced pressure and the residue was dissolved in MeOH (60 mL). An aqueous solution of K_2CO_3 (4.30 g in 15 mL of H_2O) was added to the solution at 0 °C and the mixture was allowed to stir for 4 h (TLC monitoring). The MeOH was evaporated under reduced pressure and the aqueous residue was extracted with DCM (3 × 50 mL). The combined organic phase was dried (Na_2SO_4), filtered, and concentrated under reduced pressure to yield the crude carbamate, which was purified by column chromatography on silica gel (*n*-hexane/EtOAc 9:1).

(1*R*,3*S*,5*R*)-6,6-Dimethyl-2-methylenebicyclo[3.1.1]heptan-3-yl carbamate (8)

Compound **8**: synthesis was performed starting from (−)-isopinocarveol, 4.18 g (72%), white crystalline powder, mp: 113–115 °C, $[\alpha]_D^{20}$ = +12.5 (c = 0.315, MeOH), ^{1}H NMR (500.2 MHz, $CDCl_3$): δ = 0.69 (s, 3H), 1.28 (s, 3H), 1.55 (d, 1H, *J* = 10.2 Hz), 1.86 (dd, 1H, *J* = 3.9, 15.3 Hz), 1.96-2.06 (m, 1H), 2.35-2.46 (m, 2H), 2.53 (t, 1H, *J* = 5.5 Hz), 4.75 (br s, 2H), 4.91 (s, 1H), 5.11 (s, 1H), 5.44 (d, 1H, *J* = 8.1 Hz), ^{13}C NMR (125.8 MHz, $CDCl_3$): δ = 22.0, 25.9, 27.9, 33.4, 39.6, 40.5, 50.8, 69.4, 114.0, 150.5, 156.8. Anal. calcd for $C_{11}H_{17}NO_2$ (195.26): C, 67.66; H, 8.78; N, 7.17; Found: C, 67.42; H, 8.80; N, 7.52. HRMS-ESI [M+H]+*m/z* found 196.13276, calcd for $C_{11}H_{18}NO_2$: 196.13375.

[(1*R*,5*S*)-6,6-Dimethylbicyclo[3.1.1]hept-2-en-2-yl]methyl carbamate (11)

Compound **11**: synthesis was performed starting from (1*R*)-(−)-myrtenol, 4.65 g (80%), white crystals, all physical and chemical properties are similar to those reported in the literature,[2] $[\alpha]_D^{20}$ = –43 (c = 0.265, MeOH), Anal. calcd for $C_{11}H_{17}NO_2$ (195.26): C, 67.66; H, 8.78; N, 7.17; Found: C, 67.42; H, 8.53; N, 7.35. HRMS-ESI [M+H]+*m/z* found 196.13289, calcd for $C_{11}H_{18}NO_2$: 196.13375.

General method for the aminohydroxylation process

To a solution of allylic carbamate **8** or **11** (2.00 g, 10.24 mmol) in iPrOH (180 mL), freshly prepared 0.33% aqueous solution of NaOH (80 mL) was added. The solution was allowed to stir for 5 min, then *t*-BuOCl (1.026 mL, 10.30 mmol) was added. After stirring for 5 min, *N,N*-diisopropylethylamine (59 mg, 79.5 µL, 0.46 mmol) and potassium osmate(VI) dihydrate (135 mg, 0.366 mmol) were added to the solution in one portion. After the addition of 0.33% aqueous NaOH solution (20 mL), the mixture was stirred for 24 h (TLC monitoring), then quenched with Na_2SO_3 (500 mg) and allowed to stir for 30 min. The mixture was extracted with EtOAc (3 × 50 mL) and the organic layer was washed with brine (1 × 50 mL), dried (Na_2SO_4), filtered and concentrated under reduced pressure to yield the crude product, which was purified by column chromatography on silica gel (*n*-hexane/EtOAc 1:2).

(3a*S*,4*R*,6*R*,7a*S*)-3a-Hydroxymethyl-5,5-dimethylhexahydro-4,6-methanobenzo[*d*]oxazol-2(3*H*)-one (9)

Compound **9**: 0.86 g (40%), white crystalline powder, mp: 195–197 °C, $[\alpha]_D^{20}$ = +31 (c = 0.290, MeOH), ^{1}H NMR (400 MHz, DMSO-d$_6$): δ = 0.80 (s, 3H), 1.00-1.10 (m, 1H), 1.20 (s, 3H), 1.33 (d, 1H, *J* = 9.9 Hz), 1.58 (dt, 1H, *J* = 3.6, 14.1 Hz), 1.81-1.87 (m, 1H), 1.99 (t, 1H, *J* = 5.8 Hz), 2.08-2.16 (m, 1H), 2.26-2.33 (m, 1H), 3.97-4.02 (m, 1H), 4.03 (d, 1H, *J* = 8.6 Hz), 4.16 (d, 1H, *J* = 8.6 Hz), 5.07 (d, 1H, *J* = 5.0 Hz), 7.69 (br s, 1H), ^{13}C NMR (100 MHz, DMSO-d$_6$): δ = 23.4, 27.2, 27.4, 37.3, 38.6, 39.8, 53.0, 65.6, 68.9, 76.8, 158.4. Anal. calcd for $C_{11}H_{17}NO_3$ (211.26) : C, 62.54; H, 8.11; N, 6.63; Found: C, 62.28; H, 8.47; N, 6.23. HRMS-ESI [M+H]+*m/z* found 212.12775, calcd for $C_{11}H_{18}NO_3$: 212.12867.

(1*R*,2*S*,3*S*,5*R*)-3-hydroxy-6,6-dimethylspiro[bicyclo[3.1.1]heptane-2,4'-oxazolidin]-2'-one (12)

Compound **12**: 1.08 g (50%), white crystalline powder, mp: 153–156 °C, $[\alpha]_D^{20}$ = –55 (c = 0.285, MeOH), ^{1}H NMR (400 MHz, DMSO-d$_6$): δ = 0.80 (s, 3H), 1.20 (d, 1H, *J* = 11.1 Hz), 1.23 (s, 3H), 1.75-1.83 (m, 2H), 1.85-1.90 (m, 1H), 2.21-2.28 (m, 1H), 2.30-2.38 (m, 1H), 3.21 (dd, 1H, *J* = 5.7, 11.5 Hz), 3.41 (dd, 1H, *J* = 5.8, 11.7 Hz), 4.64 (d, 1H, *J* = 8.1 Hz), 5.05 (t, 1H, *J* = 5.8 Hz), 7.43 (br s, 1H), ^{13}C NMR (100 MHz, DMSO-d$_6$): δ = 24.3, 26.7, 27.3, 34.8, 38.5, 39.4, 65.7, 65.8, 71.4, 158.1. Anal. calcd for $C_{11}H_{17}NO_3$ (211.26) : C, 62.54; H, 8.11; N, 6.63; Found: C, 62.36; H, 8.27; N, 6.41. HRMS-ESI [M+H]+*m/z* found 212.12775, calcd for $C_{11}H_{18}NO_3$: 212.12867.

General method for the alkaline hydrolysis of oxazolidinones

To a solution of oxazolidinone **9** or **12** (1.70 g, 8.05 mmol) in dry EtOH (21 mL), 14% aqueous solution of NaOH (10 mL) was added and the mixture was heated under reflux for 6 h. The solution was evaporated to approx. 10 mL volume, then extracted with DCM (3 × 30 mL). The combined organic layer was dried (Na_2SO_4), filtered and concentrated under reduced pressure to yield crude product **13**, which was purified as a hydrochloride salt by recrystallization from an EtOH/Et_2O mixture.

(1*R*,2*S*,3*S*,5*R*)-2-Amino-2-hydroxymethyl-6,6-dimethylbicyclo[3.1.1]heptan-3-ol hydrochloride (13)

Compound **13**: 0.91 g (51%) from **9** and 0.80 g (45%) from **12**, white crystalline powder, mp: 195–196 °C, $[\alpha]_D^{20}$= +7 (c = 0.270, MeOH), ^{1}H NMR (400 MHz, DMSO-d$_6$): δ = 0.92 (s, 3H), 1.23 (s, 3H), 1.50 (d, 1H, *J* = 10.6 Hz), 1.68-1.75 (m, 1H), 1.84-1.89 (m, 1H), 2.13-2.20 (m, 1H), 2.27 (t, 1H, *J* = 5.5 Hz), 2.31-2.37 (m, 1H), 3.43 (dd, 1H, *J* = 4.8, 11.6 Hz), 3.61 (dd, 1H, *J* = 5.6, 11.7 Hz), 3.99 (dd, 1H, *J* = 5.2, 9.0 Hz), 5.48 (t, 1H, *J* = 5.5 Hz), 5.64 (br s, 1H), 7.65 (br s, 3H), ^{13}C NMR (400 MHz, DMSO-d$_6$): δ = 24.3, 27.1, 27.7, 37.7, 39.2, 40.4, 45.9, 63.3, 64.9. Anal. calcd for $C_{10}H_{20}ClNO_2$ (221.72): C, 54.17; H, 9.09; N, 6.32; Found: C, 54.43; H, 9.27; N, 6.45. HRMS-ESI [M+H]+*m/z* found 186.14915, calcd for $C_{10}H_{20}NO_2$: 186.14940.

General method for the LAH reduction of oxazolidinones 9 and 12

To the stirred suspension of $LiAlH_4$ (0.25 g, 6.59 mmol) in dry THF (5 mL) the solution of **9** or **12** (0.35 g, 1.656 mmol) in dry THF (5 mL) was added dropwise under ice cooling. The reaction mixture was treated under reflux conditions for 2 h, then a mixture of H_2O (0.50 mL) and THF (5 mL) was added dropwise with cooling. After 30 min of stirring, the inorganic material was filtered off and washed with THF (3 × 30 mL). The filtrate was dried (Na_2SO_4), filtered and evaporated. The obtained crude product was purified by column chromatography on silica gel (toluene/EtOH 1:1).

(1*R*,2*S*,3*S*,5*R*)-2-Hydroxymethyl-6,6-dimethyl-2-methylaminobicyclo[3.1.1]heptan-3-ol (14)

Compound **12**: 0.20 g (60%) from **9** and 0.18 g (55%) from **12**, white crystalline powder, mp: 126–128 °C, $[\alpha]_D^{20}$= –3 (c = 0.200, MeOH), ^{1}H NMR (400 MHz, DMSO-d$_6$): δ = 0.91 (s, 3H), 1.21 (s, 3H), 1.45 (d, 1H, *J* = 9.3 Hz), 1.50-1.56 (m, 1H), 1.77-1.81 (m, 1H), 1.83-1.87 (m, 1H), 2.02-2.06 (m, 1H), 2.08 (s, 3H), 2.26-2.33 (m, 1H), 3.22 (d, 2H, *J* = 11.0 Hz), 3.35 (d, 1H, *J* = 11.2 Hz), 4.01 (dd, 1H, *J* = 5.7, 9.1 Hz), ^{13}C NMR (400 MHz, DMSO-d$_6$): δ = 24.9, 27.6, 28.1, 28.8, 38.4, 38.8, 40.4, 46.6, 61.5, 64.8, 66.8. Anal. calcd for $C_{11}H_{21}NO_2$ (199.29): C, 66.29; H, 10.62; N, 7.03; Found: C, 66.53; H, 10.27; N, 7.33. HRMS-ESI [M+H]+*m/z* found 200. 16391, calcd for $C_{11}H_{22}NO_2$: 200.16451.

Preparation of the 15A–E tautomeric mixture

Aminodiol **13** (20.0 mg, 0.11 mmol) and benzaldehyde (10.2 µL, 10.6 mg, 0.11 mmol) were dissolved in dry ethanol (2 mL) and stirred for 2 h at room temperature. Then, the solvent was evaporated under reduced pressure to afford the mixture of **15A–E** that was examined in $CDCl_3$.

Compounds **15A–E**: 0.030 g (98%) crude mixture. Ratio of **15A**:**15B**:**15C**:**15D**:**15E**: 4:<1:4:12:79 based on ^{1}H NMR and 2D NMR (NOESY and HMBC) measurement. Characteristic chemical shifts for the C-2 methyne peaks of tautomers: ^{1}H NMR (500.2 MHz, $CDCl_3$): δ = 8.02 (d, *J* = 6.5 Hz): **15A**, 5.90 (s): **15B**/**15C**, 5.41 (s): **15D**, 5.30 (s): **15E**.

(1*R*,2*S*,3*S*,5*R*)-2-Benzylamino-2-hydroxymethyl-6,6-dimethylbicyclo[3.1.1]heptan-3-ol (16)

To a solution of aminodiol **14** (liberated base, 0.60 g, 3.24 mmol) in dry ethanol (20 mL) benzaldehyde (0.343 g, 0.328 mL, 3.24 mmol) was added in one portion, and the solution was stirred at room temperature for 1 h and then evaporated to dryness. The residue was dissolved in dry THF (3 mL) and then added dropwise to the stirred suspension of dry THF (10 mL) and $LiAlH_4$ (0.491 g, 12.95 mmol). The reaction mixture was treated under reflux conditions for 3 h, then a mixture of H_2O (1.00 mL) and THF (8 mL) was added dropwise with cooling. After stirring for 30 min, the inorganic material was filtered off and washed with THF (3 × 30 mL). The filtrate was dried (Na_2SO_4), filtered and evaporated. The obtained crude product was purified by column chromatography on silica gel ($CHCl_3$/MeOH 9:1).

Compound **16**: 0.66 g (75%), white crystalline powder, mp: 112–114 °C, $[\alpha]_D^{20}$= +10.8 (c = 0.320, MeOH), ^{1}H NMR (400 MHz, DMSO-d_6): δ = 0.95 (s, 3H), 1.24 (s, 3H), 1.57 (dd, 1H, *J* = 5.5, 13.6 Hz), 1.65 (d, 1H, *J* = 9.5 Hz), 1.80-1.85 (m, 1H), 1.98 (t, 1H, *J* = 5.7 Hz), 2.07-2.14 (m, 1H), 2.28-2.35 (m, 1H), 3.30-3.33 (m, 2H, overlapped with H_2O peak), 3.48 (d, 1H, *J* = 10.9 Hz), 3.65 (d, 1H, *J* = 12.4 Hz), 4.11 (dd, 1H, *J* = 6.4, 8.6 Hz), 4.32 (br s, 2H), 7.18-7.37 (m, 5H), ^{13}C NMR (400 MHz, DMSO-d_6): δ = 25.0, 27.9, 29.0, 38.5, 38.7, 40.5, 45.3, 47.1, 47.4, 61.9, 66.0, 67.1, 126.9, 128.5, 128.6, 142.4. Anal. calcd for $C_{17}H_{25}NO_2$ (275.39): C, 74.14; H, 9.15; N, 5.09; Found: C, 74.47; H, 9.31; N, 5.35. HRMS-ESI [M+H]+*m/z* found 276.19634, calcd for $C_{17}H_{26}NO_2$: 276.19635.

((3a*S*,4*R*,6*R*,7a*S*)-3-Benzyl-5,5-dimethyloctahydro-4,6-methanobenzo[*d*]oxazol-3a-yl)methanol (17)

In a manner similar to a procedure in [3], to the solution of **16** (0.25 g, 0.91 mmol) in Et_2O (10 mL), 40% aqueous formaldehyde solution (4 mL) was added. The reaction mixture was stirred for 1 h at room temperature followed by making it alkaline with 10% cold aqueous KOH solution (5 mL) and extracted with Et_2O (3 × 30 mL). The combined organic layer was washed with saturated NaCl solution (2 × 20 mL) then dried (Na_2SO_4), filtered and evaporated to dryness. The crude product was purified by column chromatography on silica gel (*n*-hexane/EtOAc 3:2).

Compound **17**: 0.23 g (89%), pale-yellow crystalline powder, mp: 77–78 °C, $[\alpha]_D^{20}$ = +3 (c = 0.285, MeOH), ^{1}H NMR (400 MHz, $CDCl_3$): δ = 0.93 (s, 3H), 1.30 (s, 3H), 1.45 (d, 1H, *J* =

10.2 Hz), 1.86-1.95 (m, 2H), 2.08-2.14 (m, 1H), 2.35-2.43 (m, 1H), 2.48-2.55 (m, 1H), 3.13 (br s, 1H), 3.27 (br d, 1H, *J* = 9.5 Hz), 3.60 (br d, 1H, *J* = 10.9 Hz), 3.67 (br d, 1H, *J* = 13.7 Hz), 3.86 (br d, 1H, *J* = 12.5 Hz), 4.44 (s, 1H), 4.50 (d, 1H, *J* = 2.8 Hz), 4.56 (d, 1H, *J* = 8.6 Hz), 7.21-7.35 (m, 5H), ^{13}C NMR (400 MHz, $CDCl_3$): δ = 24.6, 27.2, 27.6, 35.7, 39.1, 47.0, 48.9, 65.3, 76.3, 84.5, 127.3, 128.1, 128.6, 139.6. Anal. calcd for $C_{18}H_{25}NO_2$ (287.40): C, 75.22; H, 98.77; N, 4.87; Found: C, 75.54; H, 98.39; N, 4.61. HRMS-ESI [M+H]+*m/z* found 288.19544, calcd for $C_{18}H_{26}NO_2$: 288.19581.

(1*R*,2*S*,3*S*,5*R*)-2-(*N*-Benzyl-*N*-methylamino)-2-hydroxymethyl-6,6 dimethylbicyclo[3.1.1]heptan-3-ol (18)

To the stirred suspension of $LiAlH_4$ (0.166 g, 4.38 mmol) in dry THF (5 mL) the solution of **17** or **19** (1.46 mmol) in dry THF (5 mL) was added dropwise at room temperature. After a 12 h treatment under reflux conditions a mixture of H_2O (0.30 mL) and THF (4 mL) was added dropwise with cooling to the reaction mixture. After 30 min of stirring, the inorganic material was filtered off and washed with THF (3 × 20 mL). The filtrate was dried (Na_2SO_4), filtered, and evaporated. The obtained crude product was purified by column chromatography on silica gel (DCM/MeOH 19:1).

Compound **18**: 0.26 g (60%) from **17**, 0.32 g (74%) from **19**, colourless oil, $[\alpha]_D^{20}$ = –40 (c = 0.365, MeOH), ^{1}H NMR (400 MHz, $CDCl_3$): δ = 1.03 (s, 3H), 1.35 (s, 3H), 1.75 (d, 1H, *J* = 10.5 Hz), 1.92-1.98 (m, 1H), 2.10-2.16 (m, 1H), 2.32-2.39 (m, 1H), 2.44-2.53 (m, 1H), 2.54 (s, 3H), 2.72 (t, 1H, *J* = 5.4 Hz), 3.65-3.72 (m, 2H), 3.79-3.84 (m, 1H), 3.87 (d, 1H, *J* = 12.8 Hz), 4.10 (d, 1H, *J* = 12.4 Hz), 4.12 (d, 1H, *J* = 12.4 Hz), 4.30 (br d, 1H, *J* = 11.7 Hz), 7.28-7.46 (m, 5H), ^{13}C NMR (400 MHz, $CDCl_3$): δ = 23.9, 26.3, 28.2, 37.1, 38.5, 38.8, 39.5, 45.1, 57.3, 62.7, 64.1, 64.7, 128.3, 128.8, 129.9, 135.7. Anal. calcd for $C_{18}H_{27}NO_2$ (289.41): C, 74.70; H, 9.40; N, 4.84; Found: C, 74.38; H, 9.53; N, 4.65. HRMS-ESI [M+H]+*m/z* found 290.21080, calcd for $C_{18}H_{28}NO_2$: 290.21146.

(3a*S*,4*R*,6*R*,7a*S*)-3-Benzyl-3a-(hydroxymethyl)-5,5-dimethylhexahydro-4,6-methanobenzo[*d*]oxazol-2(3H)-one (19)

To a solution of **9** (0.30 g, 1.42 mmol) in DMF (10 mL), benzyl bromide (0.291 g, 0.202 mL, 1.7 mmol), Cs_2CO_3 (0.300 g, 0.92 mmol), and a catalytic amount of KI (16.0 mg) were added and the mixture was stirred for 24 h at 80 °C. After cooling and removal of the solid by filtration, the solvent was evaporated under vacuum and the residual oily product was dissolved in a mixture of DCM (20 mL) and H_2O (20 mL). The aqueous phase was extracted with DCM (3 × 20 mL) and the combined organic layer was dried (Na_2SO_4), filtered, and evaporated to dryness. The crude product was purified by column chromatography on silica gel (DCM/MeOH 19:1).

Compound **19**: 0.19 g (45 %), yellow oil, $[\alpha]_D^{20}$ = –20 (c = 0.285, MeOH), ^{1}H NMR (400 MHz, $CDCl_3$): δ = 0.80 (s, 3H), 0.87 (d, 1H, *J* = 9.1 Hz), 1.12 (s, 3H), 1.71-1.81 (m, 4H), 2.33-2.41 (m, 1H), 3.40 (dd, 1H, *J* = 5.7, 12.1 Hz), 3.47 (dd, 1H, *J* = 5.6, 12.2 Hz), 4.08 (d, 1H, *J* = 15.7 Hz), 4.38 (d, 1H, *J* = 15.7 Hz), 4.73 (d, 1H, *J* = 8.3 Hz), 5.12 (t, 1H, *J* = 5.1 Hz), 7.20-7.38 (m, 5H), ^{13}C NMR (400 MHz, $CDCl_3$): δ = 22.5, 27.2, 27.4, 35.1, 37.9, 39.2, 43.9, 45.4, 62.3, 69.7, 70.5, 127.5, 128.5, 128.8, 138.8, 158.2. Anal. calcd for $C_{18}H_{23}NO_3$ (301.38): C, 71.73; H, 7.69;

N, 4.65, Found: C, 71.89; H, 7.48; N, 4.81. HRMS-ESI [M+H]+*m/z* found 302. 17551, calcd for $C_{18}H_{24}NO_3$: 302.17562.

1-((1*R*,2*S*,3*S*,5*R*)-3-Hydroxy-2-hydroxymethyl-6,6-dimethylbicyclo[3.1.1]heptan-2-yl)-3-phenylthiourea (20)

To a solution of aminodiol **13** (84 mg, 0.377 mmol) in toluene (30 mL), 1.05 equiv of phenyl isothiocyanate (54 mg, 47 µL, 0.396 mmol) was added and the reaction mixture was stirred for 12 h at room temperature. After evaporation, the crude product was purified by column chromatography on silica gel ($CHCl_3$/MeOH 19:1).

Compound **20**: 85 mg (70%), white powder, mp: 168–170 °C, $[\alpha]_D^{20}$ = +134 (c = 0.275, MeOH), ^{1}H NMR (500 MHz, $CDCl_3$) δ = 1.02 (s, 3 H), 1.10-1.12 (d, 1H, *J* = 10.8 Hz), 1.29 (s, 3 H), 1.49-1.53 (m, 1H), 1.62 (s, 2H), 1.93-1.94 (m, 1H), 2.28-2.32 (m, 1H), 2.39-2.43 (m, 1H), 2.65 (s, 1H), 3.27 (t, 1H, *J* = 5.8 Hz), 3.68 (d, 1H, *J* = 11.1 Hz), 4.56 (t, 1H, *J* = 8.2 Hz), 4.92 (d, 1H, *J* = 10.9 Hz), 7.23-7.29 (m, 2H), 7.40-7.43 (m, 3H), 7.60 (s, 1H); ^{13}C NMR (125 MHz, $CDCl_3$) δ = 24.5, 28.6, 29.3, 36.2, 38.5, 40.2, 47.2, 66.0, 66.9, 68.4, 125.1, 127.3, 130.1, 136.4, 178.6. Anal. calcd for $C_{17}H_{24}N_2O_2S$ (320.45): C, 63.72; H, 7.55; N, 8.74; Found: C, 63.75; H, 7.53; N, 8.71. HRMS-ESI [M+H]+*m/z* found 321. 16303, calcd for $C_{17}H_{25}N_2O_2S$: 321.16367.

(1*R*,2*S*,3*S*,5*R*)-6,6-Dimethyl-2'-(phenylimino)spiro[bicyclo[3.1.1]heptan-2,4'-oxazolidine]-3-ol (21A)

Thiourea **20** (56 mg, 0.174 mmol) was dissolved and stirred in a solution of methanol (5 mL) and MeI (5.5 equiv, 60 µL, 0.96 mmol) at room temperature. After stirring for 3 h the solvent was evaporated and the residue was redissolved and stirred in 2 mL of 2.5 N methanolic potassium hydroxide for 12 h at room temperature. After evaporation of the solvent, the residue was dissolved in a mixture of H_2O (10 mL) and $CHCl_3$ (10 mL) and the aqueous phase was extracted with $CHCl_3$ (2 × 10 mL) and the organic layer was dried (Na_2SO_4), and evaporated. The crude product was then purified by column chromatography on silica gel (*n*-hexane/EtOAc 1:1).

Compound **21A**: 33 mg (66%), orange solid, mp: 138–140 °C, $[\alpha]_D^{20}$ = +13 (c = 0.335, MeOH), ^{1}H NMR (500 MHz, $CDCl_3$) δ = 0.85 (s, 3H), 1.15-1.29 (m, 1H), 1.26 (m, 3H), 1.73-1.75 (m, 1H), 1.91-1.95 (m, 1H), 1.96-1.99 (m, 1H), 2.02-2.05 (m, 1H), 2.22-2.27 (m, 1H), 2.39-2.44 (m, 1H), 3.93-3.96 (m, 1H), 4.16 (s, 2H), 6.99-7.02 (m, 1H), 7.26-7.29 (m, 3H), 7.40-7.41 (m, 2H); ^{13}C NMR (125 MHz, $CDCl_3$) δ = 23.4, 26.9, 27.6, 29.7, 37.5, 38.4, 40.3, 53.1, 70.1, 80.0, 118.6, 123.0, 129.1, 139.1, 157.0. Anal. calcd for $C_{17}H_{22}N_2O_2$ (286.37): C, 71.30; H, 7.74; N, 9.78; Found: C, 71.33; H, 7.77; N, 9.81. HRMS-ESI [M+H]+*m/z* found 287.17545, calcd for $C_{17}H_{23}N_2O_2$: 287.17595.

((3a*S*,4*R*,6*R*,7a*S*)-5,5-dimethyl-2-(phenylimino)octahydro-4,6-methanobenzo[*d*]oxazol-3a-yl)methanol (21B)

From the $CHCl_3$ solution of **21A** (50 mg) standing for 30 days, both **21A** and **21B** tautomeric products were isolated by column chromatography on silica gel ($CHCl_3$/MeOH 19:1)

Compound **21B**: 25 mg (50%), white solid, mp: 92–95 °C, $[\alpha]_D^{20}$ = +18 (c = 0.200, MeOH), ^{1}H NMR (400 MHz, $CDCl_3$) δ = 0.88 (s, 3H), 1.20 (d, 1H, *J* = 10.8 Hz), 1.30 (s, 3H), 1.92-1.93 (m, 1H), 2.01-2.04 (m, 1H), 2.13-2.16 (m, 1H), 2.30-2.34 (m, 1H), 2.42-2.46 (m, 1H), 3.60 (d, 1H, *J* = 11.1 Hz), 3.66 (d, 1H, *J* = 11.1 Hz), 4.80 (d, 1H, *J* = 8.1 Hz), 7.03 (t, 1H, *J* = 7.1 Hz), 7.27-7.30 (t, 3H, *J* = 7.8 Hz), 7.37-7.38 (m, 2H), ^{13}C NMR (125 MHz, $CDCl_3$) δ = 24.5, 27.1, 27.3, 30.0, 34.5, 38.7, 39.3, 47.7, 68.0, 75.4, 119.1.4, 123.3, 129.4, 139.0, 156.6. Anal. calcd for $C_{17}H_{22}N_2O_2$ (286.37): C, 71.30; H, 7.74; N, 9.78; Found: C, 71.11; H, 7.67; N, 9.93. HRMS-ESI [M+H]+*m/z* found 287.17533, calcd for $C_{17}H_{23}N_2O_2$: 287.17595.

X-Ray structure determinations

The crystals of **9** and **17** were immersed in cryo-oil, mounted in a loop, and measured at a temperature of 120 K. The X-ray diffraction data were collected on a Rigaku Oxford Diffraction Supernova diffractometer using Cu Kα radiation. The CrysAlisPro [4] software package was used for cell refinements and data reductions. A multi-scan (**9**) or an analytical absorption correction (**17**) was applied to the intensities before structure solutions by using CrysAlisPro [4] software. The structures were solved by intrinsic phasing (SHELXT [5]) method. Structural refinements were carried out using SHELXL [6] software with SHELXLE [7] graphical user interface. The NH and OH hydrogen atoms were located from the difference Fourier map and refined isotropically. All other hydrogen atoms were positioned geometrically and constrained to ride on their parent atoms, with C–H = 0.95–1.00 Å and U_{iso} = 1.2–1.5 U_{eq}(parent atom). The crystallographic details are summarized in Table S1.

X-Ray structure determinations

The crystals of **9** and **17** were immersed in cryo-oil, mounted in a loop, and measured at a temperature of 120 K. The X-ray diffraction data were collected on a Rigaku Oxford Diffraction Supernova diffractometer using Cu Kα radiation. The CrysAlisPro software package was used for cell refinements and data reductions. A multi-scan (**9**) or an analytical absorption correction (**17**) was applied to the intensities before structure solutions by using CrysAlisPro software. The structures were solved by intrinsic phasing (SHELXT) method. Structural refinements were carried out using SHELXL software with SHELXLE graphical user interface. The NH and OH hydrogen atoms were located from the difference Fourier map and refined isotropically. All other hydrogen atoms were positioned geometrically and constrained to ride on their parent atoms, with C–H = 0.95–1.00 Å and U_{iso} = 1.2–1.5 U_{eq}(parent atom). The crystallographic details are summarized in Table **S1**. The deposition number CCDC 2063842 (**9**) and CCDC 2063843 (**17**) contain supplementary crystallographic data for this paper. These data can be obtained free of charge from The Cambridge Crystallographic Data Centre via http://www.ccdc.cam.ac.uk/conts/retrieving.html (or from the Cambridge Crystallographic Data Centre, 12 Union Road, Cambridge CB2 1EZ, UK; Fax: (internat.) + 44-1223-336-033; E-mail: deposit@ccdc.cam.ac.uk)

Table S1. Crystal Data.

	**9**	**17**
empirical formula	$C_{11}H_{17}NO_3$	$C_{18}H_{25}NO_2$
fw	211.25	287.39
temp (K)	120(2)	120(2)
λ(Å)	1.54184	1.54184 Å
cryst syst	Monoclinic	Monoclinic
space group	$P2_1$	$P2_1$
a (Å)	7.93530(6)	a = 8.41050(10)
b (Å)	10.89885(6)	b = 7.23460(10)
c (Å)	12.63719(8)	c = 13.4036(2)
β (deg)	97.9249(7)	β= 102.960(2)°
V (Å^3)	1082.498(12)	794.79(2)
Z	4	2
ρ_{calc} (Mg/m^3)	1.296	1.201
μ(Mo Kα) (mm^{-1})	0.770	0.608
No. reflns.	24009	16590
Unique reflns.	4528	3329
GOOF (F^2)	1.042	1.038
R_{int}	0.0194	0.0310
R1[a] ($I \geq 2\sigma$)	0.0249	0.0305
wR2[b] ($I \geq 2\sigma$)	0.0664	0.0766

[a] $R1 = \Sigma||F_o| - |F_c||/\Sigma|F_o|$. [b] $wR2 = [\Sigma[w(F_o^2 - F_c^2)^2]/\Sigma[w(F_o^2)^2]]^{1/2}$.

(1) Szakonyi, Z.; Hetényi, A.; Fülöp, F. *Tetrahedron* **2008**, *64* (6), 1034–1039. doi:10.1016/j.tet.2007.07.065

(2) Kamon, T.; Shigeoka, D.; Tanaka, T.; Yoshimitsu, T. *Org. Biomol. Chem.* **2012**, *10* (12), 2363–2365. doi:10.1039/c2ob07190h

(3) Tashenov, Y.; Daniels, M.; Robeyns, K.; Van Meervelt, L.; Dehaen, W.; Suleimen, Y.; Szakonyi, Z. *Molecules* **2018**, *23* (4), 771. doi:10.3390/molecules23040771

(4) Rikagu Oxford Diffraction, *CrysAlisPro*, Agilent Technologies inc., 2015, Yarnton, Oxfordshire, England

(5) Sheldrick, G. M. *Acta Crystallogr. A* **2015**, *71* (1), 3–8. doi:10.1107/S2053273314026370

(6) Sheldrick, G. M. *Acta Crystallogr. C* **2015**, *71* (1), 3–8. doi:10.1107/S2053229614024218

(7) Hübschle, C. B.; Sheldrick, G. M.; Dittrich, B. *J. Appl. Crystallogr.* **2011**, *44* (6), 1281–1284. doi:10.1107/S0021889811043202

^{1}H, ^{13}C NMR, COSY, HSQC and HMBC of **8** ($CDCl_3$)

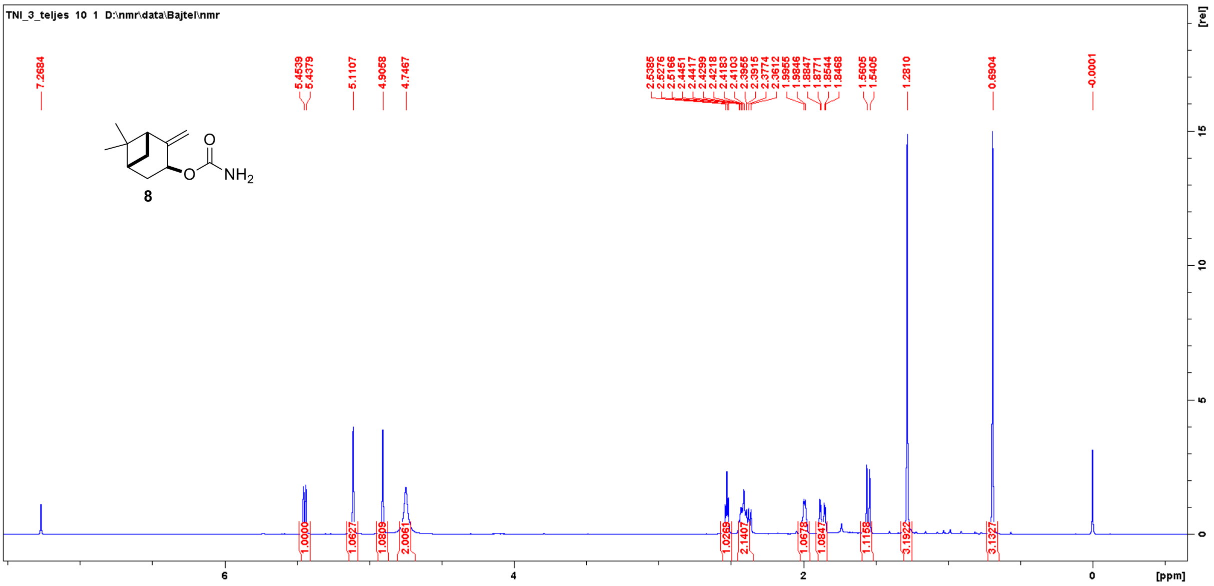

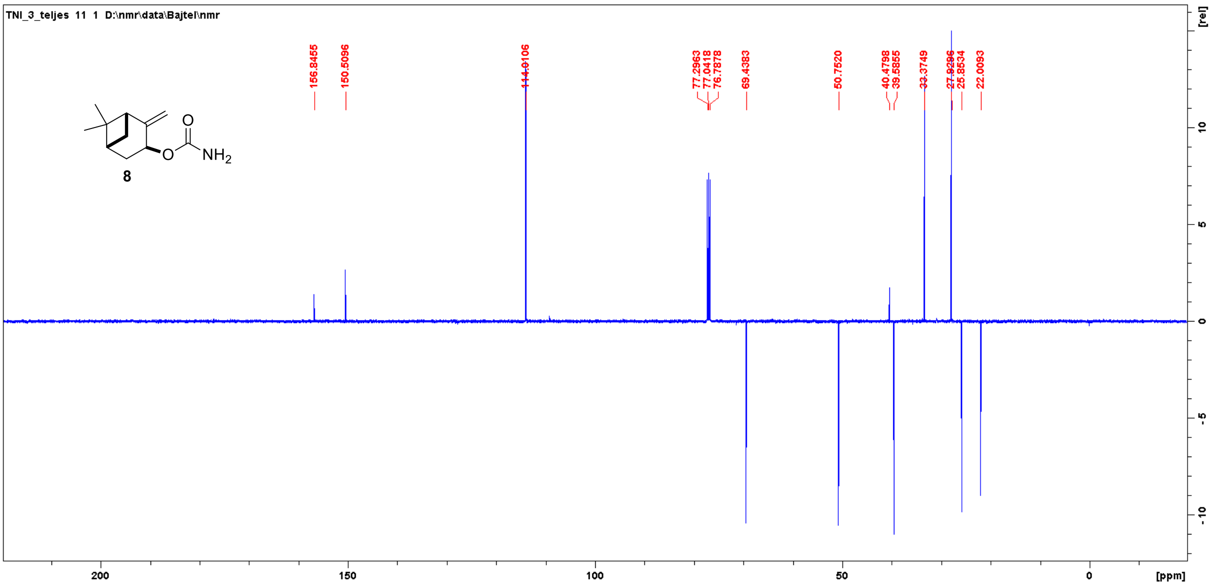

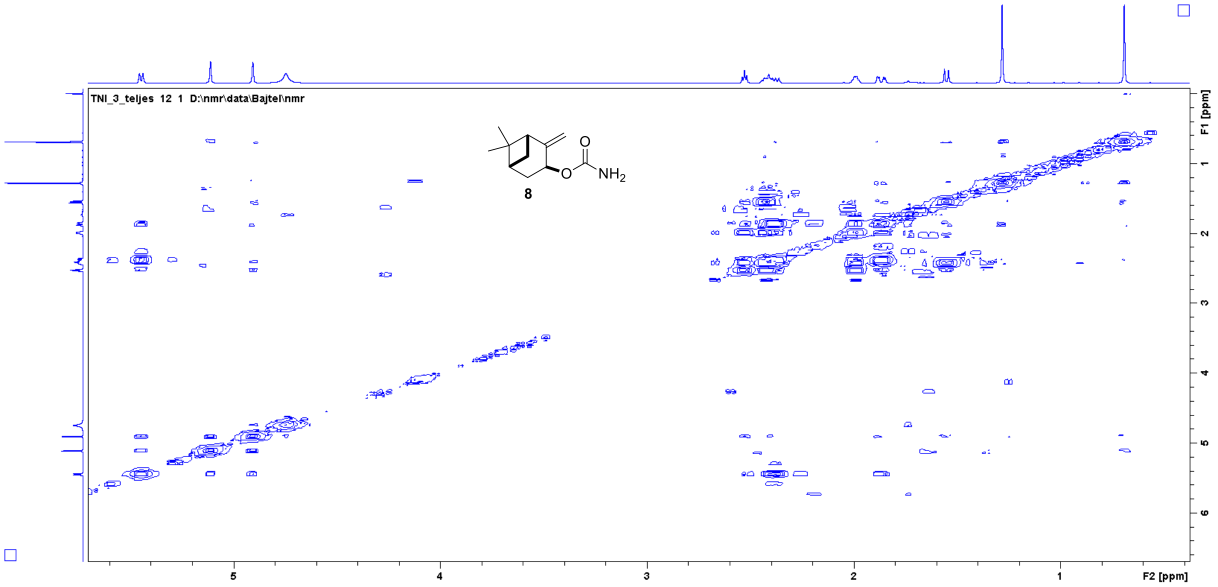

TNI_3_teljes 12 1 D:\nmr\data\Bajtel\nmr
O
NH2
8
F1 [ppm]
1
2
3
4
5
6
5
4
3
2
1
F2 [ppm]

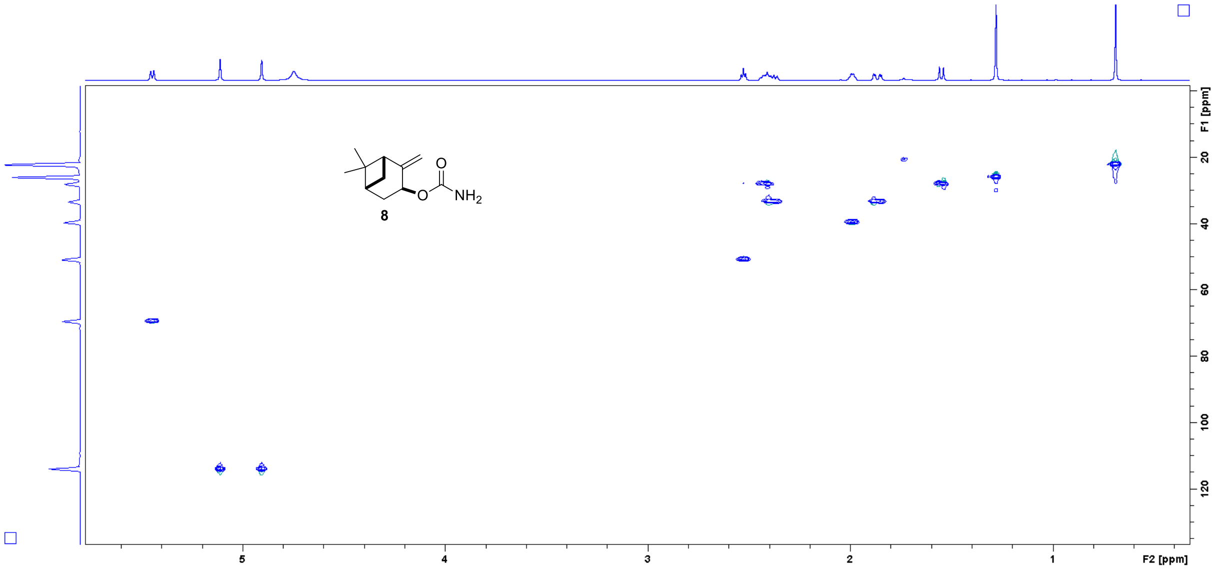

O
NH2
8
F1 [ppm]
20
40
60
80
100
120
5
4
3
2
1
F2 [ppm]

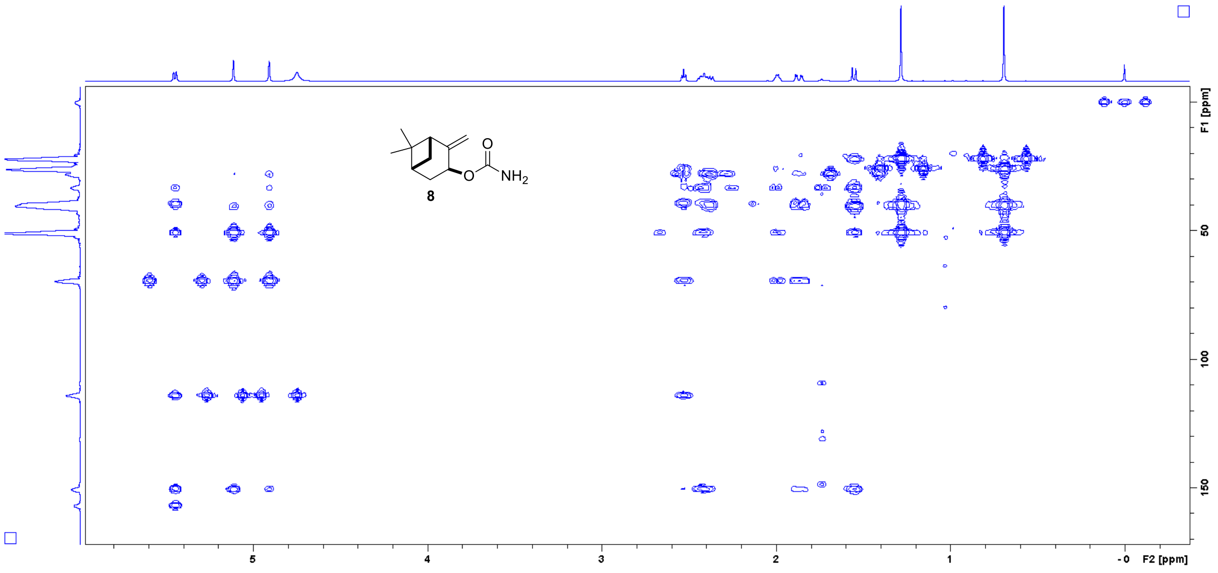

O
NH2
8
F1 [ppm]
50
100
150
5
4
3
2
1
-0
F2 [ppm]

^{1}H, ^{13}C NMR, COSY, NOESY, HSQC and HMBC of **9** (DMSO-d_6)

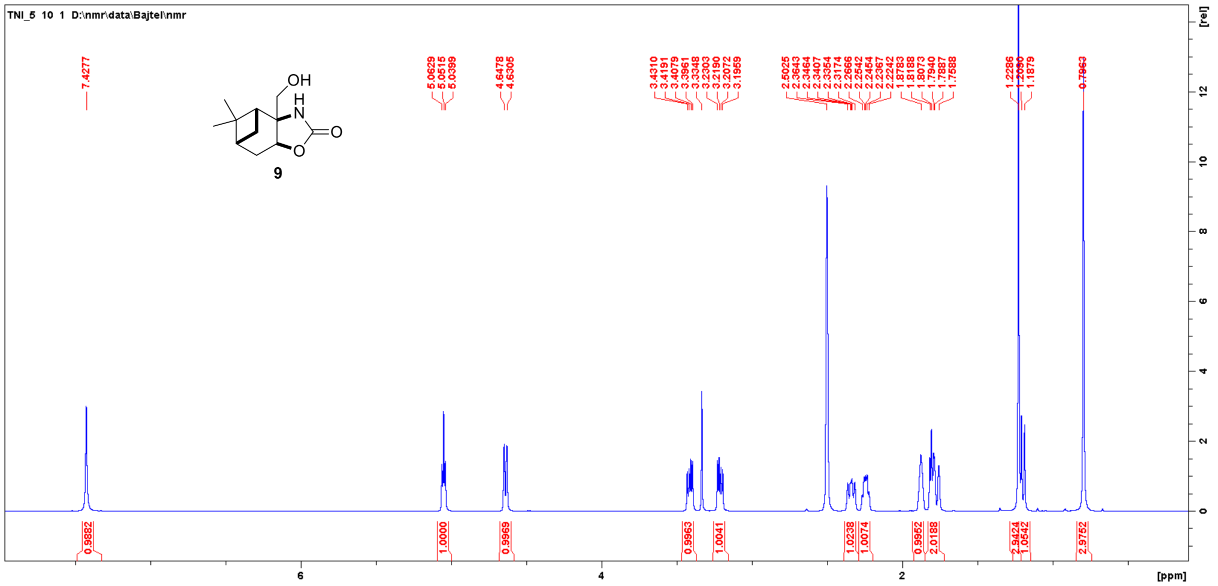

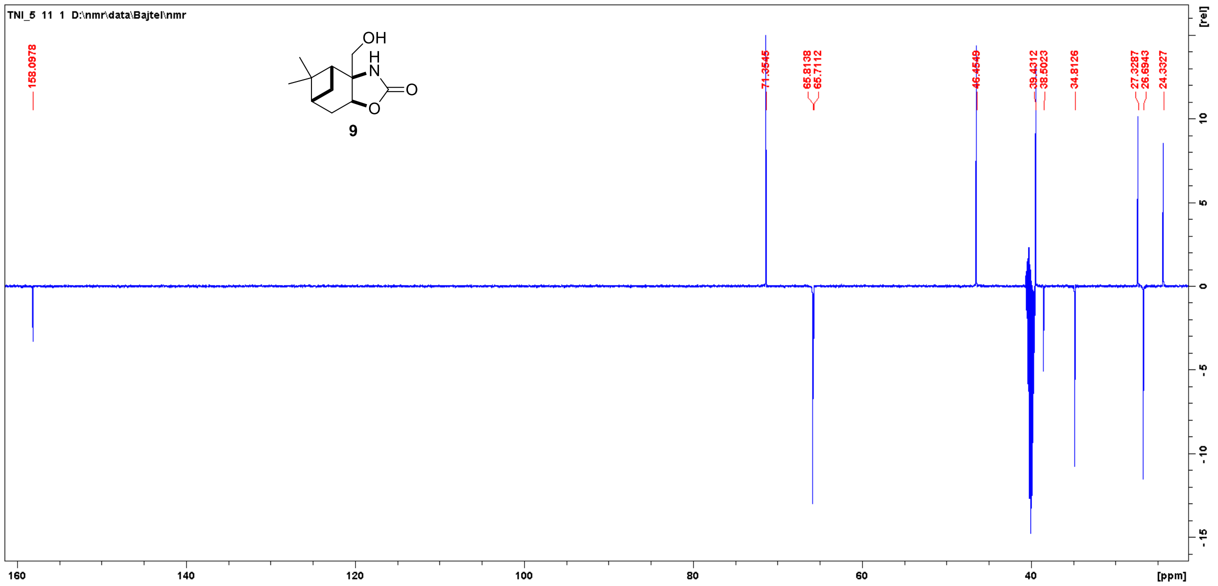

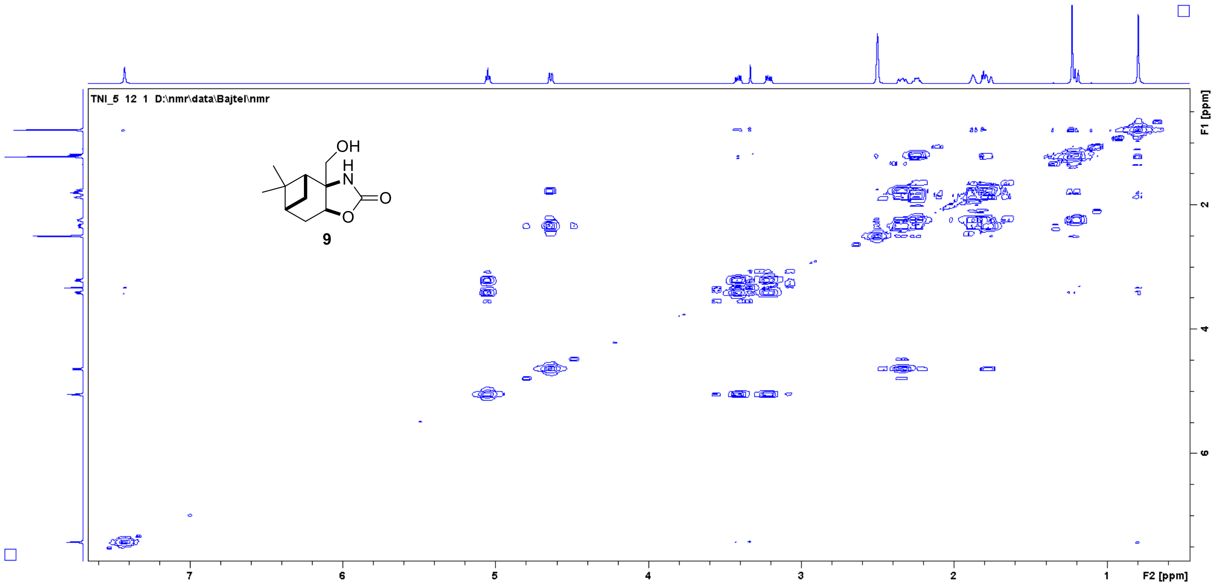

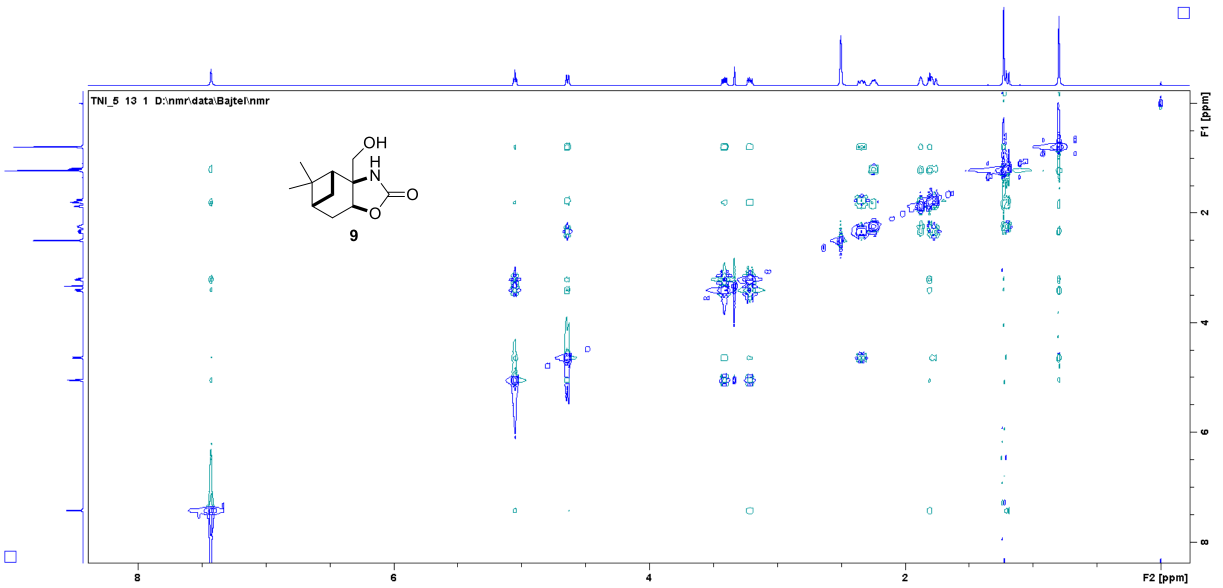
TNI_5 13 1 D:\nmr\data\Bajtel\nmr
OH
H
N
O
O
9
F1 [ppm]
F2 [ppm]

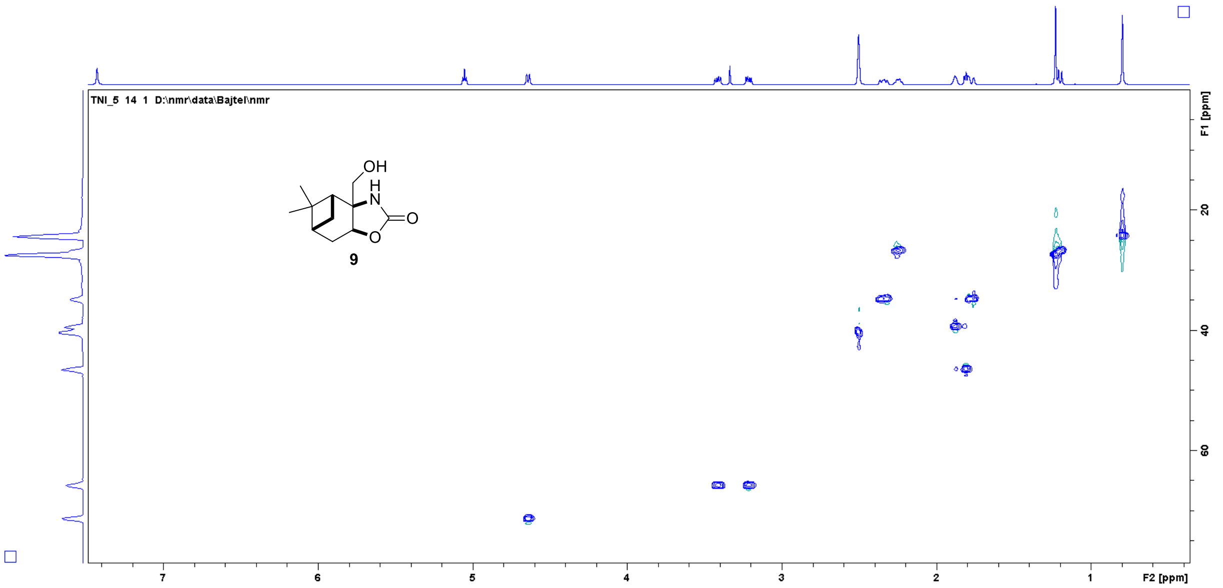
TNI_5 14 1 D:\nmr\data\Bajtel\nmr
OH
H
N
O
O
9
F1 [ppm]
F2 [ppm]

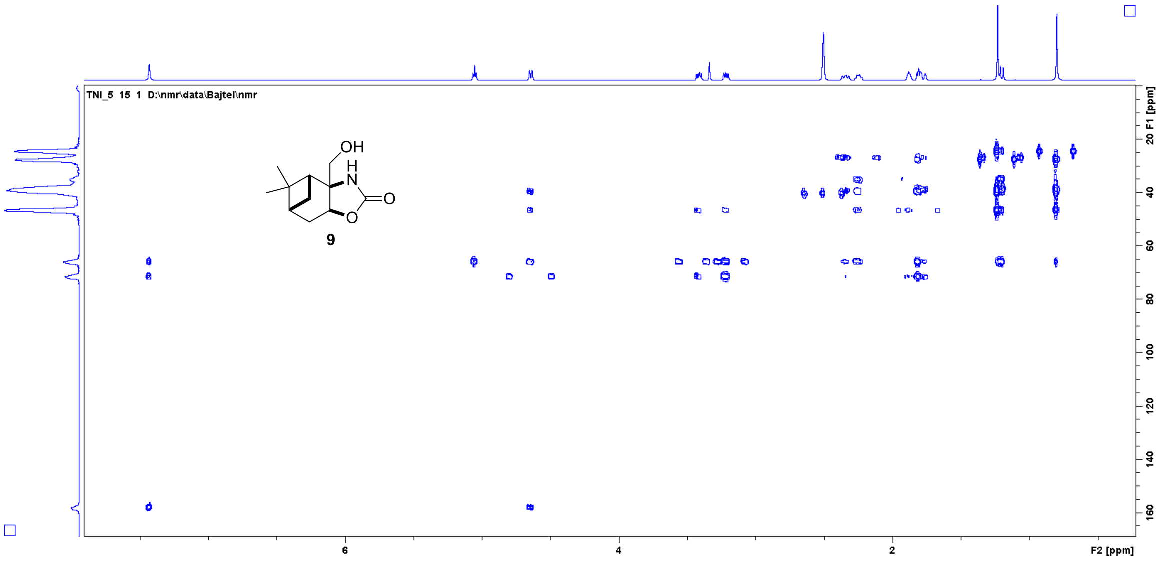
TNI_5 15 1 D:\nmr\data\Bajtel\nmr
OH
H
N
O
O
9
F1 [ppm]
F2 [ppm]

^{1}H, ^{13}C NMR, COSY, NOESY, HSQC and HMBC of **12** (DMSO-d_6)

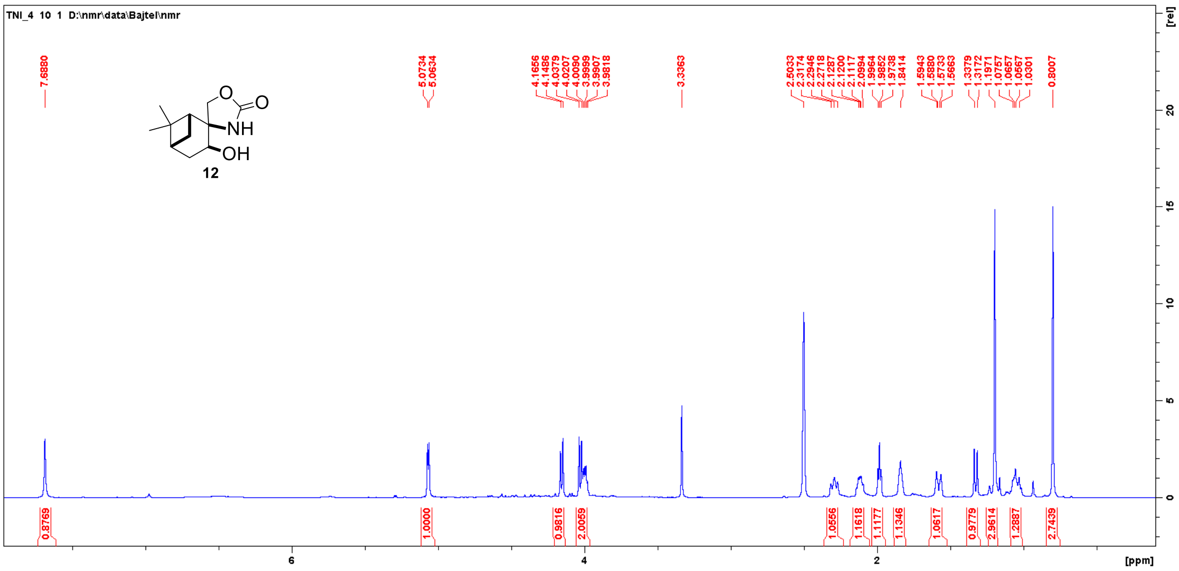

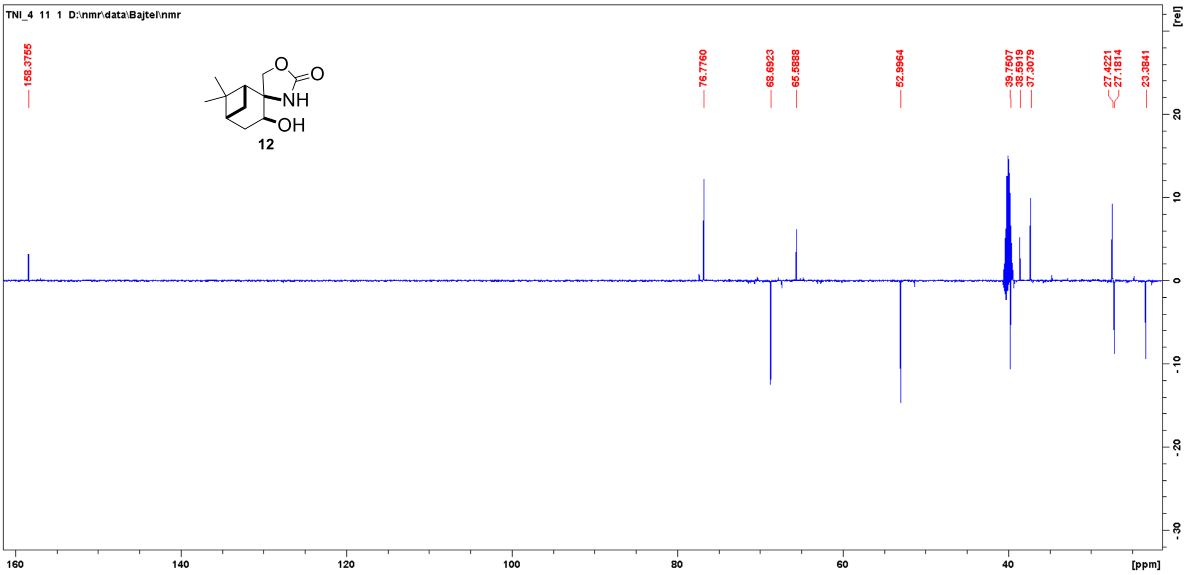

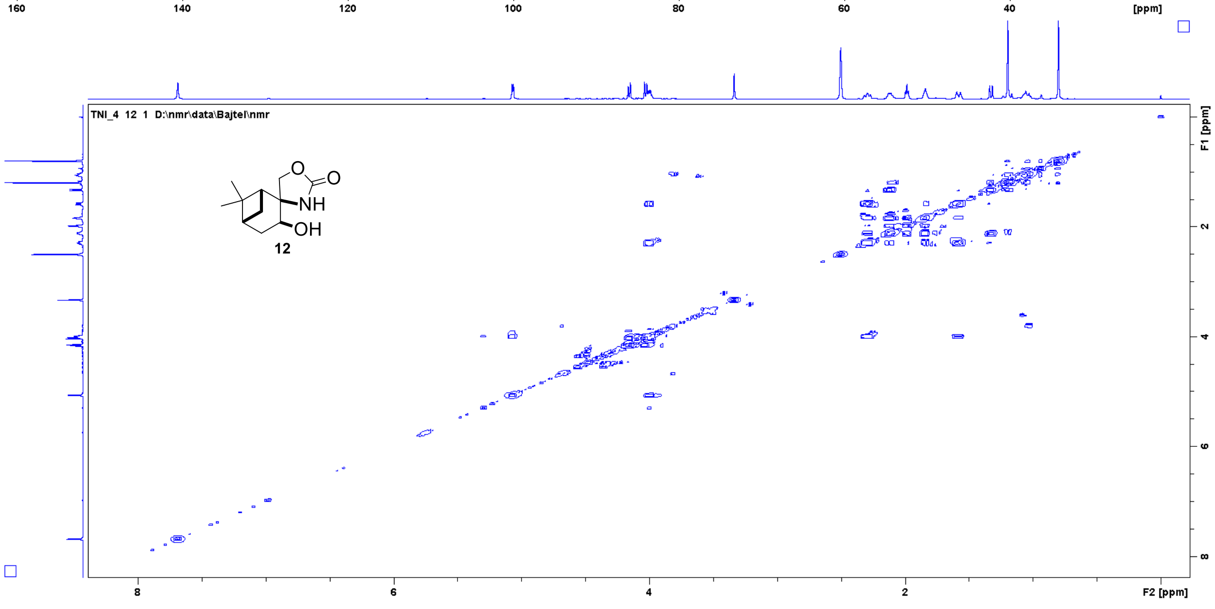

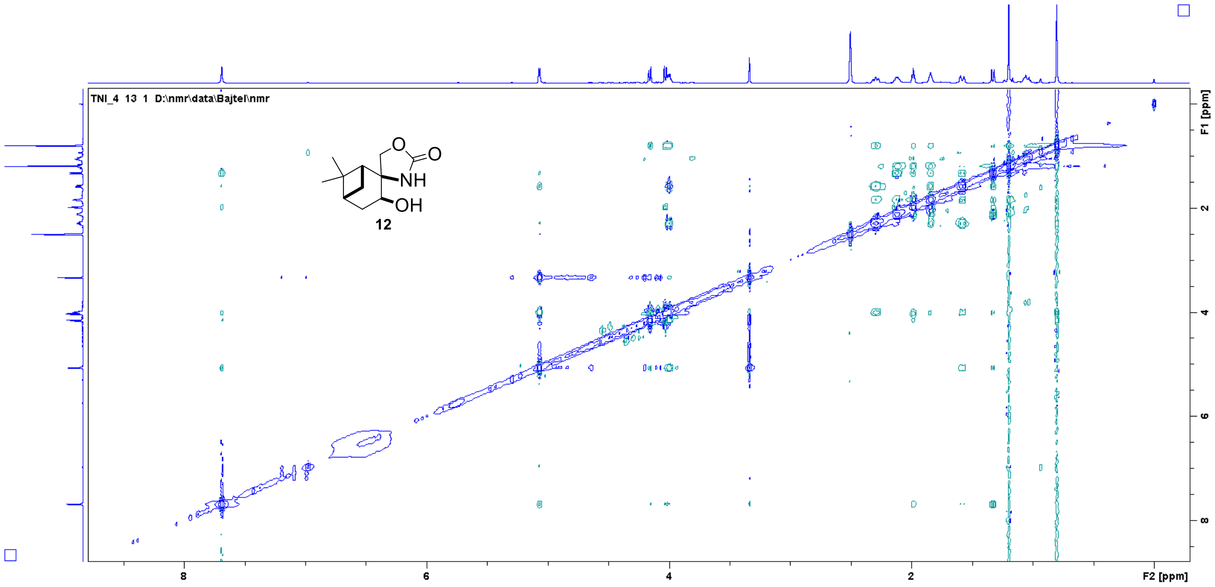
TNI_4 13 1 D:\nmr\data\Bajtel\nmr
O
O
NH
OH
12
F1 [ppm]
F2 [ppm]

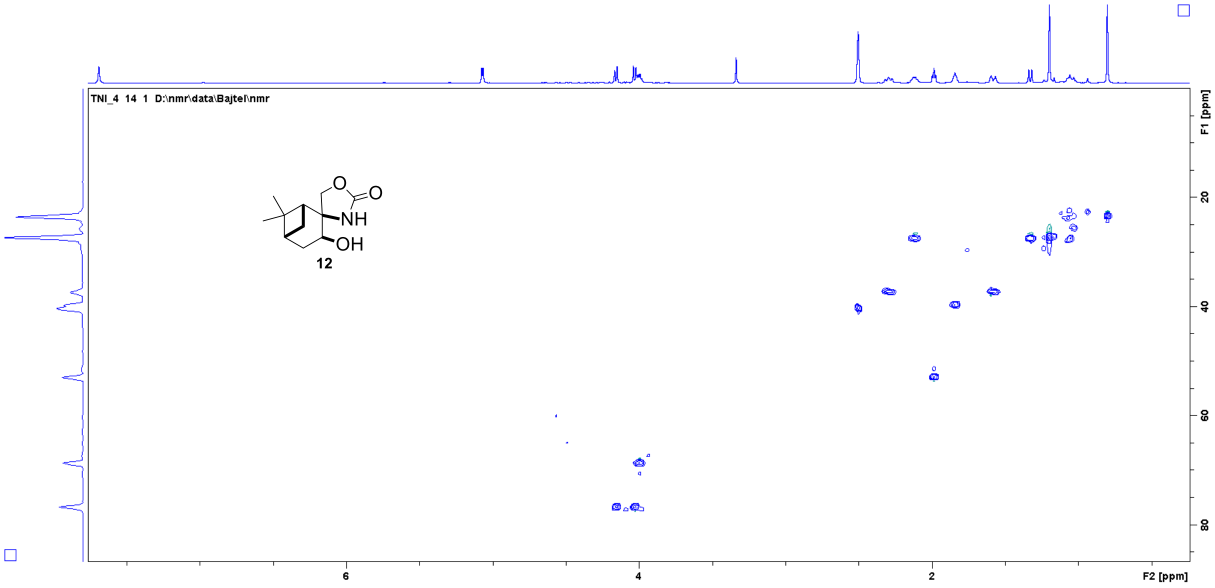
TNI_4 14 1 D:\nmr\data\Bajtel\nmr
O
O
NH
OH
12
F1 [ppm]
F2 [ppm]

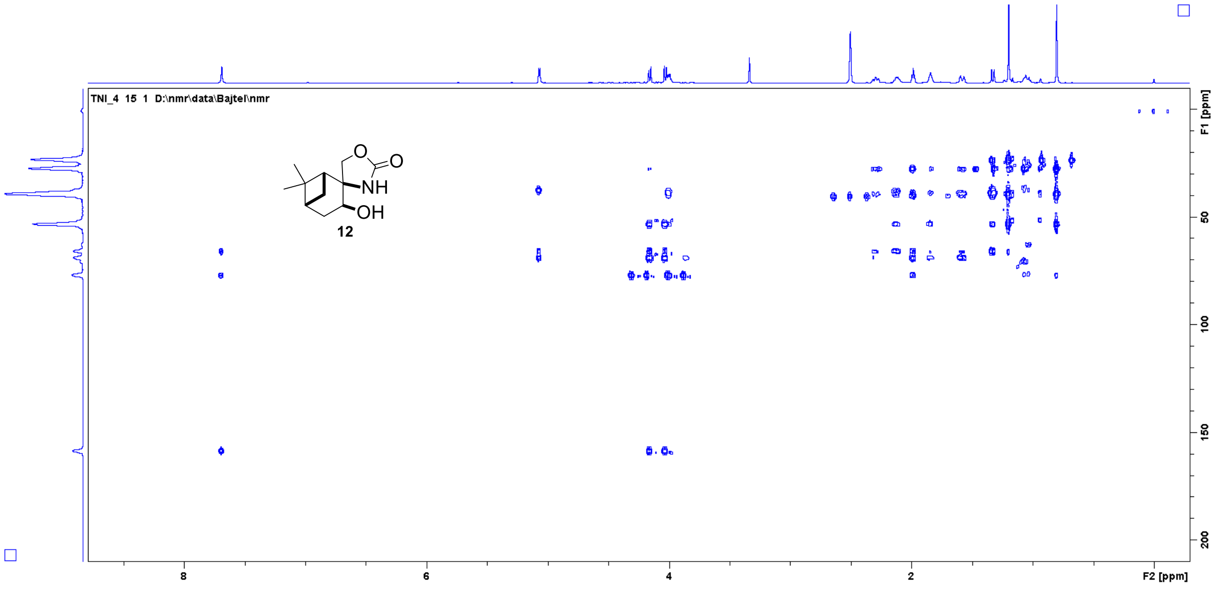
TNI_4 15 1 D:\nmr\data\Bajtel\nmr
O
O
NH
OH
12
F1 [ppm]
F2 [ppm]

^{1}H, ^{13}C NMR, COSY, NOESY, HSQC and HMBC of **13** (DMSO-d_6)

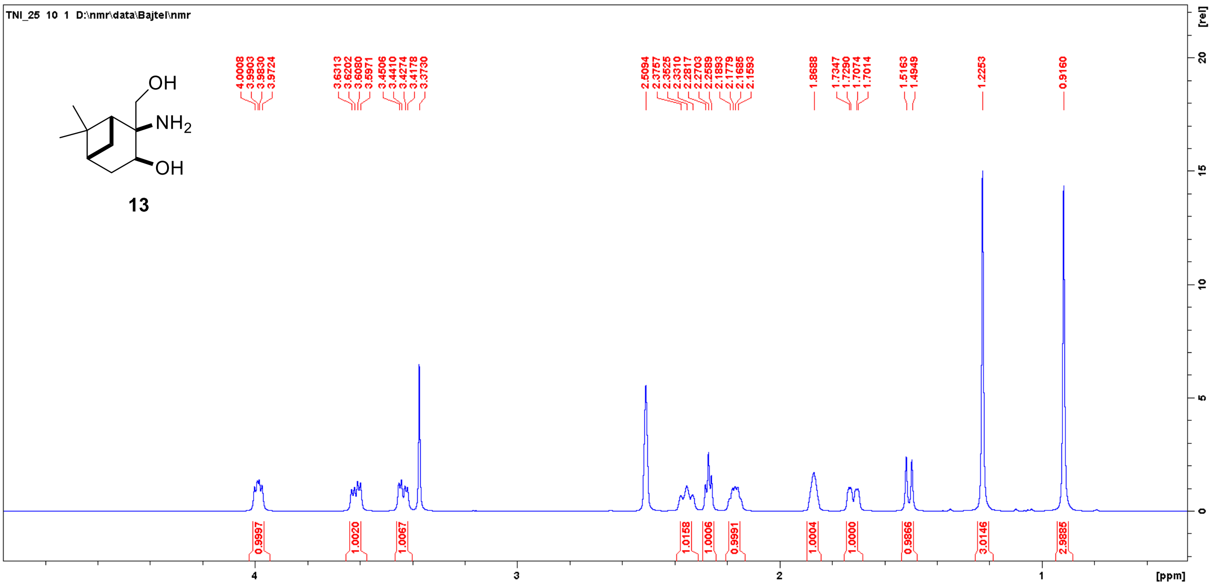

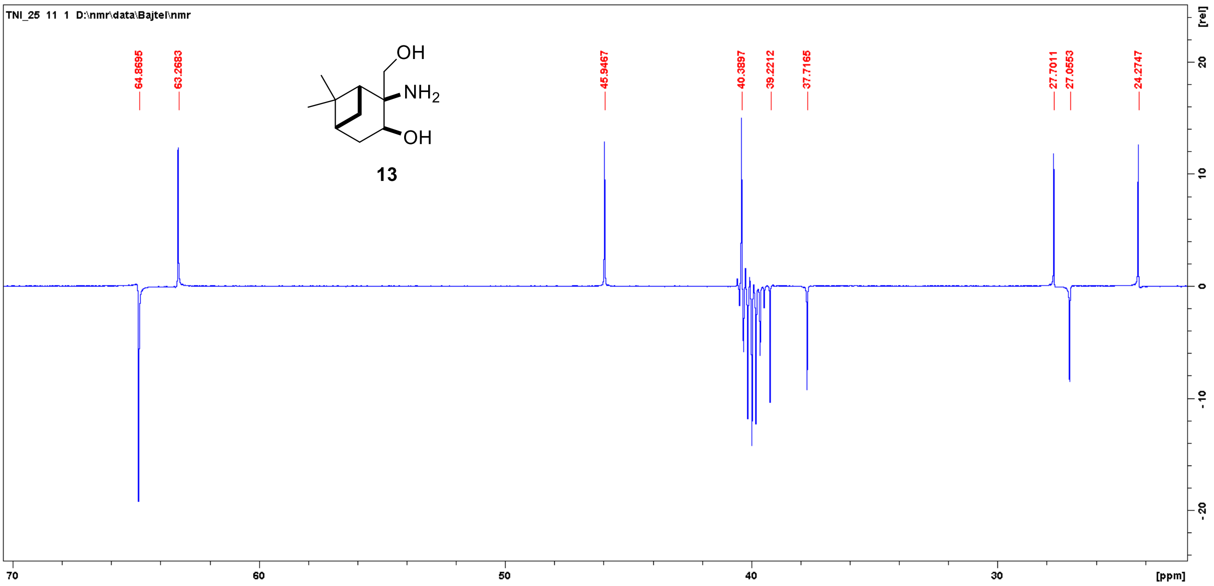

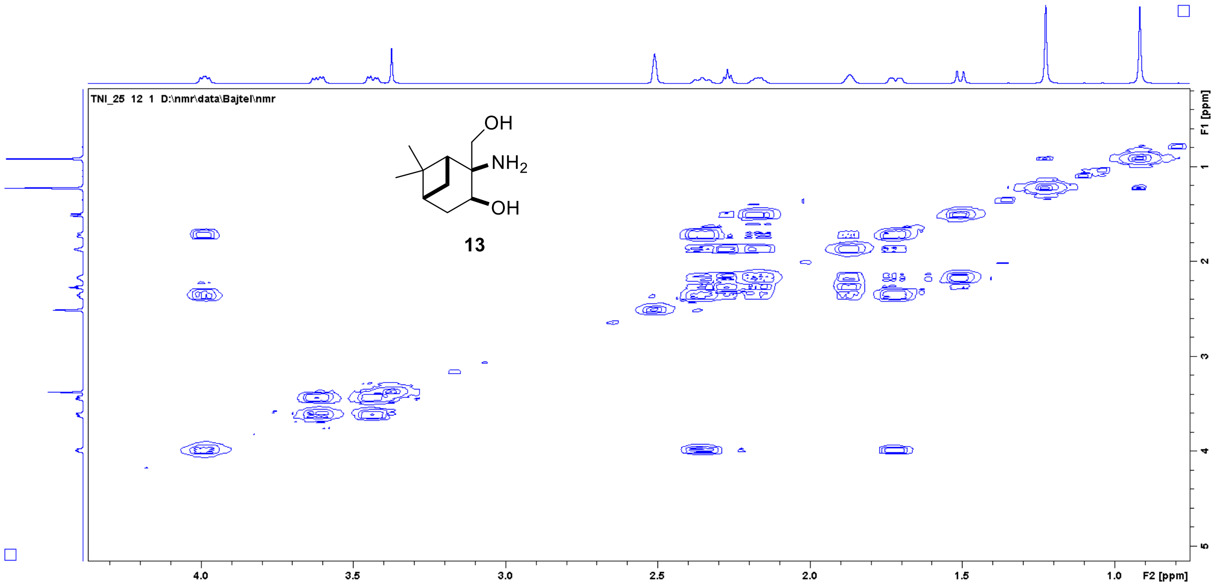

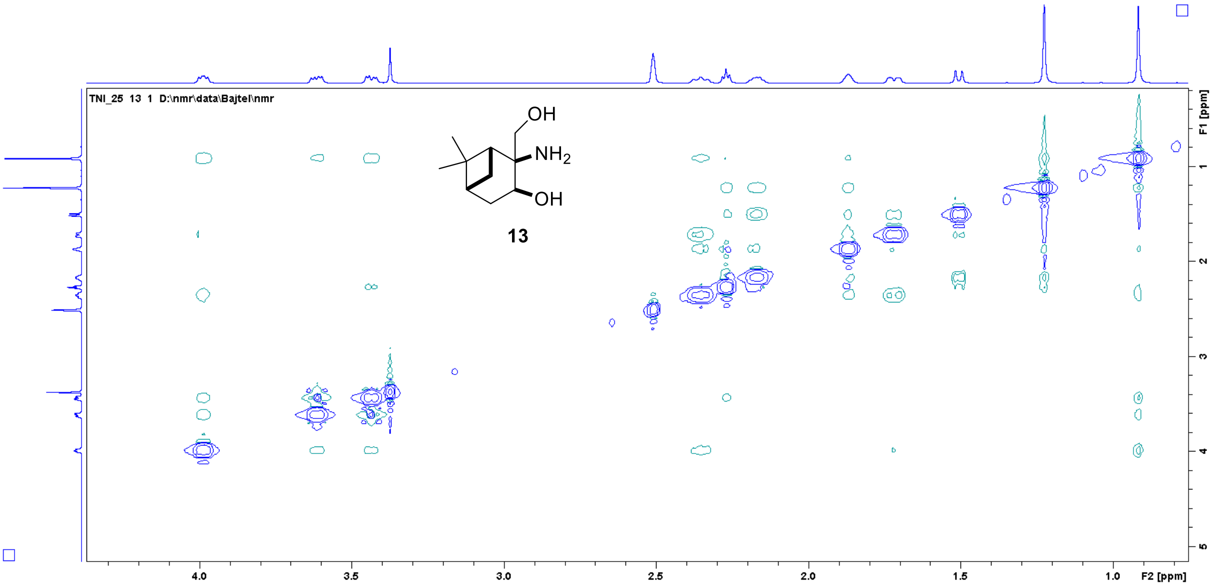
TNI_25 13 1 D:\nmr\data\Bajtel\nmr
OH
NH2
OH
13
F1 [ppm]
F2 [ppm]

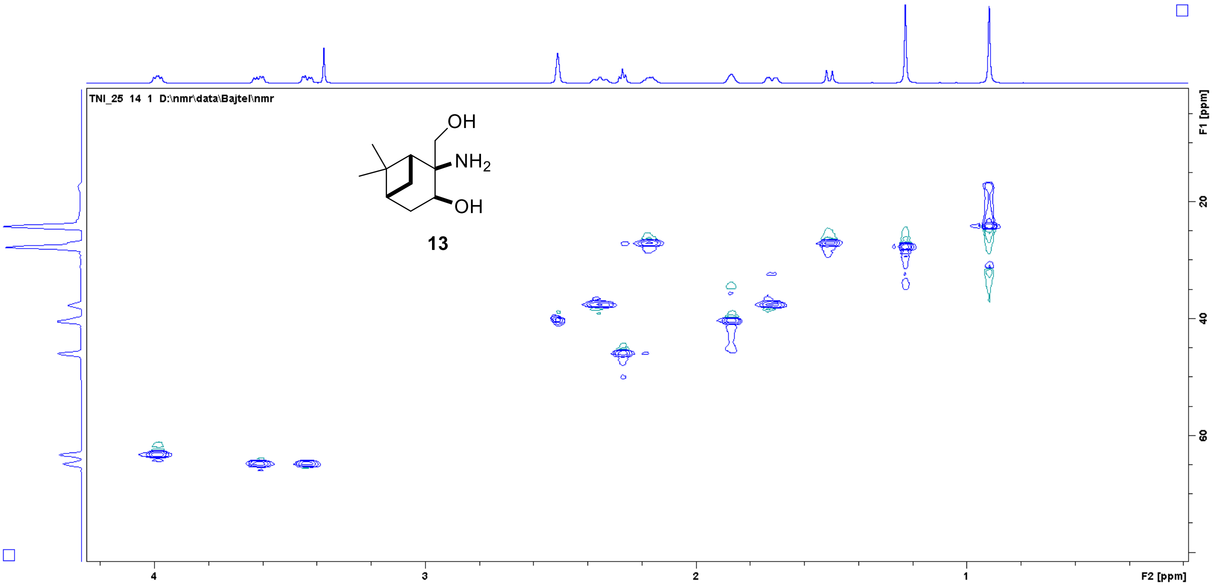
TNI_25 14 1 D:\nmr\data\Bajtel\nmr
OH
NH2
OH
13
F1 [ppm]
F2 [ppm]

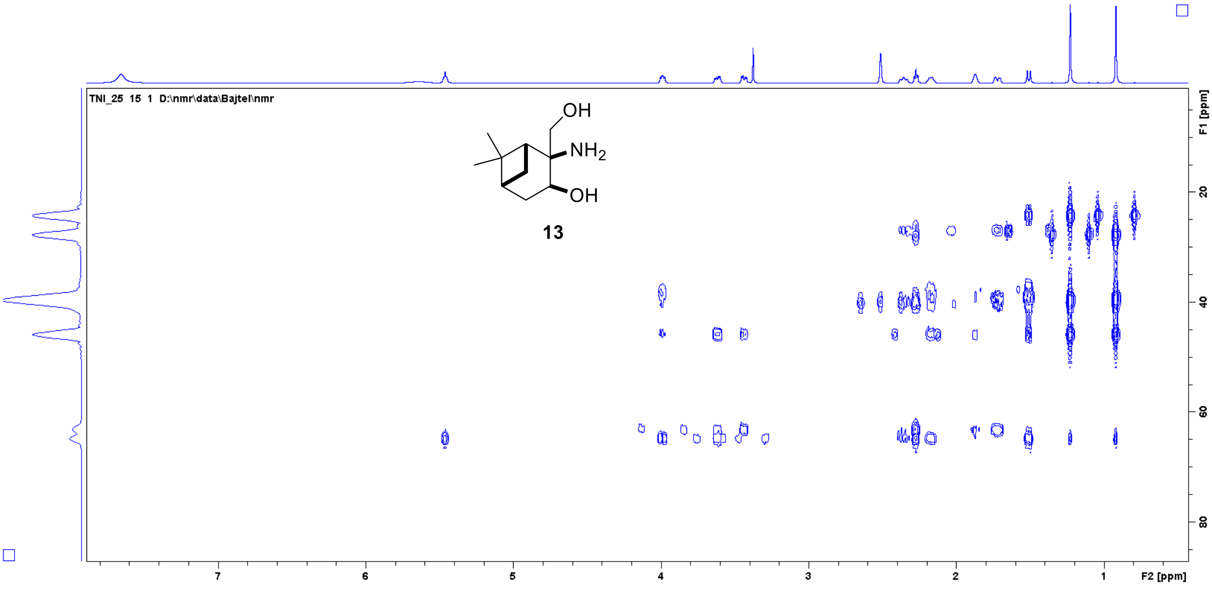
TNI_25 15 1 D:\nmr\data\Bajtel\nmr
OH
NH2
OH
13
F1 [ppm]
F2 [ppm]

^{1}H, ^{13}C NMR, COSY, HSQC and HMBC of **14** (DMSO-d_6)

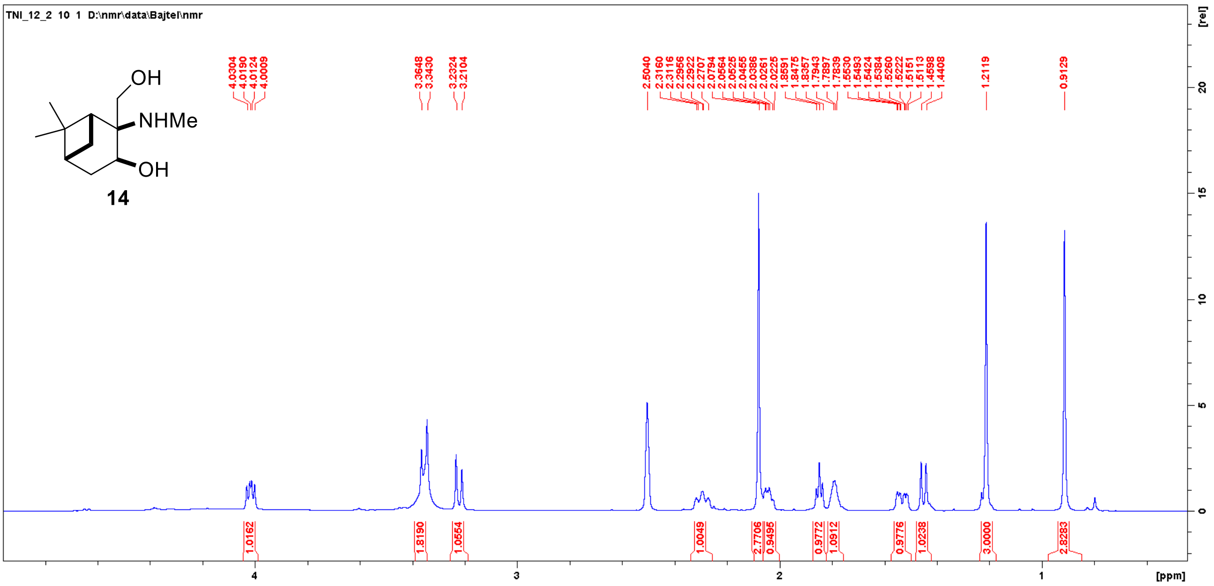

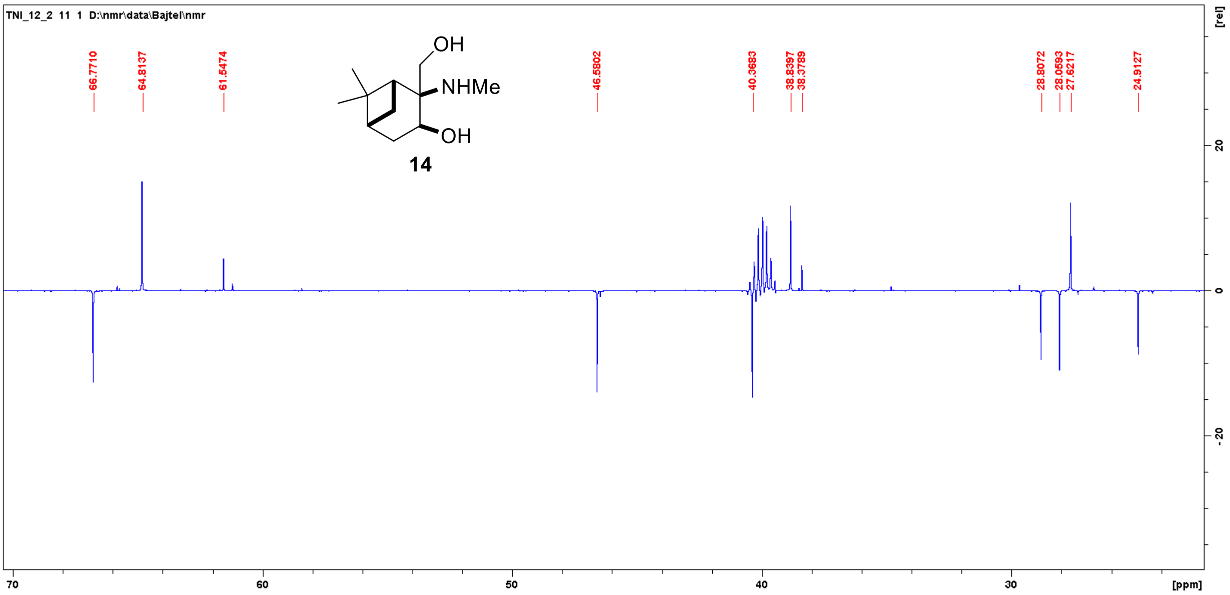

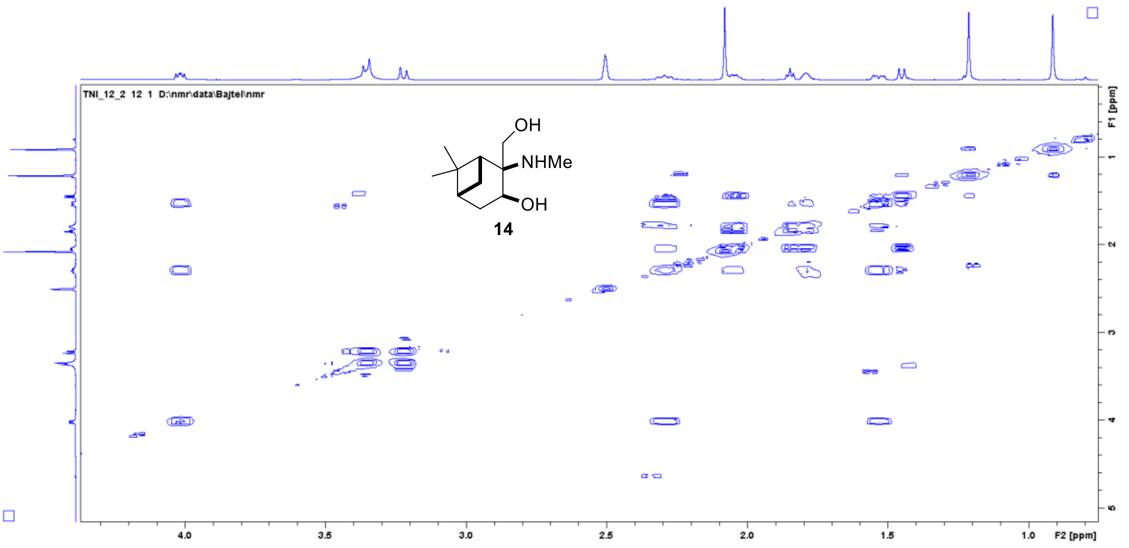

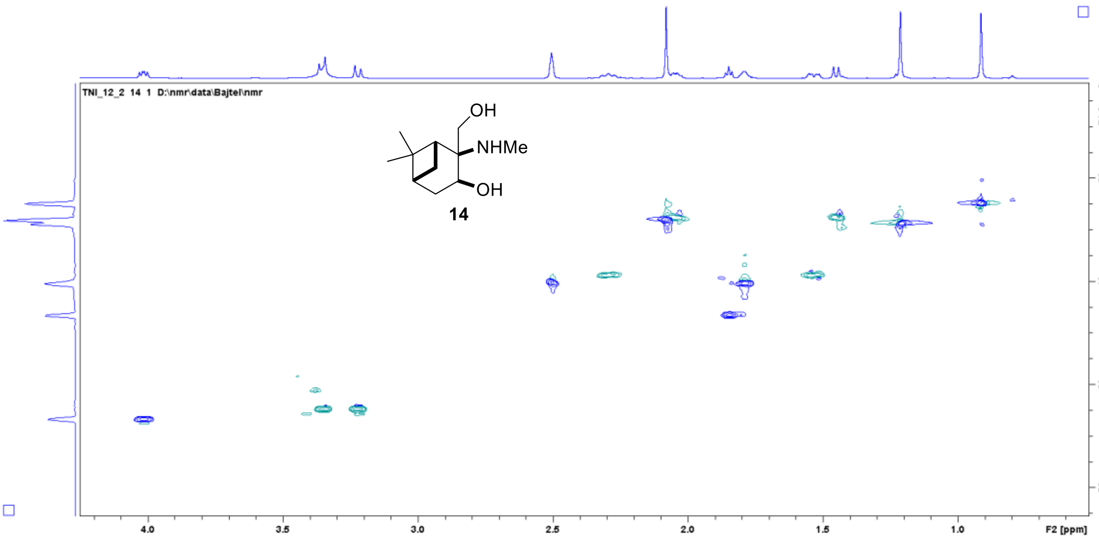
TNI_12_2 14 1 D:\nmr\data\Bajtel\nmr
OH
NHMe
OH
14
F1 [ppm]
20
40
60
80
4.0
3.5
3.0
2.5
2.0
1.5
1.0
F2 [ppm]

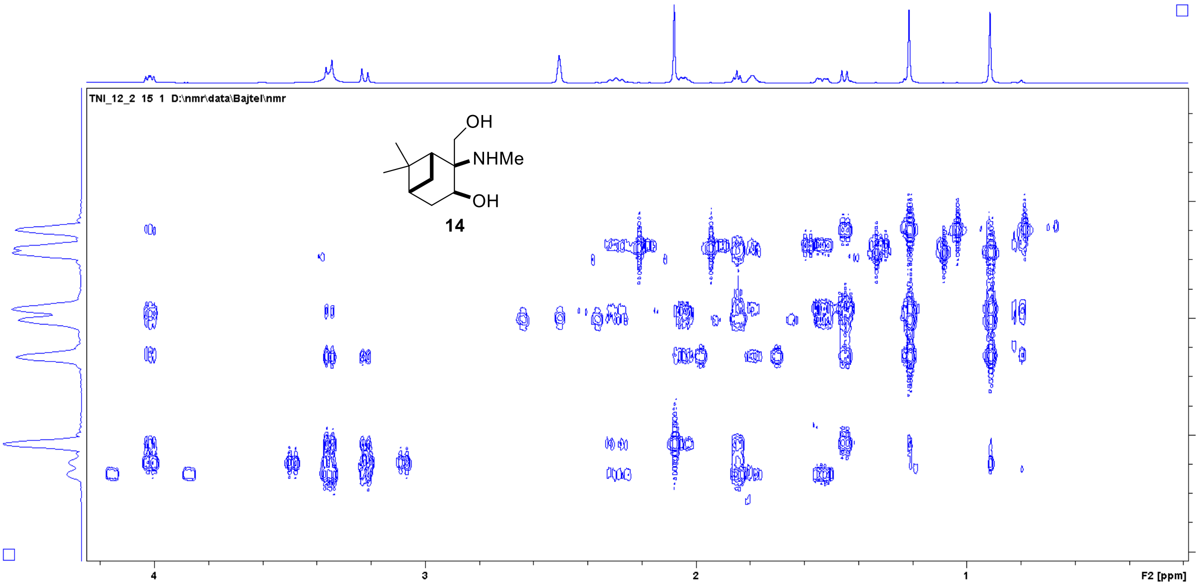
TNI_12_2 15 1 D:\nmr\data\Bajtel\nmr
OH
NHMe
OH
14
F1 [ppm]
20
40
60
4
3
2
1
F2 [ppm]

^{1}H, ^{13}C NMR, COSY, HSQC and HMBC of **16** (DMSO-d_6)

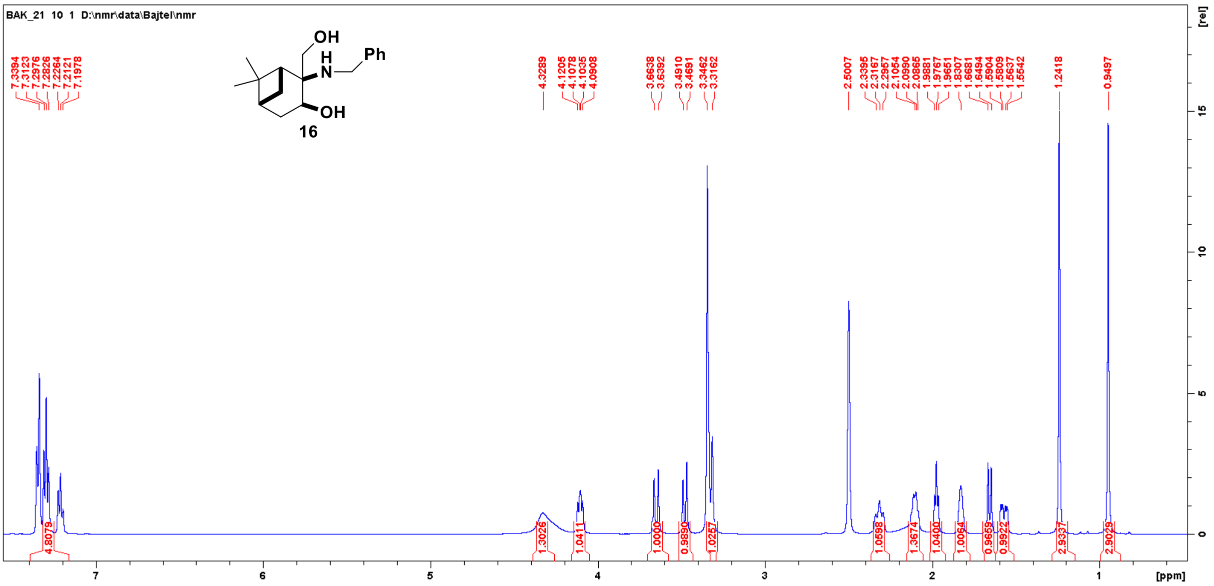

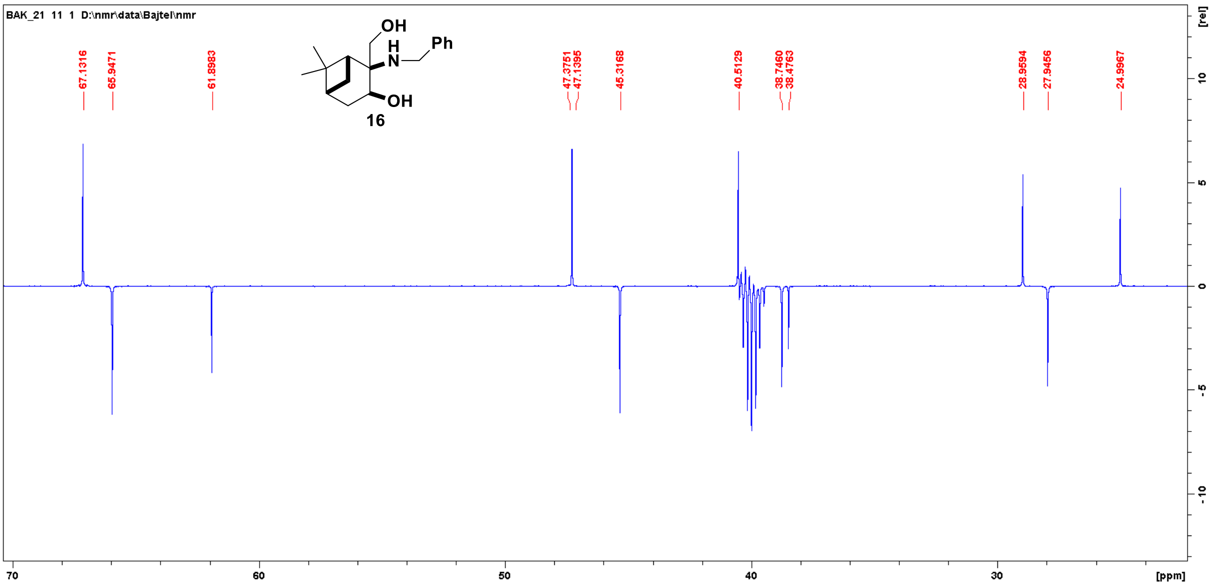

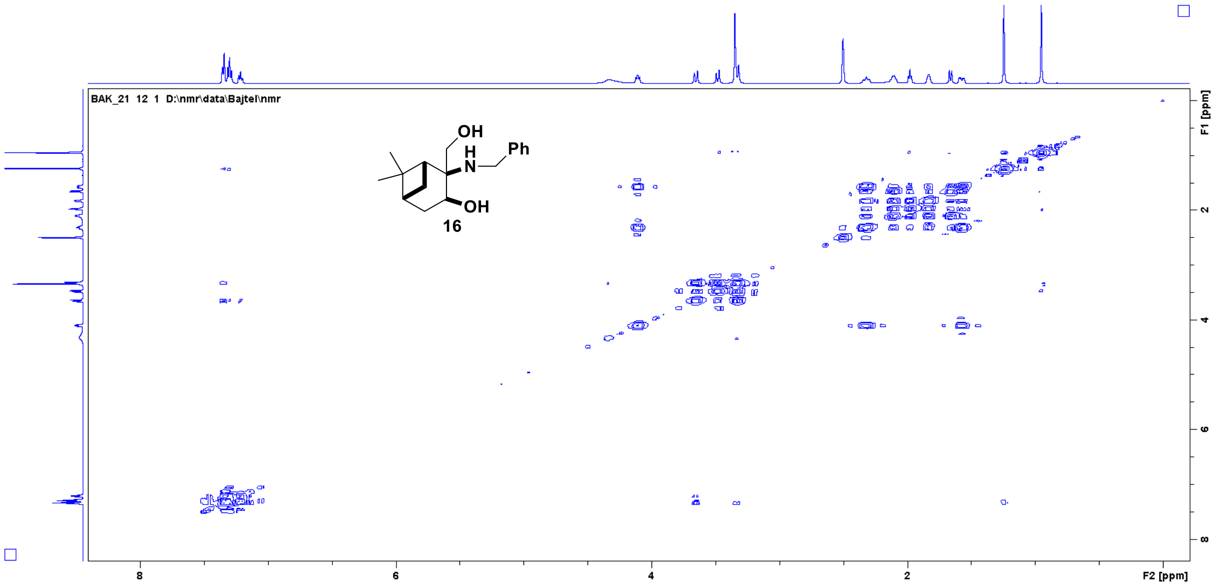

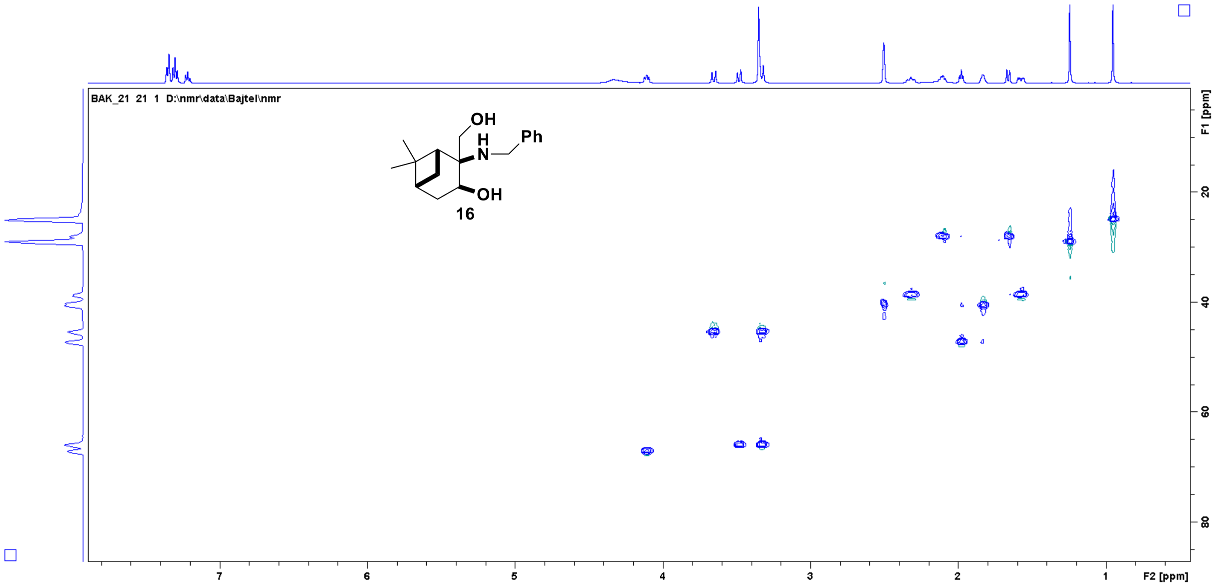
BAK_21 21 1 D:\nmr\data\Bajtel\nmr
OH
H
N
Ph
OH
16
F1 [ppm]
F2 [ppm]

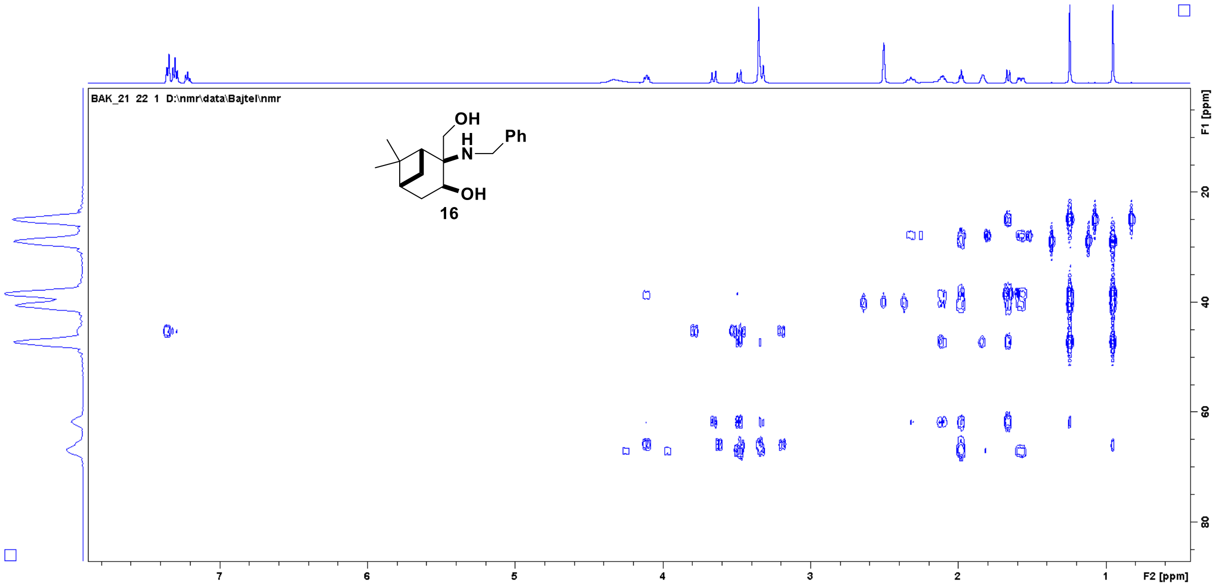
BAK_21 22 1 D:\nmr\data\Bajtel\nmr
OH
H
N
Ph
OH
16
F1 [ppm]
F2 [ppm]

^{1}H, ^{13}C NMR and HMBC of **17** ($CDCl_3$)

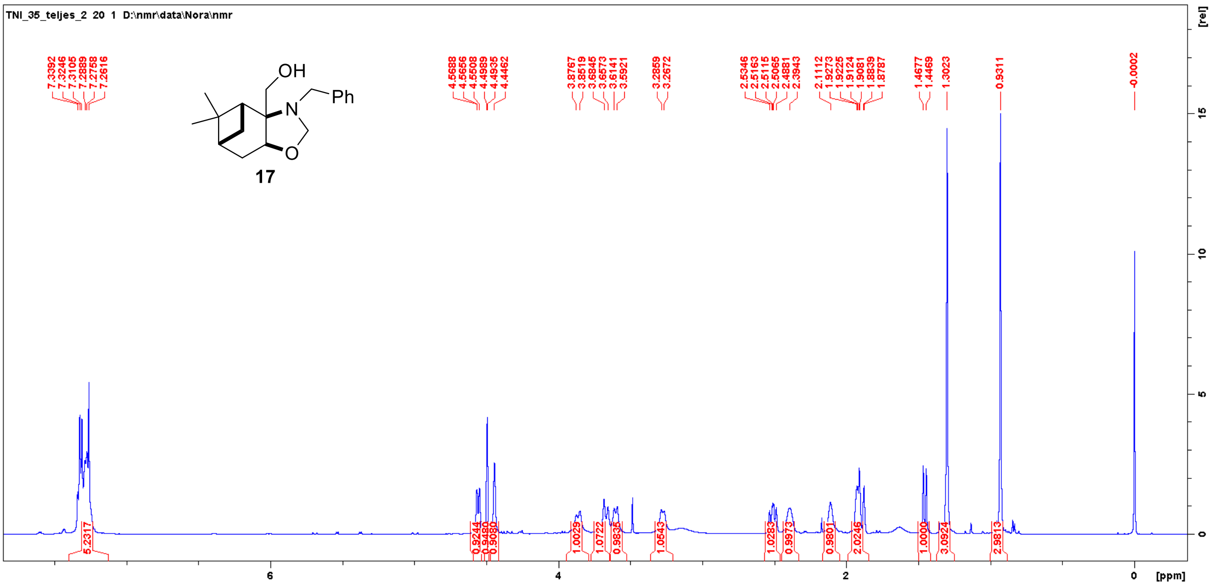

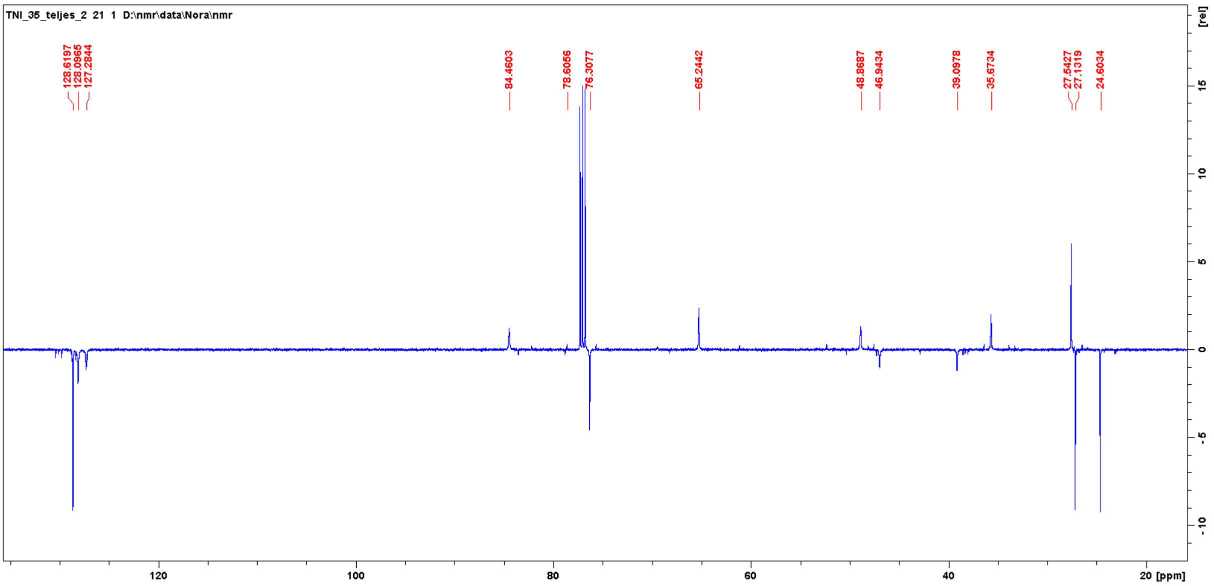

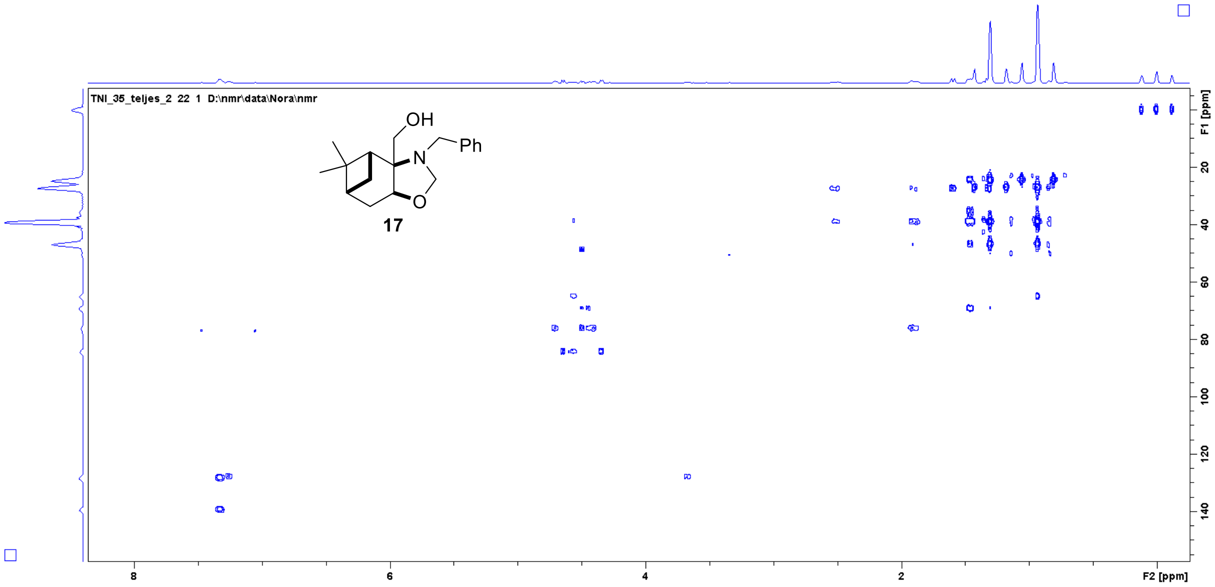
TNI_35_teljes_2 22 1 D:\nmr\data\Nora\nmr
OH
N
Ph
O
17
F1 [ppm]
F2 [ppm]

^{1}H, ^{13}C NMR, COSY, HSQC and HMBC of **18** ($CDCl_3$)

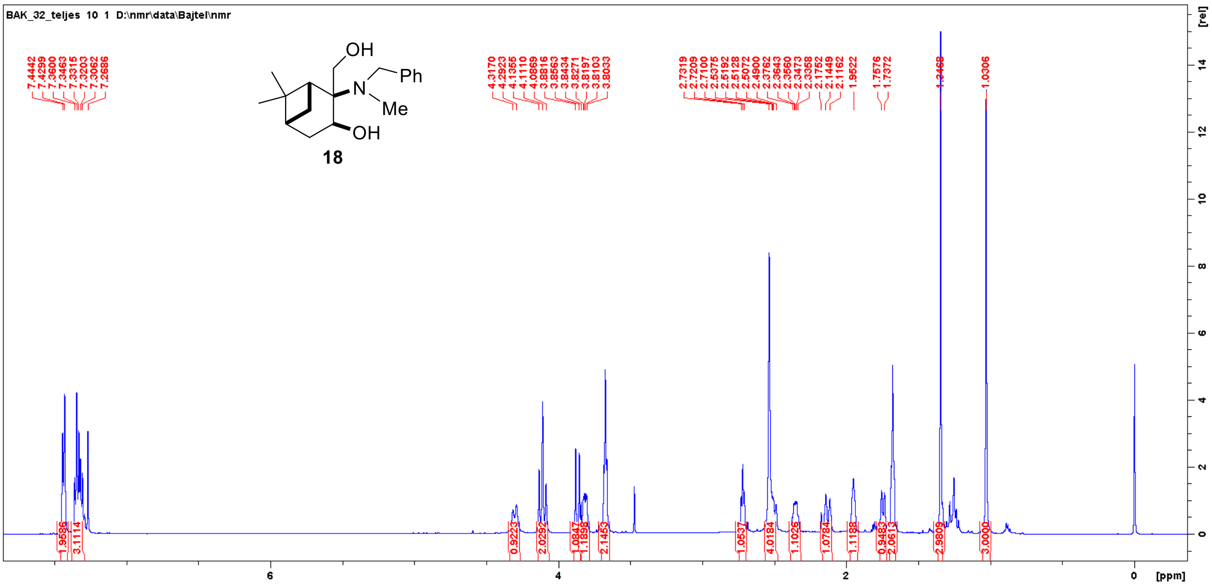

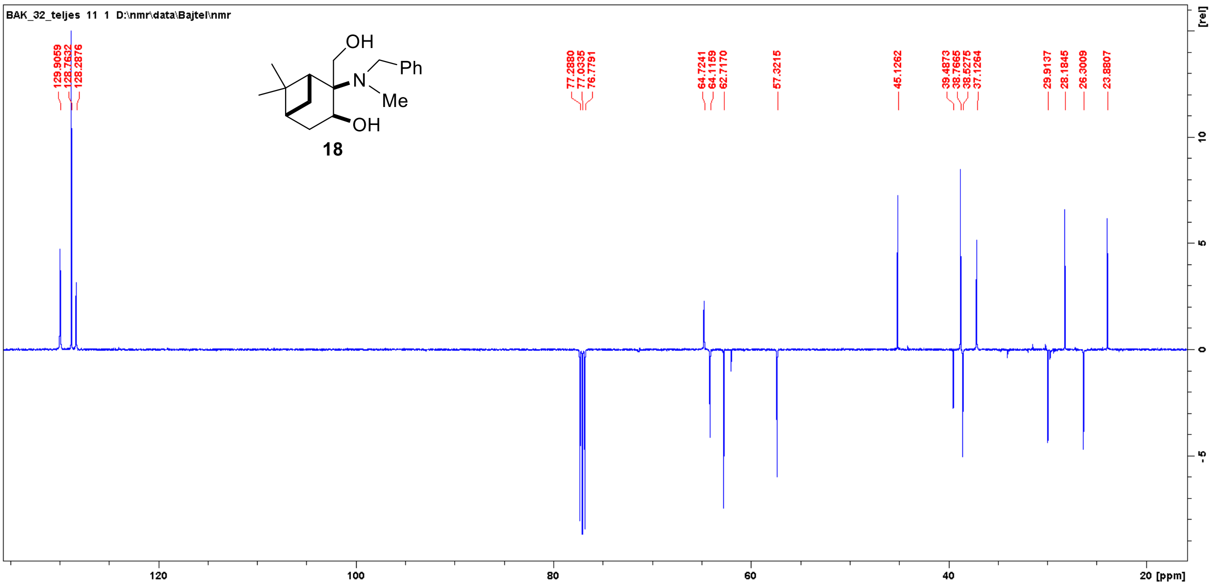

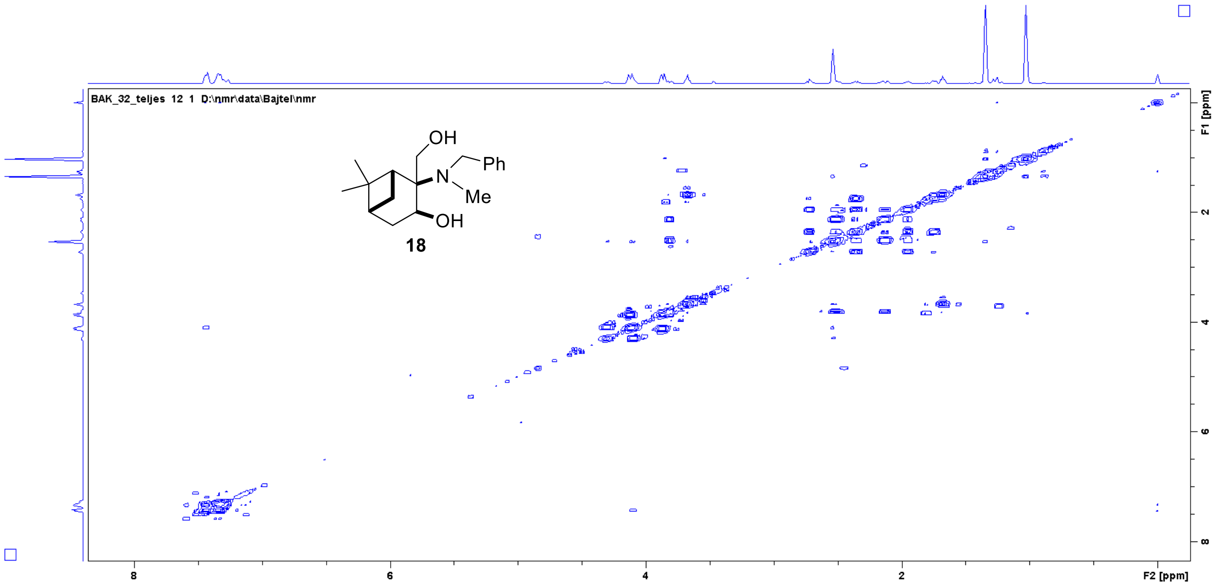

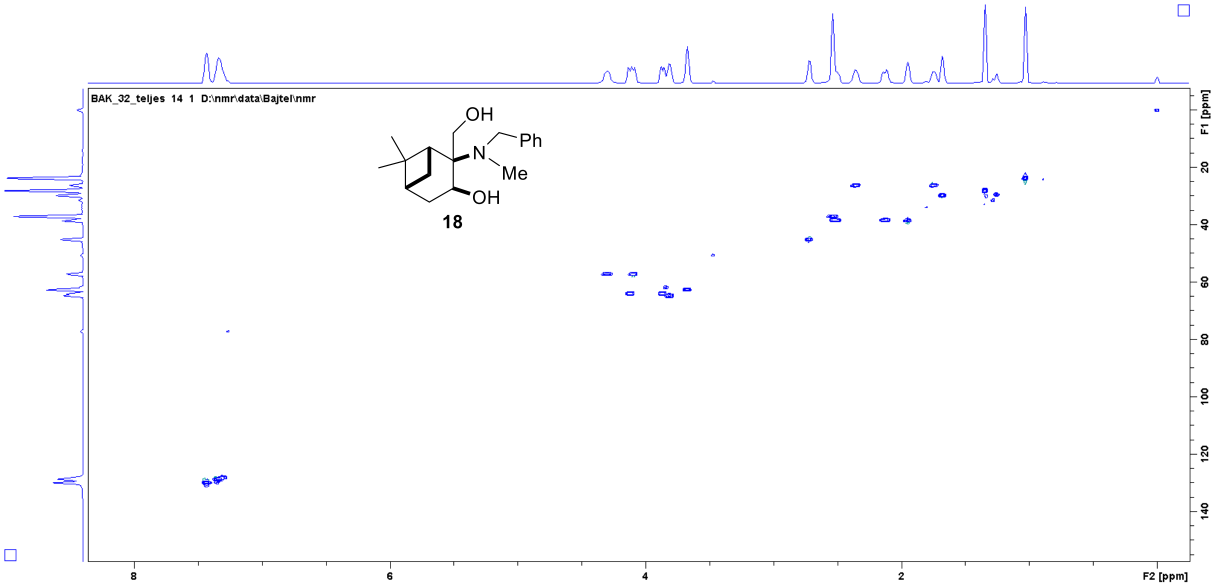
BAK_32_teljes 14 1 D:\nmr\data\Bajtel\nmr
OH
N
Ph
Me
OH
18
F1 [ppm]
F2 [ppm]

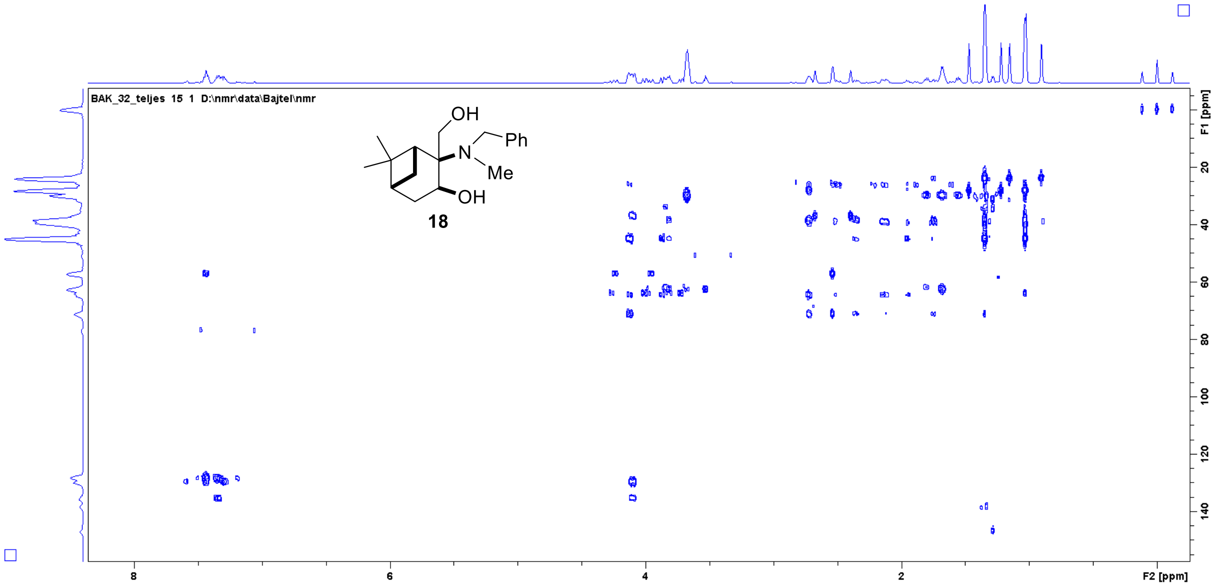
BAK_32_teljes 15 1 D:\nmr\data\Bajtel\nmr
OH
N
Ph
Me
OH
18
F1 [ppm]
F2 [ppm]

^{1}H, ^{13}C NMR, COSY, NOESY, HSQC and HMBC of **19** (DMSO-d_6)

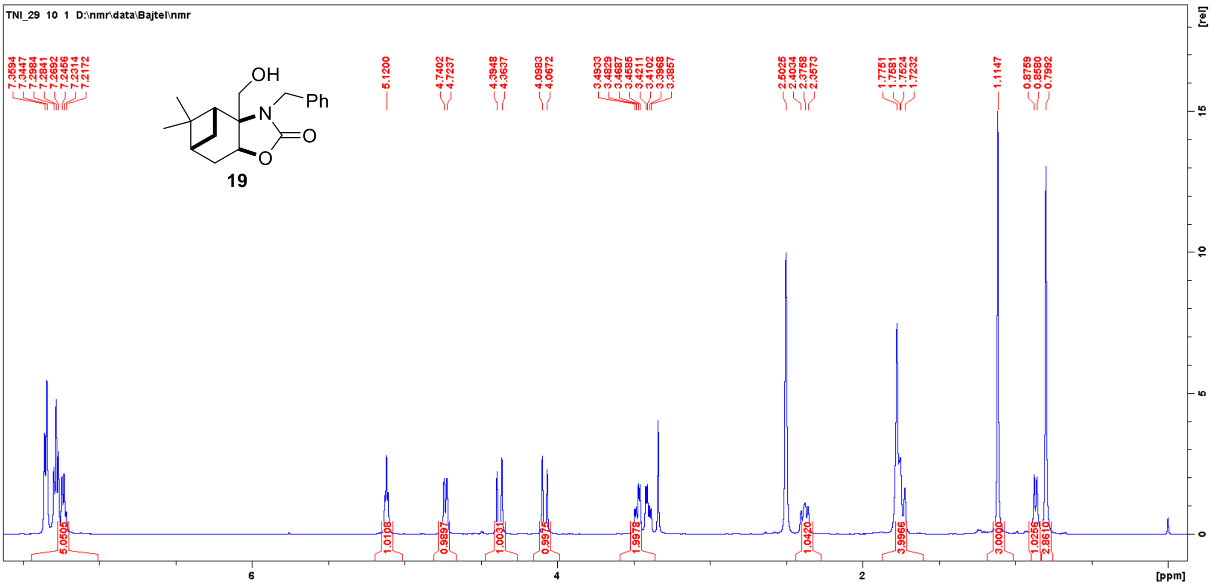

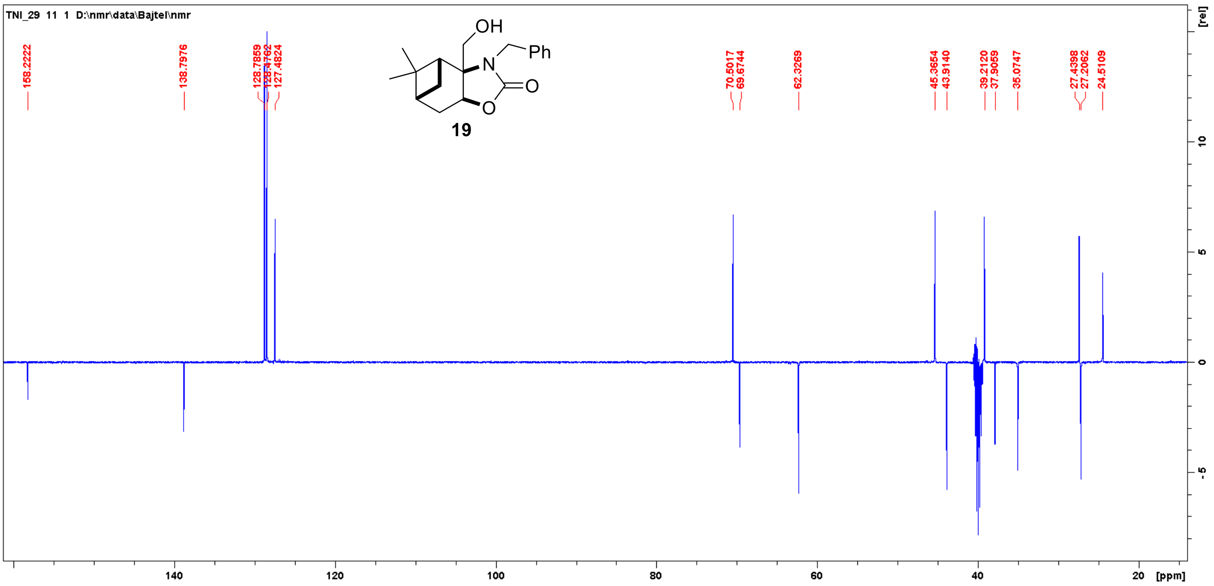

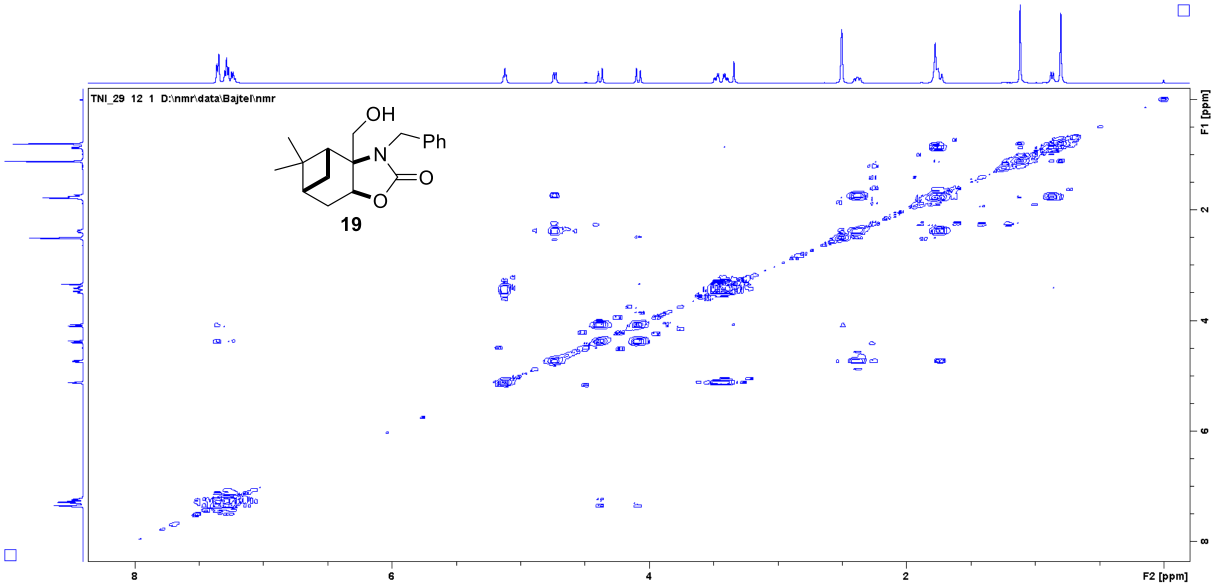
TNI_29 12 1 D:\nmr\data\Bajtel\nmr
OH
N
Ph
O
O
19
F1 [ppm]
F2 [ppm]

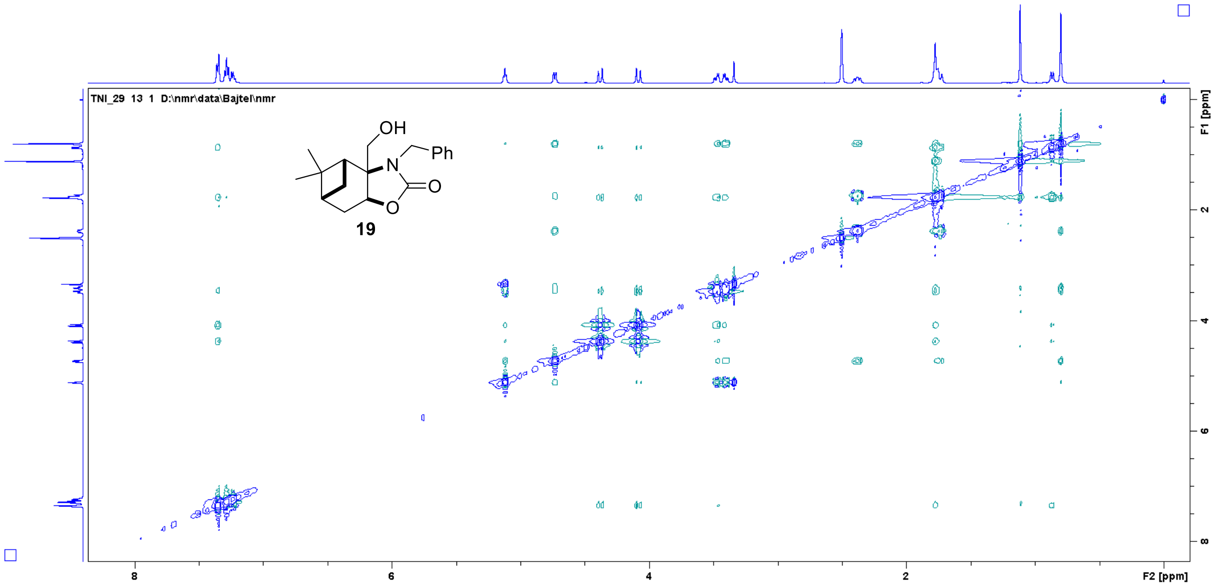
TNI_29 13 1 D:\nmr\data\Bajtel\nmr
OH
N
Ph
O
O
19
F1 [ppm]
F2 [ppm]

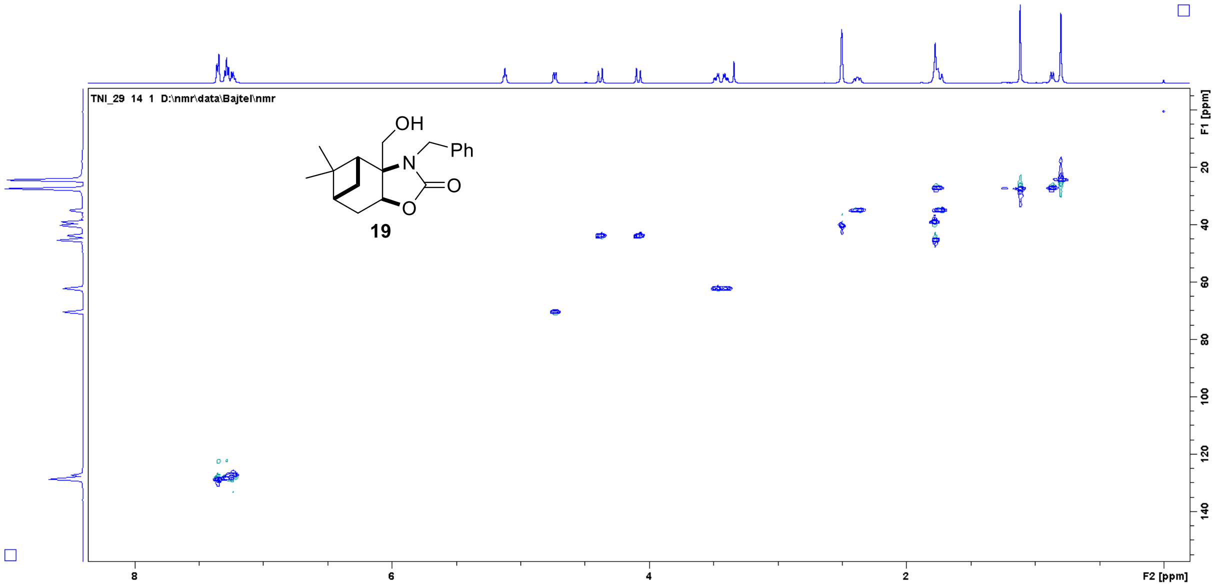
TNI_29 14 1 D:\nmr\data\Bajtel\nmr
OH
N
Ph
O
O
19
F1 [ppm]
F2 [ppm]

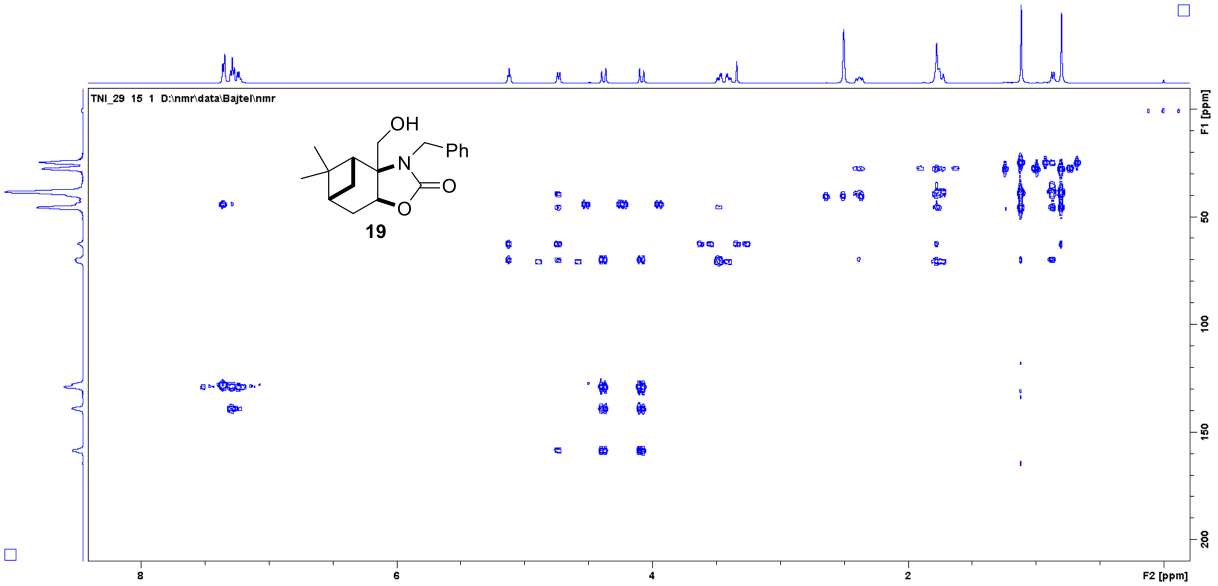
TNI_29 15 1 D:\nmr\data\Bajtel\nmr
OH
N
Ph
O
O
19
F1 [ppm]
F2 [ppm]

^{1}H, ^{13}C NMR, COSY, HSQC and HMBC of **20** ($CDCl_3$)

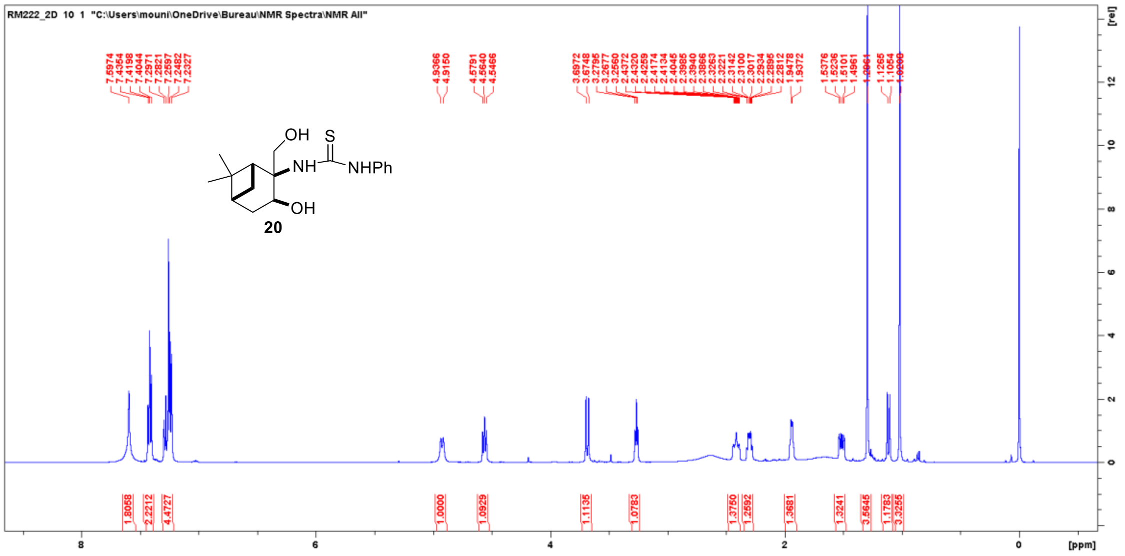

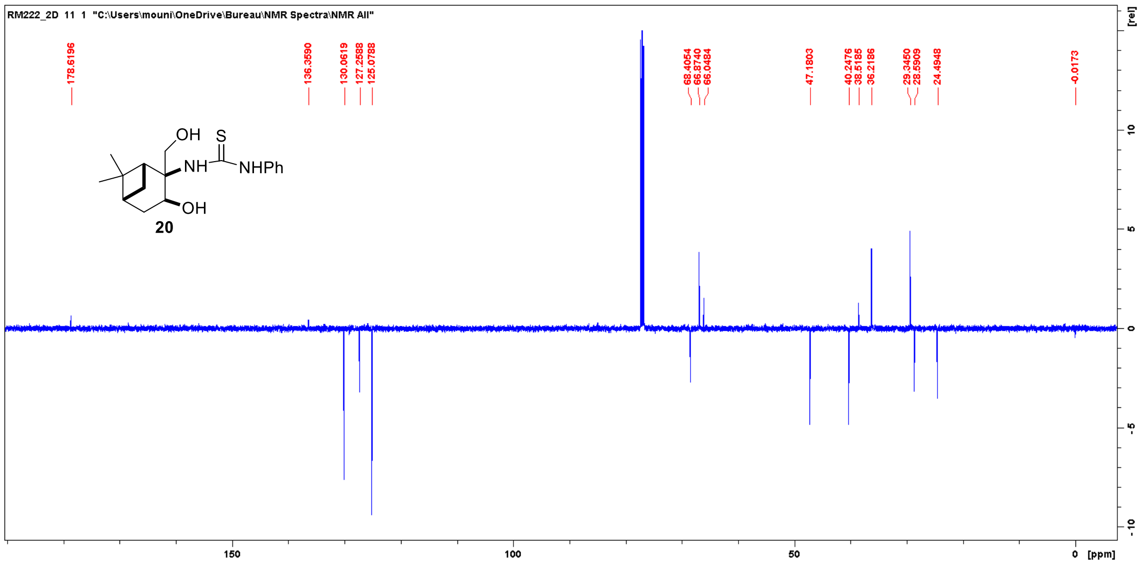

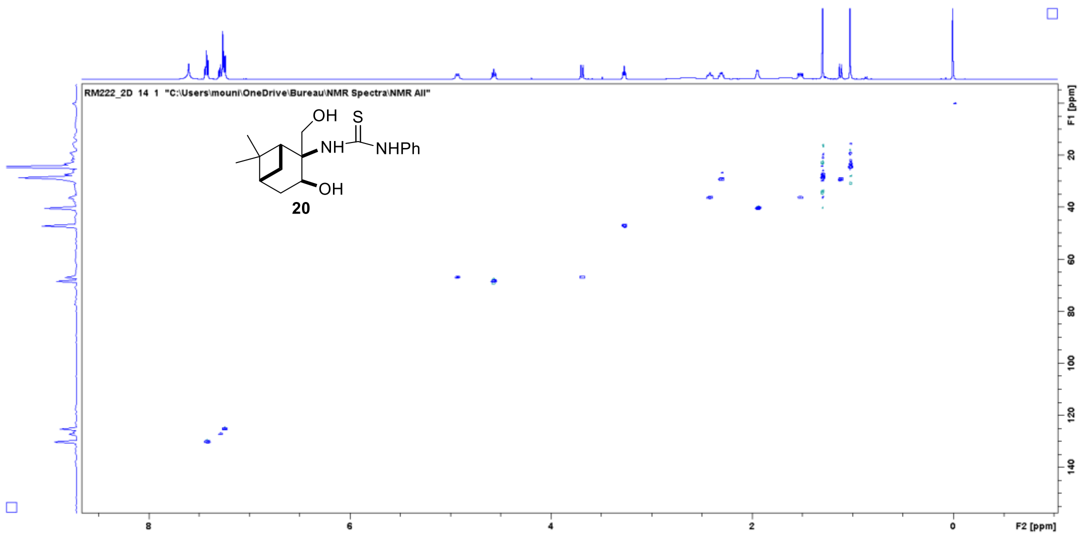

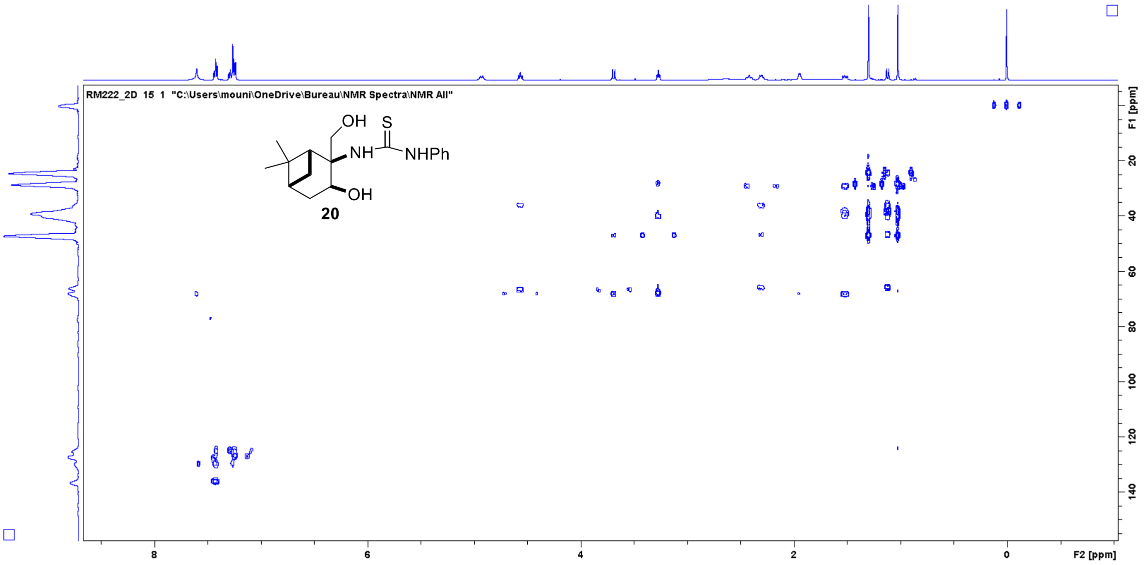
RM222_2D 15 1 "C:\Users\mouni\OneDrive\Bureau\NMR Spectra\NMR All"
OH
S
NH
NHPh
OH
20
F1 [ppm]
F2 [ppm]

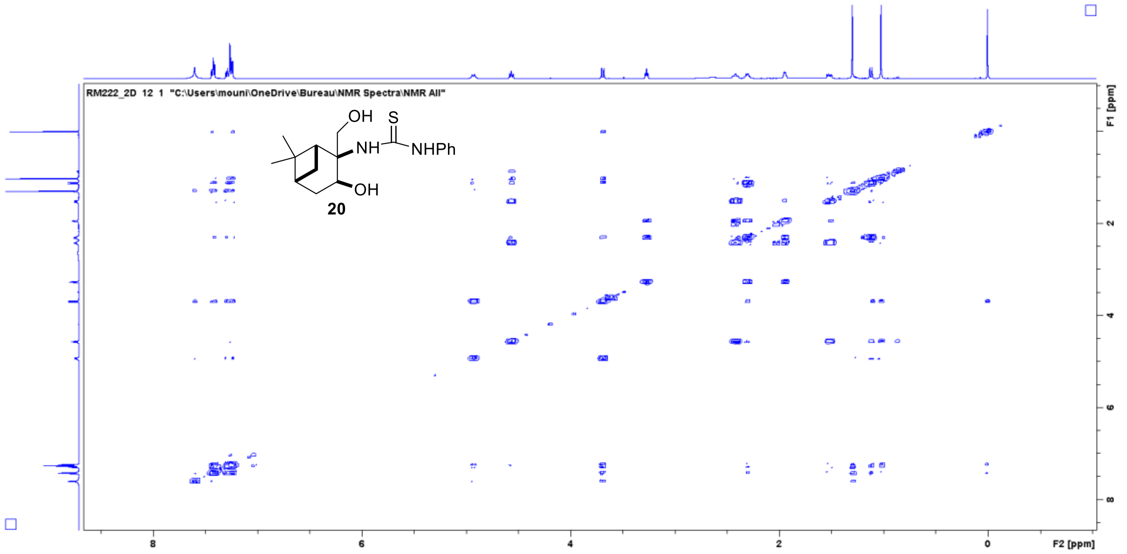
RM222_2D 12 1 "C:\Users\mouni\OneDrive\Bureau\NMR Spectra\NMR All"
OH
S
NH
NHPh
OH
20
F1 [ppm]
F2 [ppm]

^{1}H, ^{13}C NMR, COSY, NOESY, HSQC and HMBC of **21A** ($CDCl_3$)

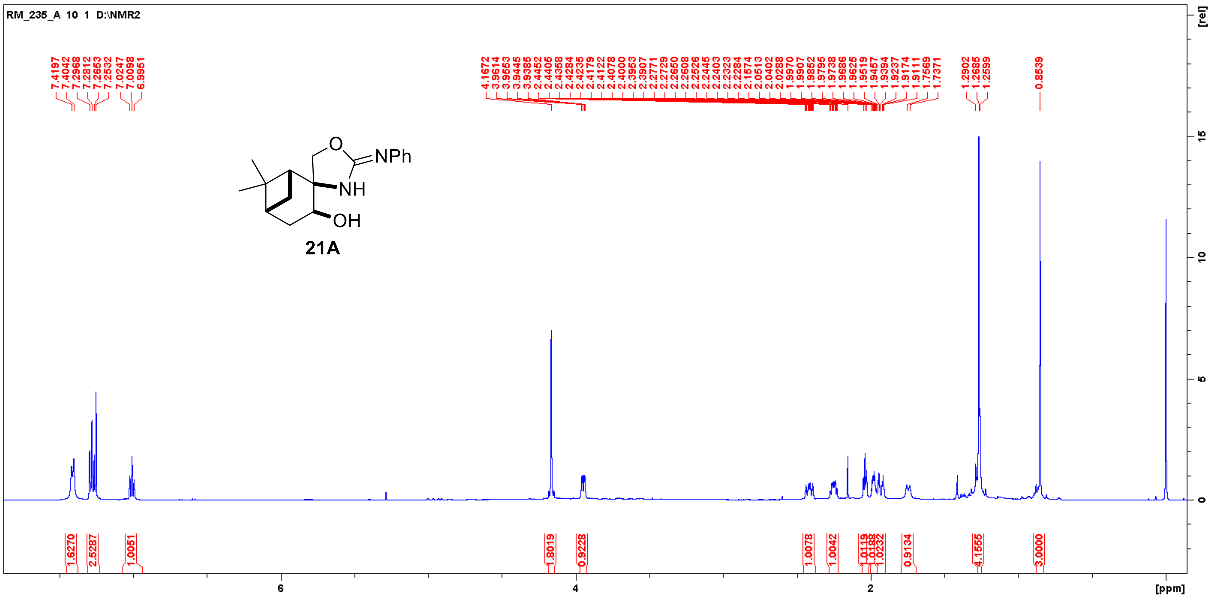

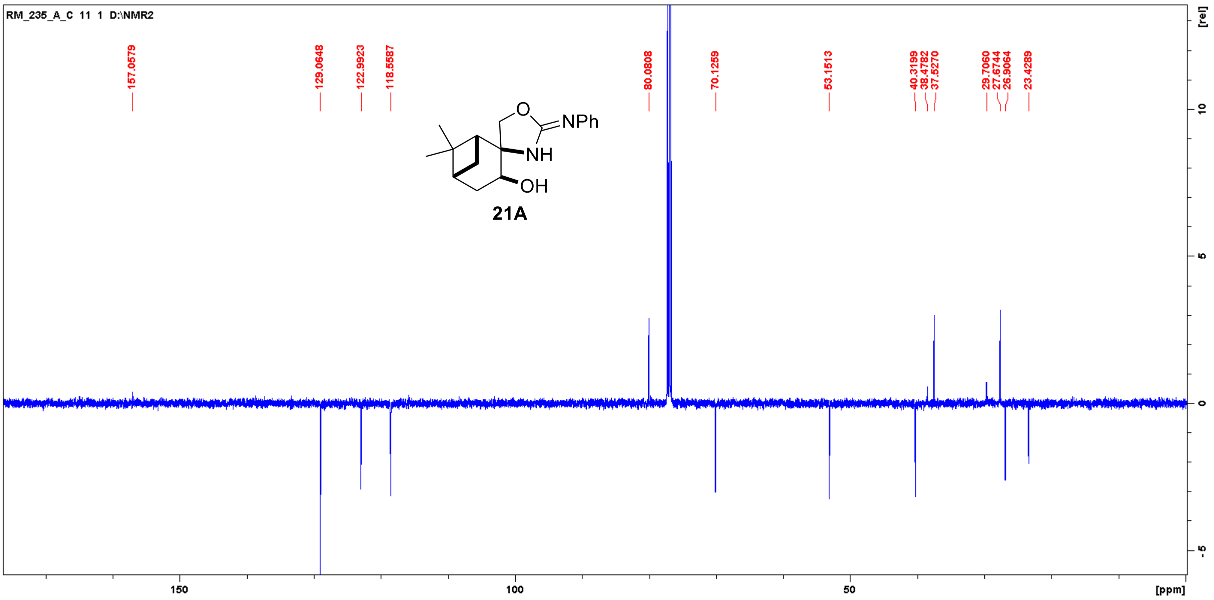

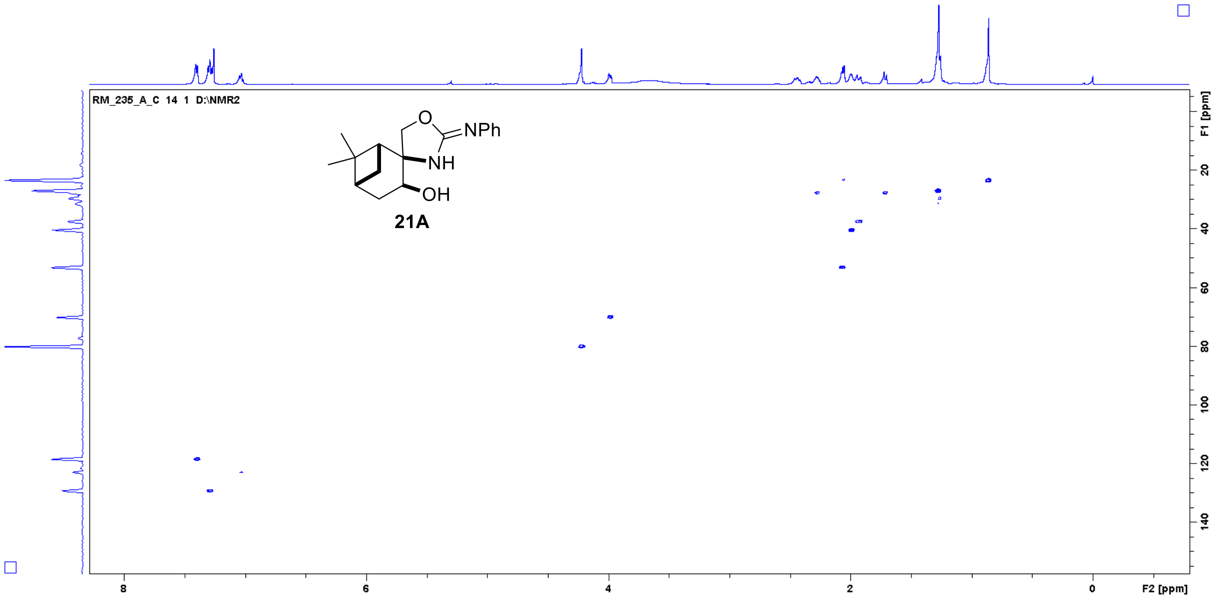
RM_235_A_C 14 1 D:\NMR2
NPh
O
NH
OH
21A
F1 [ppm]
F2 [ppm]

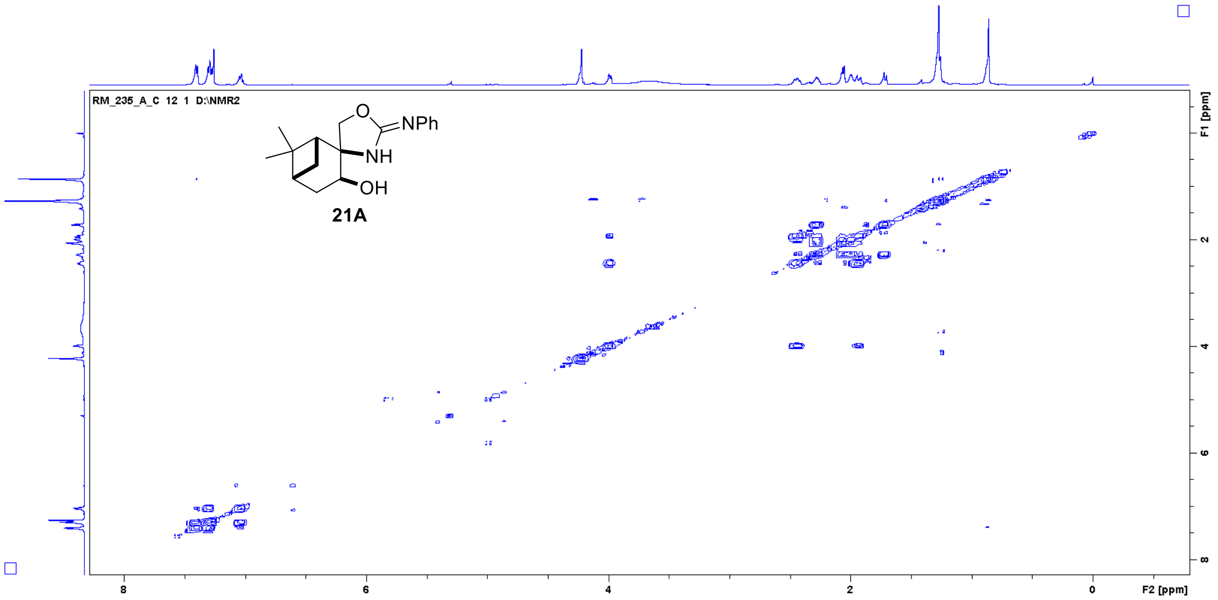
RM_235_A_C 12 1 D:\NMR2
NPh
O
NH
OH
21A
F1 [ppm]
F2 [ppm]

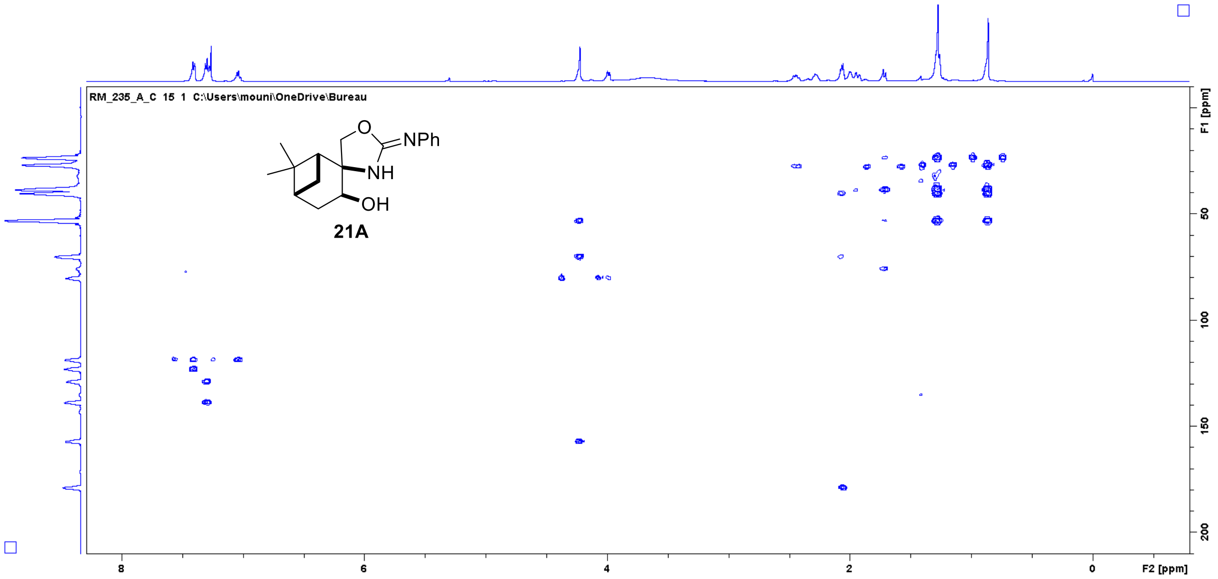
RM_235_A_C 15 1 C:\Users\mouni\OneDrive\Bureau
NPh
O
NH
OH
21A
F1 [ppm]
F2 [ppm]

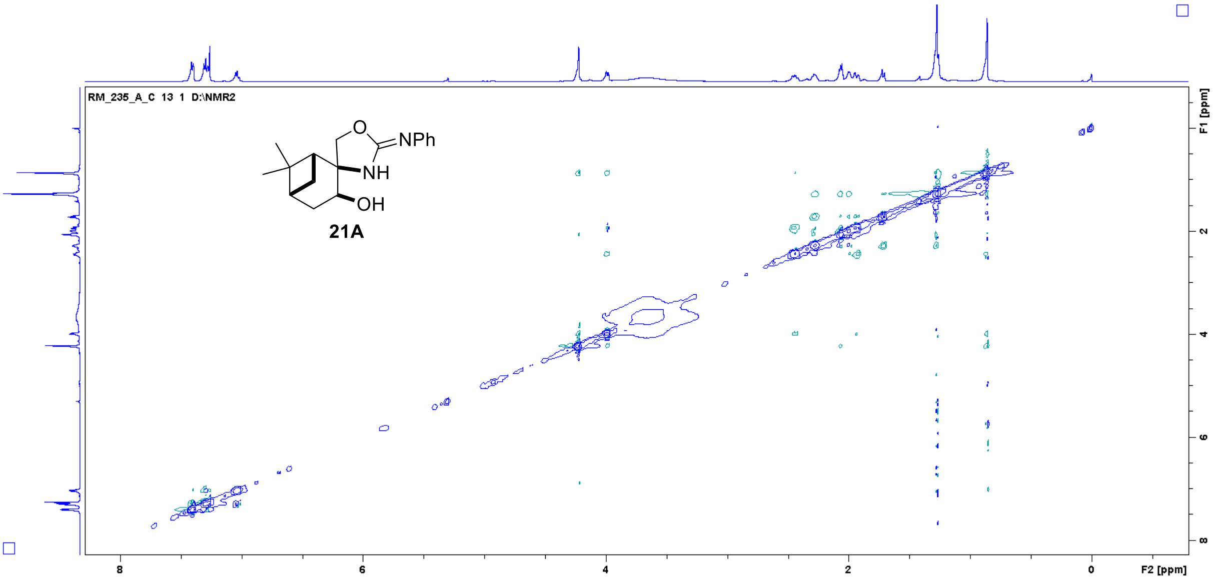
RM_235_A_C 13 1 D:\NMR2
O
NPh
NH
OH
21A
F1 [ppm]
F2 [ppm]
8
6
4
2
0

^{1}H, ^{13}C NMR, COSY,NOESY, HSQC and HMBC of **15A-E** ($CDCl_3$)

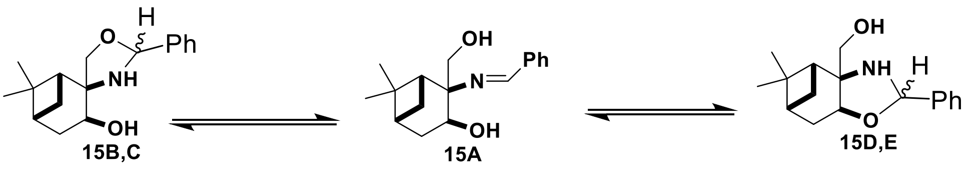

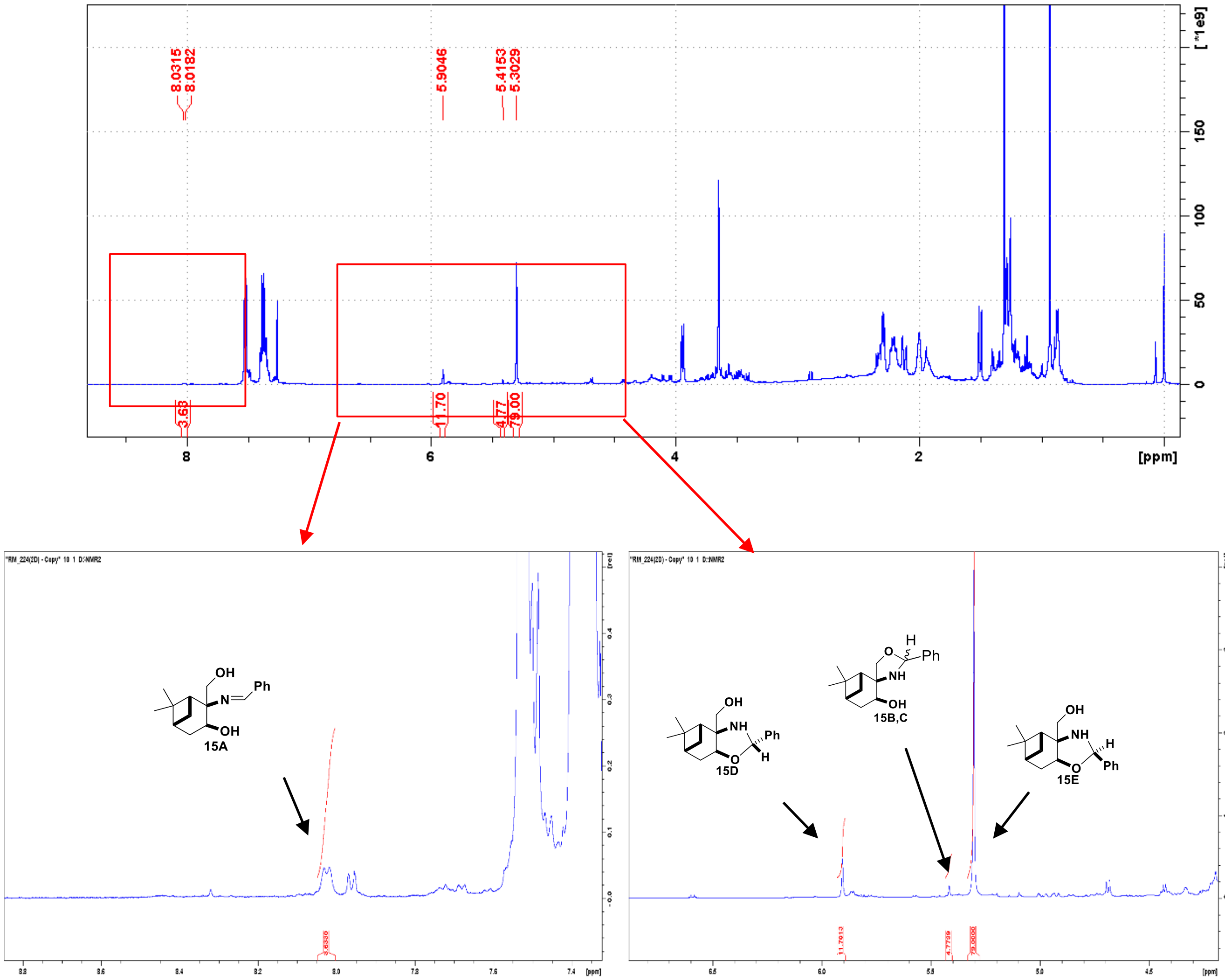

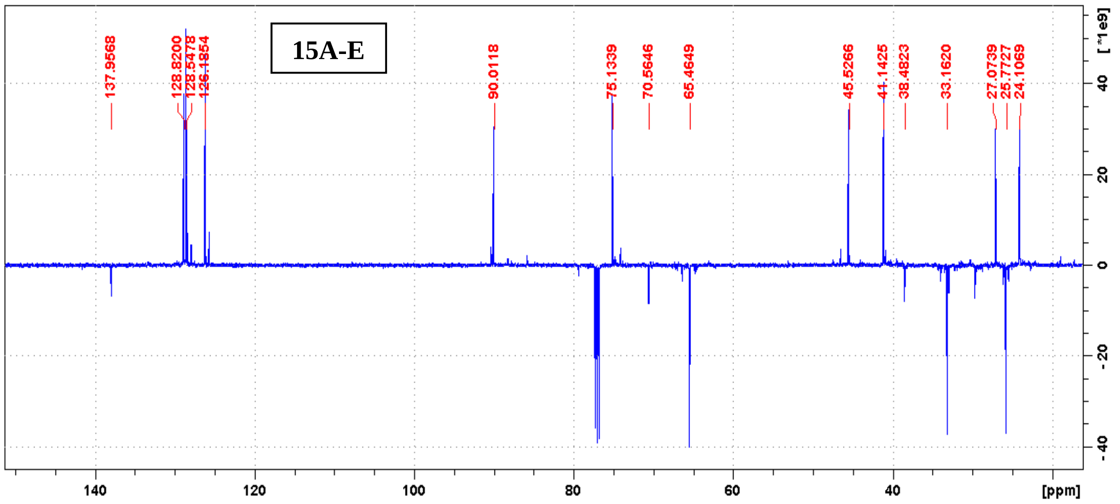
15A-E
137.9568
128.8200
128.5478
126.1854
90.0118
75.1339
70.5646
65.4649
45.5266
41.1425
38.4823
33.1620
27.0739
25.7727
24.1069
[*1e9]
[ppm]

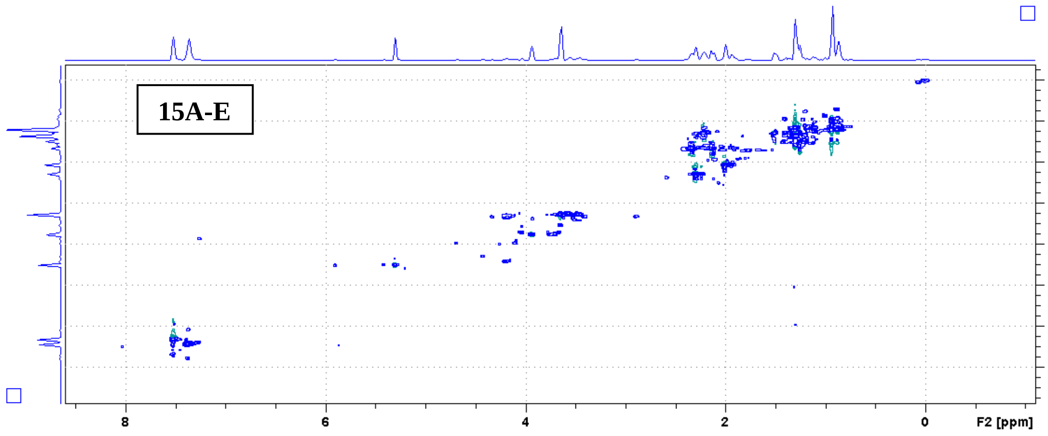
15A-E
F1 [ppm]
F2 [ppm]

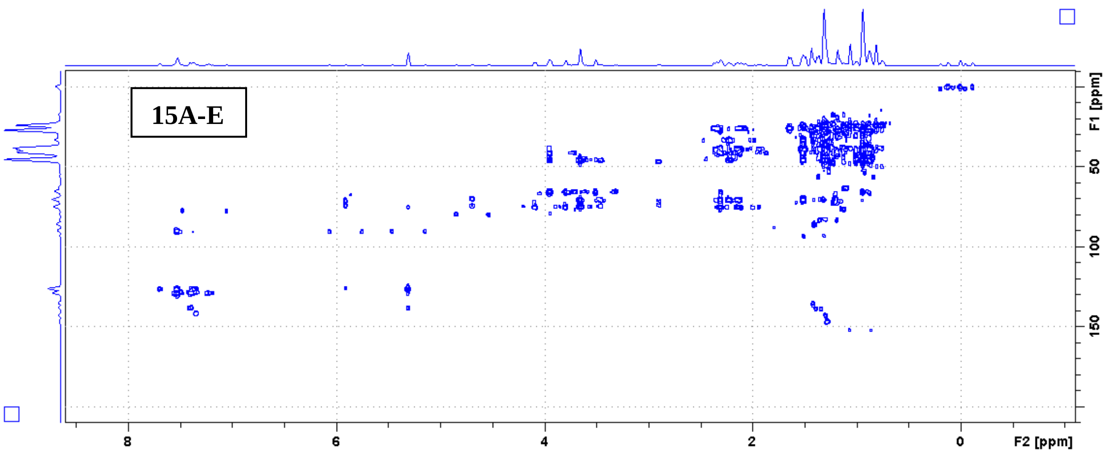
15A-E
F1 [ppm]
F2 [ppm]

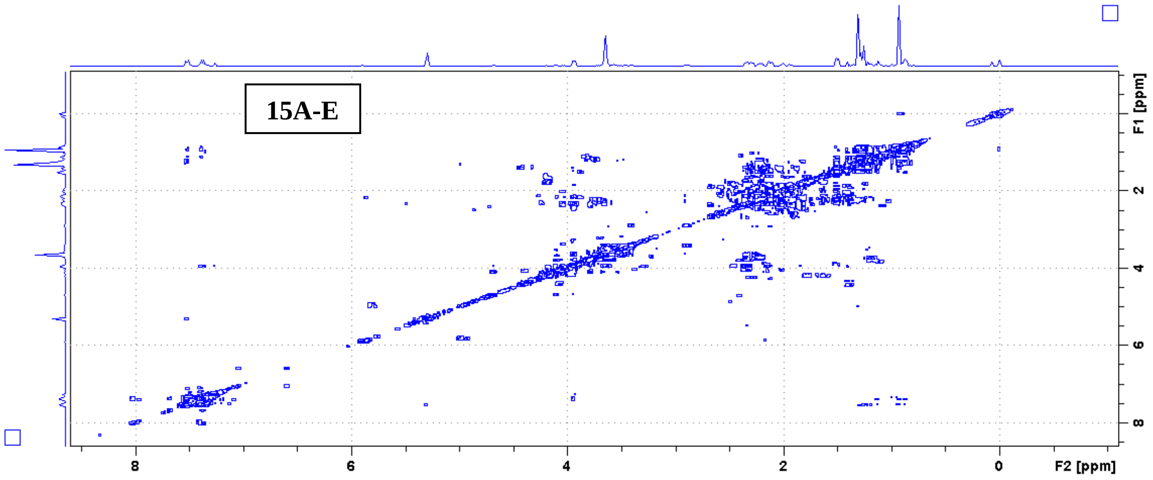
15A-E
F1 [ppm]
F2 [ppm]
8
6
4
2
0

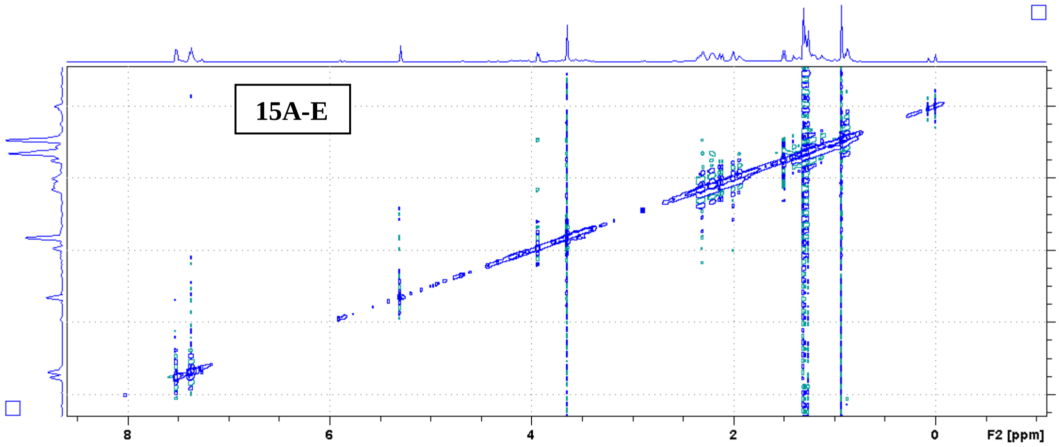
15A-E
F1 [ppm]
F2 [ppm]
8
6
4
2
0

^{1}H, ^{13}C NMR, HSQC, HMBC, COSY and NOESY of **21B** ($CDCl_3$)

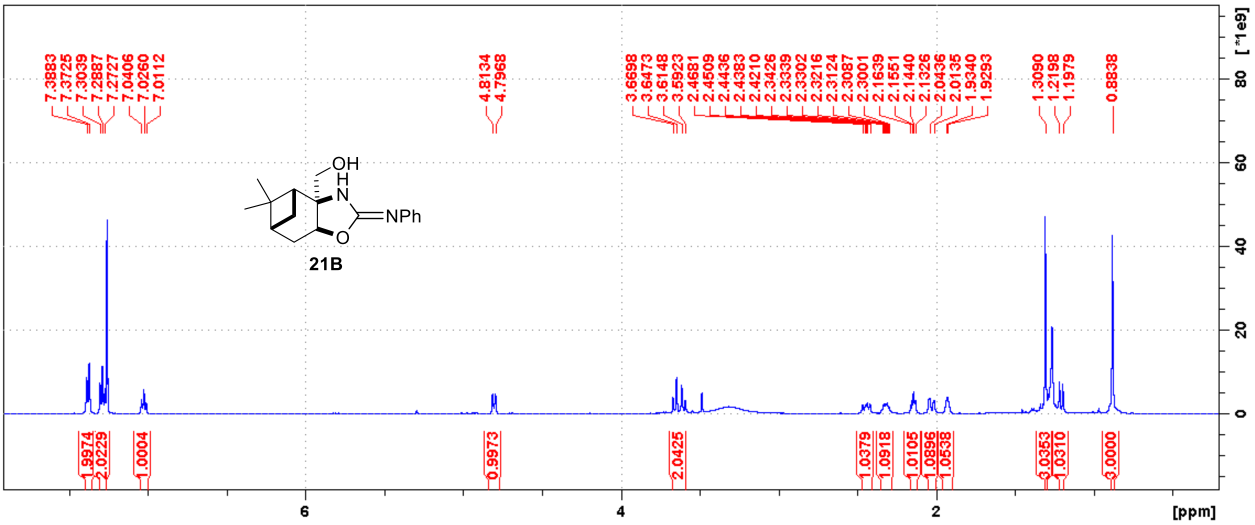

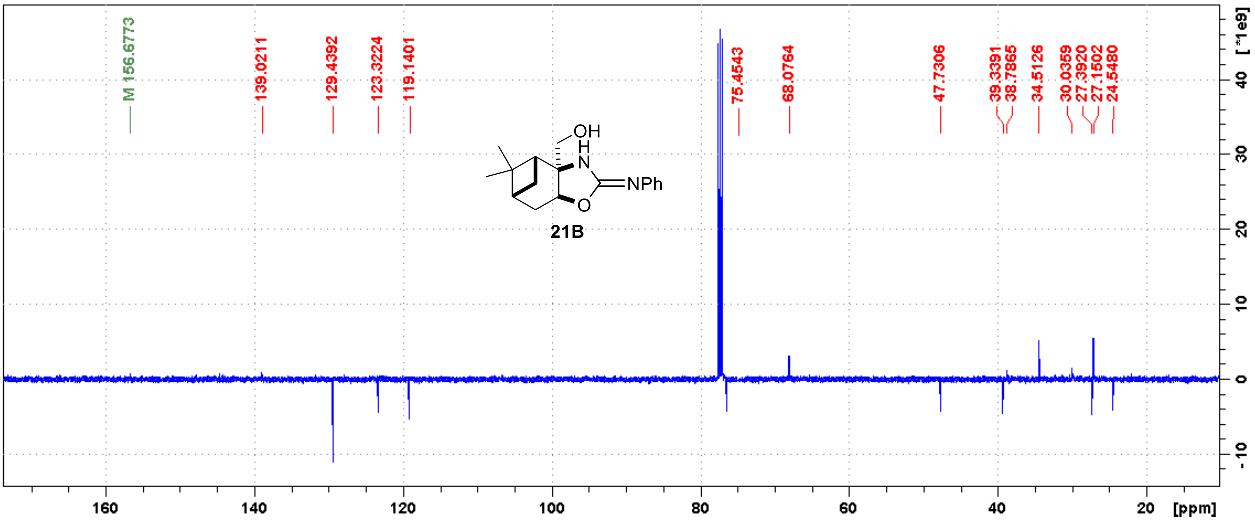

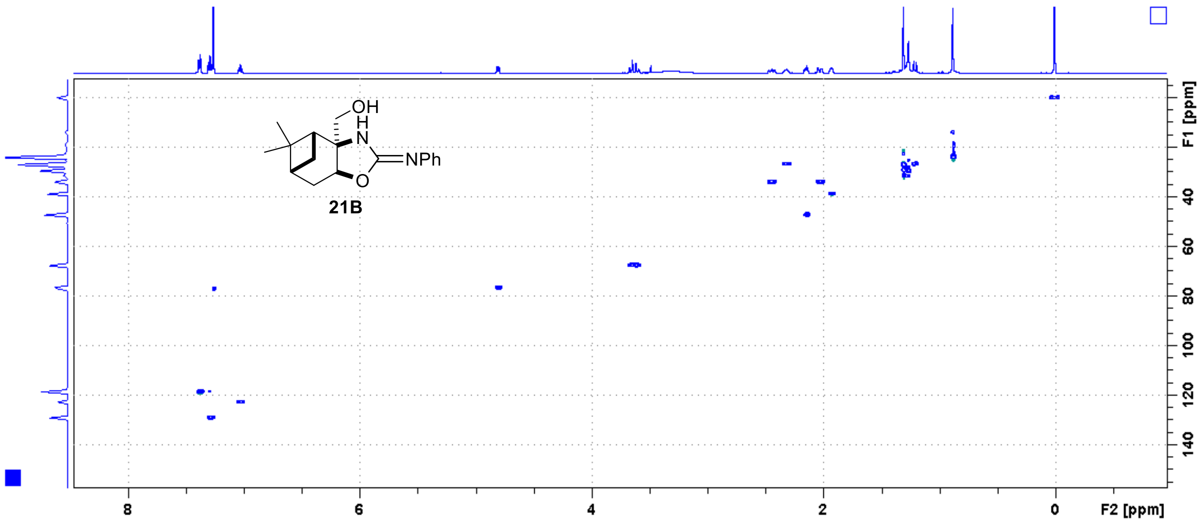

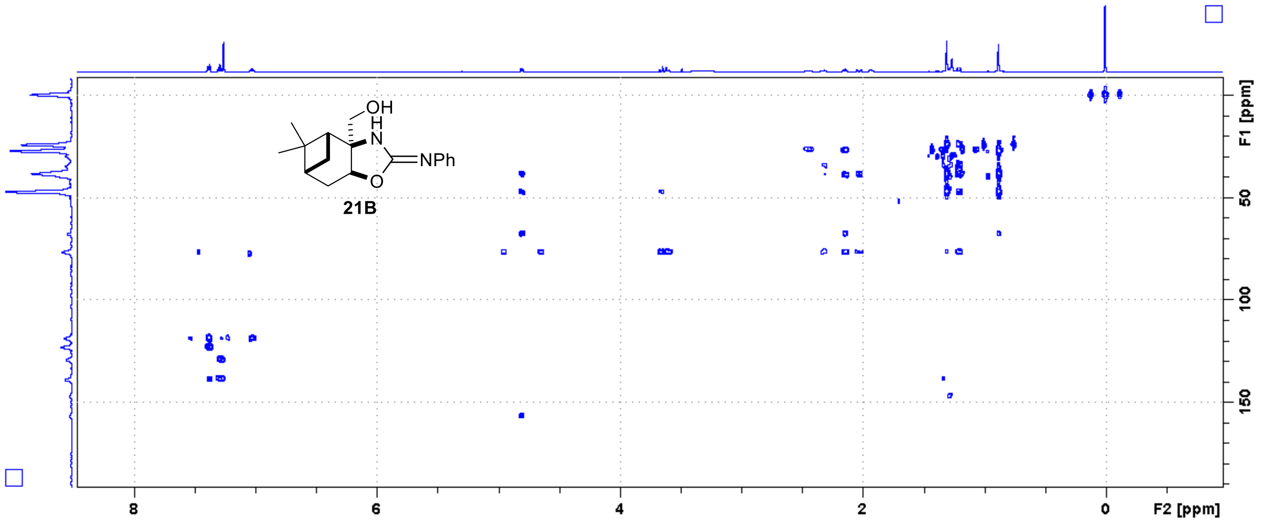
OH
H
N
NPh
O
21B
F1 [ppm]
50
100
150
8
6
4
2
0
F2 [ppm]

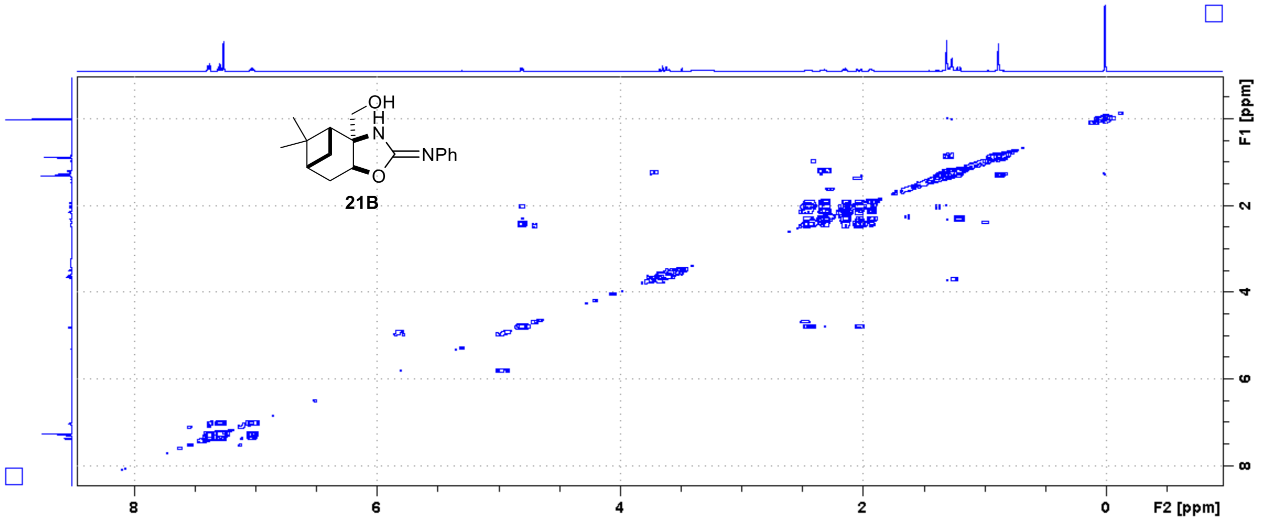
OH
H
N
NPh
O
21B
F1 [ppm]
2
4
6
8
8
6
4
2
0
F2 [ppm]

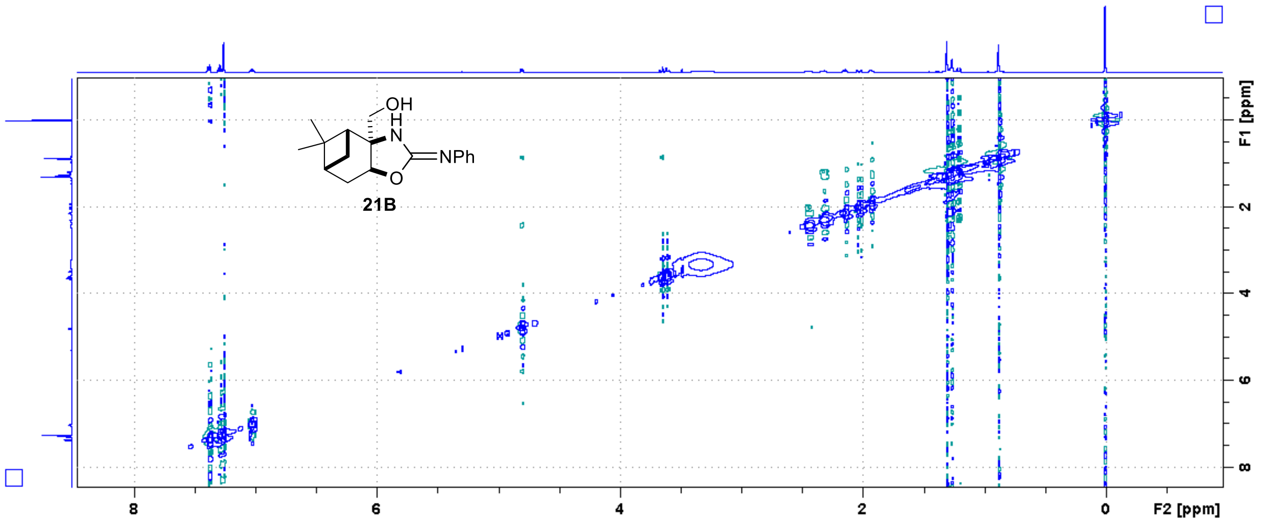
OH
H
N
NPh
O
21B
F1 [ppm]
2
4
6
8
8
6
4
2
0
F2 [ppm]

Time-dependent tautomerisation of **21A** to **21B** in $CDCl_3$ solution

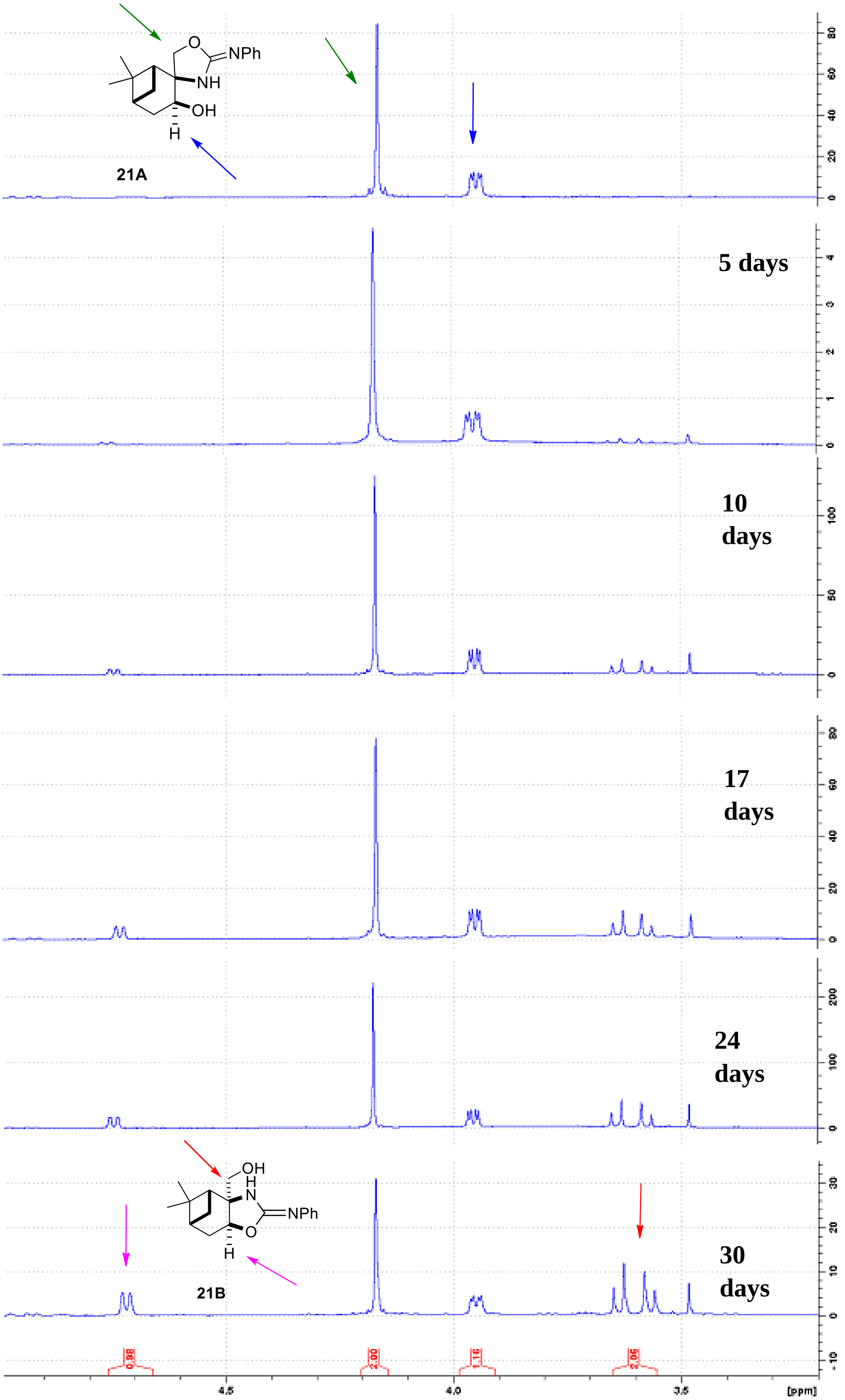

Supplement: File 1 — Experimental part, analytical data, NMR spectra and X-ray data of the prepared compounds. [file Beilstein_J_Org_Chem-17-983-s001.pdf]
